# Cereal grain mineral micronutrient and soil chemistry data from GeoNutrition surveys in Ethiopia and Malawi

**DOI:** 10.1038/s41597-022-01500-5

**Published:** 2022-07-25

**Authors:** D. B. Kumssa, A. W. Mossa, T. Amede, E. L. Ander, E. H. Bailey, L. Botoman, C. Chagumaira, J. G. Chimungu, K. Davis, S. Gameda, S. M. Haefele, K. Hailu, E. J. M. Joy, R. M. Lark, I. S. Ligowe, S. P. McGrath, A. Milne, P. Muleya, M. Munthali, E. Towett, M. G. Walsh, L. Wilson, S. D. Young, I. R. Haji, M. R. Broadley, D. Gashu, P. C. Nalivata

**Affiliations:** 1 School of Biosciences, University of Nottingham, Sutton Bonington Campus, Loughborough, LE12 5RD UK; 2 International Crop Research Institute for the Semi-Arid Tropics (ICRISAT), ILRI Sholla Campus, P.O. Box 5689, Addis Ababa, Ethiopia; 3 Centre for Environmental Geochemistry, British Geological Survey, Keyworth, Nottinghamshire NG12 5GG UK; 4 Lilongwe University of Agriculture and Natural Resources (LUANAR), Bunda College, P.O. Box 219, Lilongwe, Malawi; 5 The Department of Agricultural Research Services, P.O. Box 30779, Lilongwe, Malawi; 6 Future Food Beacon, University of Nottingham, Sutton Bonington Campus, Nottinghamshire, LE12 5RD UK; 7 Rothamsted Research, Harpenden, Hertfordshire, AL5 2JQ UK; 8 International Maize and Wheat Improvement Centre (CIMMYT), ILRI Sholla Campus, P.O. Box 5689, Addis Ababa, Ethiopia; 9 Centre for Food Science and Nutrition, Addis Ababa University, P.O. Box 1176, Addis Ababa, Ethiopia; 10 Addis Ababa Science and Technology University, Addis Ababa, Ethiopia; 11 Faculty of Epidemiology and Population Health, London School of Hygiene & Tropical Medicine, Keppel Street, London, WC1E 7HT UK; 12 World Agroforestry (ICRAF), United Nations Avenue, P.O. Box 30677, Nairobi, Kenya; 13 Africa Soil Information Service, Selian Agricultural Research Institute, P.O. Box 2704, Arusha, Tanzania

**Keywords:** Biogeochemistry, Metabolomics

## Abstract

The dataset comprises primary data for the concentration of 29 mineral micronutrients in cereal grains and up to 84 soil chemistry properties from GeoNutrition project surveys in Ethiopia and Malawi. The work provided insights on geospatial variation in the micronutrient concentration in staple crops, and the potential influencing soil factors. In Ethiopia, sampling was conducted in Amhara, Oromia, and Tigray regions, during the late-2017 and late-2018 harvest seasons. In Malawi, national-scale sampling was conducted during the April–June 2018 harvest season. The concentrations of micronutrients in grain were measured using inductively coupled plasma mass spectrometry (ICP-MS). Soil chemistry properties reported include soil pH; total soil nitrogen; total soil carbon (C); soil organic C; effective cation exchange capacity and exchangeable cations; a three-step sequential extraction scheme for the fractionation of sulfur and selenium; available phosphate; diethylenetriaminepentaacetic acid (DTPA)-extractable trace elements; extractable trace elements using 0.01 M Ca(NO_3_)_2_ and 0.01 M CaCl_2_; and isotopically exchangeable Zn. These data are reported here according to FAIR data principles to enable users to further explore agriculture-nutrition linkages.

## Abstract (summary)

**Table:** 

Measurement(s)	Trace Element • soil chemical properties
Technology Type(s)	Inductively-Coupled Plasma Mass Spectrometry
Factor Type(s)	Geography • Staple cereal crop
Sample Characteristic - Organism	Staple cereal food crops
Sample Characteristic - Environment	Smallholder farming
Sample Characteristic - Location	Ethiopia • Malawi

## Background & Summary

Micronutrients are the vitamins and minerals that our bodies require in small amounts, and which are obtained from the food we eat. Micronutrient deficiencies (MNDs) among people remain a major global concern; more than 2 billion people are likely to be affected worldwide, with greater deficiency risks in sub-Saharan Africa than in most other regions^1^. The risk of MNDs in populations can be informed by understanding the supply of micronutrients within food systems. This approach has typically been conducted at a national level, based on secondary interpretation of food consumption, expenditure or supply data from household surveys and food balance sheets^1–5^.

Recent studies from Ethiopia and Malawi have reported substantial variation in the micronutrient concentration of the grains of staple cereal crops at subnational levels, including for calcium (Ca), iron (Fe), selenium (Se) and zinc (Zn)^6–8^. Some of this variation is spatially correlated at distances of up to several hundred kilometres. What this means is that for people consuming food sourced locally, as is the case for many smallholder farming communities, the location of residence will be a major (sometimes the largest) influencing factor in determining the dietary intake of micronutrients from cereals^7^. Furthermore, for the micronutrient Se, there is strong evidence of linkages between soil and landscape features, cereal grain concentrations, and biomarkers of Se status in people^9,10^.

Here we report the wider set of primary data for cereal grains and soils from these studies in Ethiopia and Malawi (Tables 1 and 2), with a focus on the data reported in Gashu *et al*.^6,7^, Mossa *et al*.^11^, and Botoman *et al*.^12^. These data were obtained as part of ongoing work within two ‘GeoNutrition’ projects, funded primarily by the Bill & Melinda Gates Foundation (BMGF) and the UK Government’s Global Challenges Research Fund (GCRF). Soil and grain samples were collected in both countries using spatially balanced sampling designs, along with meta-data, with the informed consent of farmers.

**Table 1:** Type and number of cereal grain samples collected from Ethiopia and Malawi.

Cereal grain	Number of samples	
	Ethiopia	Malawi
Barley	175	0
Finger millet	37	1
Maize	290	1,608
Pearl millet	1	32
Rice	8	54
Sorghum	135	117
Teff	362	0
Triticale	19	0
Wheat	325	0
**Total grain-soil pairs**	**1,352**	**1,812**

The total row indicates the total number of cereal grain-soil sample pairs sampled in each country.

**Table 2:** Elemental concentrations in cereal grains reported from Ethiopia and Malawi.

Data field name	Element	Data field name	Element
**Ag_grain**	Silver	**Mg_grain**	Magnesium
**Al_grain**	Aluminium	**Mn_grain**	Manganese
**As_grain**	Arsenic	**Mo_grain**	Molybdenum
**B_grain**	Boron	**Ni_grain**	Nickel
**Ba_grain**	Barium	**P_grain**	Phosphorus
**Be_grain**	Beryllium	**Pb_grain**	Lead
**Ca_grain**	Calcium	**Rb_grain**	Rubidium
**Cd_grain**	Cadmium	**S_grain**	Sulfur
**Co_grain**	Cobalt	**Se_grain**	Selenium
**Cr_grain**	Chromium	**Sr_grain**	Strontium
**Cs_grain**	Caesium	**Tl_grain**	Thallium
**Cu_grain**	Copper	**U_grain**	Uranium
**Fe_grain**	Iron	**V_grain**	Vanadium
**K_grain**	Potassium	**Zn_grain**	Zinc
**Li_grain**	Lithium		

All concentrations are in mg kg^−1^ on dry matter basis.

## Methods

### Research design

#### Ethical approval

The research work that generated these data involved metadata collection using semi-structured questionnaires, and sampling of cereal grain from farmers’ fields or grain stores and soils from the corresponding crop fields with the prior informed consent of the farmers. Farmers who participated in the survey received an information sheet (see Supplementary files 1 and 2) explaining the details of the project, what participation would involve, and how their data would be used, including its eventual release. The work was conducted under ethical approvals from the University of Nottingham, School of Sociology and Social Policy Research Ethics Committee (REC); BIO-1819-001 and BIO-1718-0004 for Ethiopia and Malawi, respectively. These REC approvals were recognized formally by the Directors of Research at Addis Ababa University (Ethiopia) and Lilongwe University of Agriculture and Natural Resources (Malawi), who also reviewed the study protocols.

#### Sampling design

The sampling design is described in full by Gashu *et al*.^7^. The objective of the sampling was to support the evaluation of relationships between crop and soil properties, and spatial mapping of these. For this reason, the basic sample design was selected to achieve spatial coverage of the agreed sample frame. A random subset of the spatial coverage sample was then selected, and an additional close paired sample site was specified for each of these, to support statistical modelling of spatial variation.

**Fig. 1:**
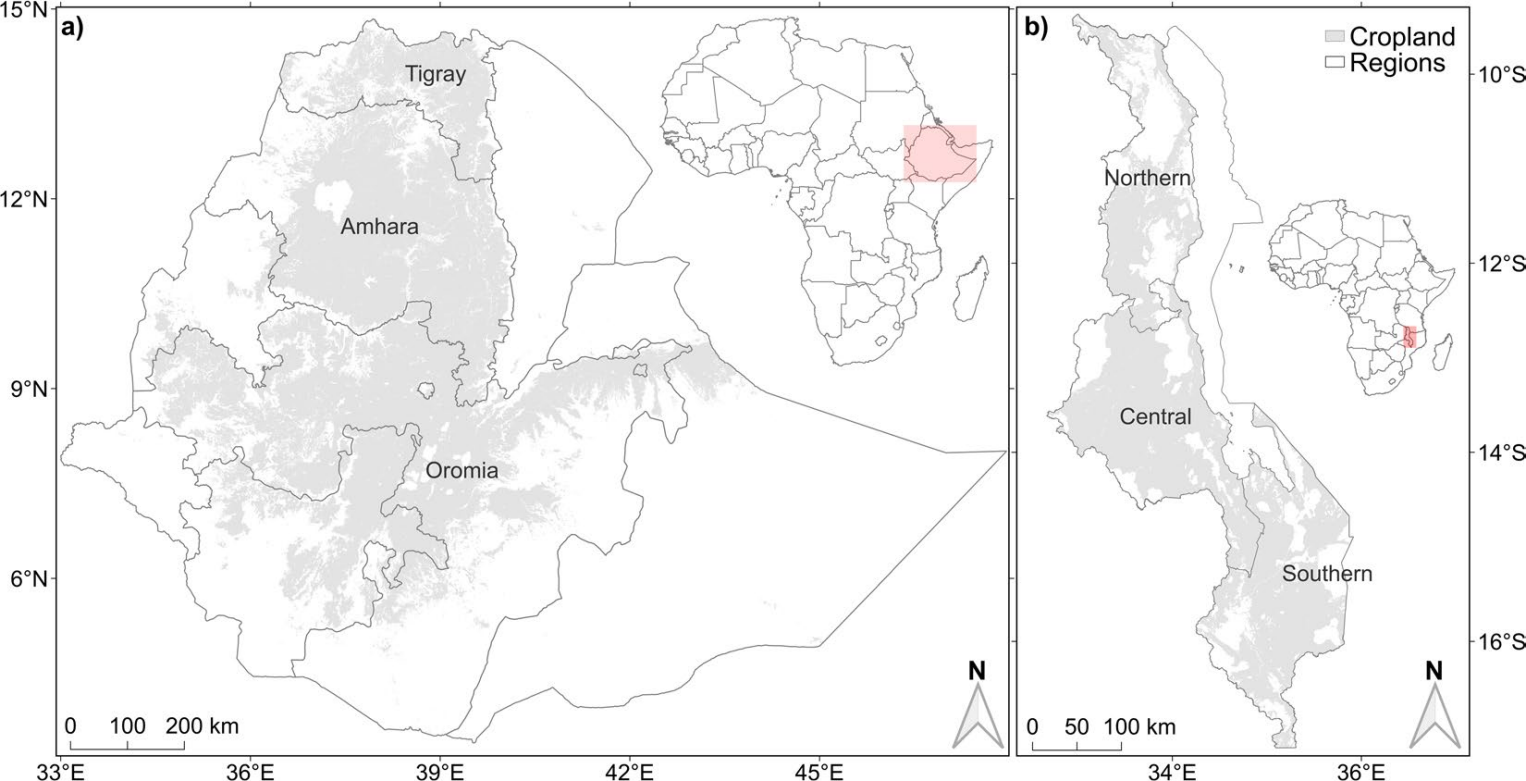
GeoNutrition cereal grain and soil sampling constrained to areas identified as croplands (shaded grey) in (**a**) the Amhara, Oromia and Tigray regions of Ethiopia, and (**b**) Malawi^7^. The (**a**) Ethiopian and (**b**) Malawian map insets in the African continental map are shaded pink.

Ethiopia’s Amhara, Oromia and Tigray regions were sampled (Fig. 1). Target sample frames in Ethiopia were constrained to locations on a 500-m grid (Lambert azimuthal equal-area projection) at which the probability of the land being under crop production had been mapped as ≥0.9^13^. The sample frame was further constrained to include only those locations on the 500-m grid that fell within 2.5 km of a road available in digital mapping format on OpenStreetMap (OSM)^14^. These constraints may introduce possible biases into predictions made at locations outside the designed sample frame, however, it would not otherwise have been possible to visit all the sample locations in the time available. Of a total land area of around 558,500 km^2^ in the three regions of Ethiopia, the total cropland mask represented 354,325 km^2^, of which 220,467 km^2^ was within 2.5 km of a OSM mapped road^13^. Because the sample frame was somewhat fragmented spatially the sample points were selected to achieve spatial balance and spread, the latter denoting spatial coverage^15^. A total of 1,825 sample sites were selected this way, and 175 of these were selected at random to be supplemented with a close pair site.

In Malawi, the cropland area was determined from the European Space Agency Climate Change Initiative^16^ land-use maps. The agricultural area used was defined as all raster cells that included the category of ‘cropland’ in their description (Fig. 1). In Malawi, where road access to cropped areas is generally better than in Ethiopia, no constraint to distance from a road was imposed on sample locations. A total of 820 sample sites were selected from the 2015/16 Demographic and Health Survey (DHS) of Malawi (REFS)^17^, all the DHS points in the sample frame. A further 890 sites were then selected to complete a spatial coverage survey, with the stratify function from the spcosa library in R^18^ which can draw a spatial coverage survey conditional on fixed points. An additional 190 close-pair sites were then added to a random subset of the spatial coverage points.

#### Field data collection

Field data collection was carried out using an open-source georeferenced survey data collection tool, KoBoToolBox (https://www.kobotoolbox.org/) and its companion mobile app KoBoCollect via questionnaires (see Supplementary file 3). Data recording, and cereal grain and soil sampling was conducted by teams of enumerators who were trained in standard operating procedures, and participatory risk assessments to safely navigate to target sampling sites. Sample teams used their discretion to exclude sample sites that may have jeopardised their safety (for example, via flooded roads), or created a disproportionate detour for single sample collection in mountainous regions. Field data collected include longitude, latitude, altitude, cereal crop species and source of cereal grain (i.e., standing crop, field stack or store).

#### Cereal grain and soil sampling

Sampling of cereal grain and soil (Table 1) from farmers’ fields in Ethiopia was completed in November 2017–February 2018 for most of the Amhara region and November 2018–February 2019 in all three regions (Fig. 1). Sampling in Malawi was completed in April–June 2018.

At each preselected target sample site, the team would identify the nearest field with a mature cereal crop within a 1-km radius, and take a grain and soil sample, subject to farmer consent. If a field with a standing mature cereal crop was not apparent, that is, the crop had been harvested, or a non-cereal crop had been grown, the team would ask the farmer to identify a field from which a cereal crop had recently been harvested and stored, and from which a sample could be obtained. If sampling was not possible, then the team would either look beyond a 1-km radius for an alternative site, or leave the site without taking samples.

Within a selected field, samples were taken from a 100 m^2^ (0.01 ha) circular plot. This was centred as close as practical to the middle of the field unless this area was unrepresentative due to disease or crop damage. Five subsample points were located (see Extended Data Fig. 1 in Gashu *et al*.^7^). The first point was at the centre of the plot. Two subsample points were then selected at locations on a line through the plot centre along the crop rows, and two more points on a line orthogonal to the first through the plot centre. Where possible, the central sampling location was fixed between crop rows, and the long axis of the sample array (with sample locations at 5.64 m and 4.89 m) was oriented in the direction of crop rows with the short axis perpendicular to the crop rows. A single soil subsample was collected at each of the five subsample points with a Dutch auger with a flight length of 0.15 m and diameter of 0.05 m. The auger was inserted vertically to the depth of one flight and the five subsamples were combined in a single Kraft^©^ paper bag. Where a mature or ripe crop was still standing in the field, grain samples were taken close to each augering position by a different operator, to minimise further contamination by dust and soil. For maize, a single cob was taken at each of the five points. Maize kernels were stripped from around 50% of each cob lengthways and composited into a single sample envelope for each location. For small-grained crops, sufficient stalks were taken so that approximately 20–50% of the sample envelope was filled (dimensions 0.15 m × 0.22 m), with samples placed grain‐first into the sample bag and the stalks were twisted off the grain heads and discarded. If a crop was in field stacks, then a subsample, comprising five cobs for maize, or a representative sample for other crops was taken from each available stack, taking material from inside the stack to minimize contamination by dust and soil (see Extended Data Fig. 1 in Gashu *et al*.^7^). If a crop was in a farmer’s store, it was considered an already-composited sample and a sample was taken while avoiding grain from the store floor if grain was loosely stored and avoiding grain with visible soil or dust contamination.

#### Sample management and preparation

Whole-grain samples were air-dried in their sample bags. Each sample was then ground in a domestic stainless-steel coffee grinder, which was wiped clean before use and after each sample with a non-abrasive cloth. All preparation was done away from sources of contamination by soil or by dust. A 20-g subsample of the ground material was then shipped to the University of Nottingham. Soil samples were oven-dried at 40 °C for 24–48 h depending on the moisture content of the soil. Preparation took place in a soil laboratory to avoid cross-contamination with grain samples. Plant material was removed from each soil sample, which was then disaggregated and sieved to pass 2 mm. This material was then coned and quartered to produce subsample splits. A 150-g subsample of soil was poured into a self-seal bag, labelled and shipped to the UK for analysis in the laboratories at Rothamsted Research and the University of Nottingham.

#### Chemical analysis methods

All grain and soil analyses were conducted in numerical sequence of the sample ID at Rothamsted Research and the University of Nottingham, UK.

### Grain analysis

**Fig. 2:**
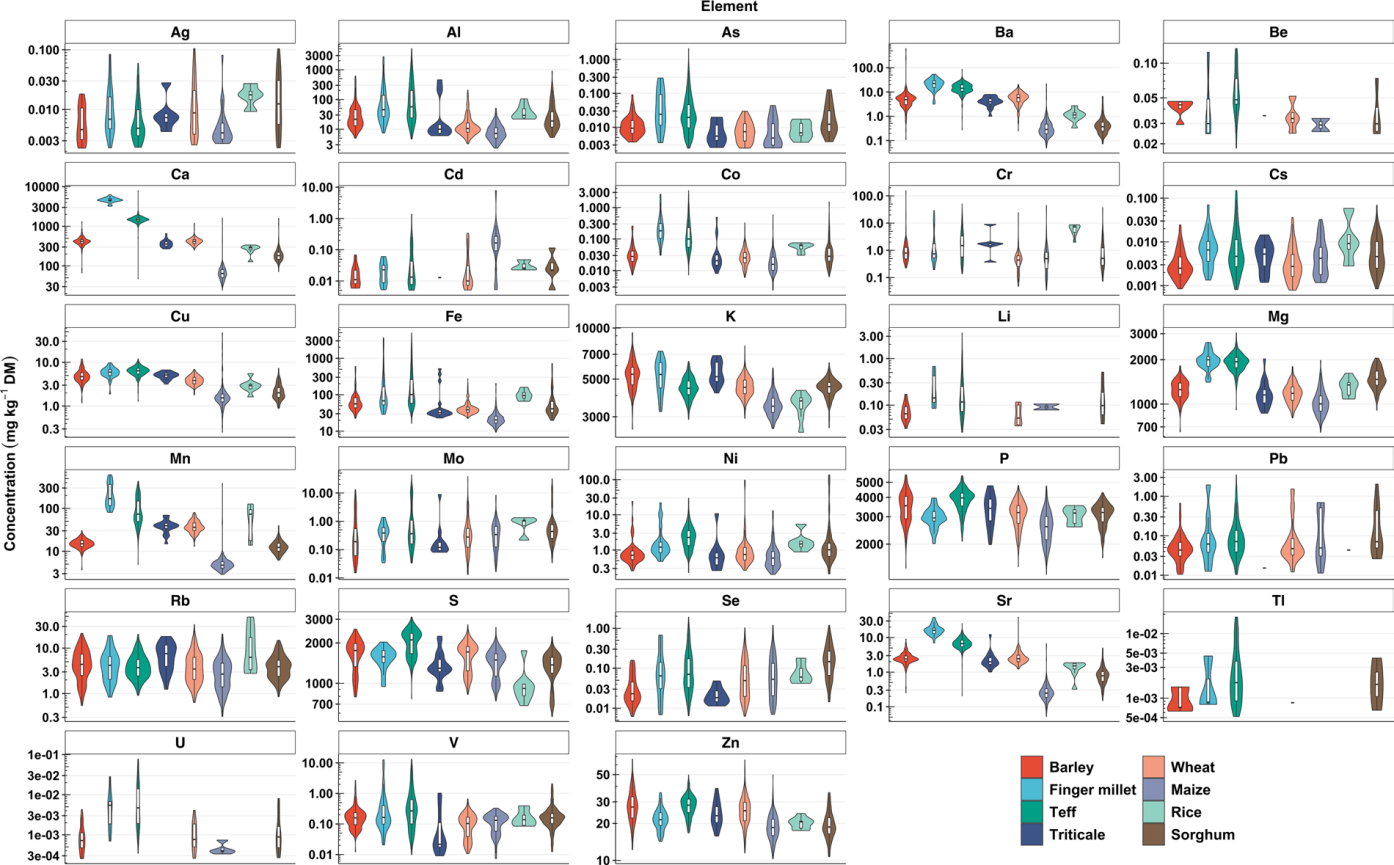
Combined violin and box-and-whisker plots of the elemental concentration in barley, finger millet, teff, triticale, wheat, maize, rice, and sorghum grains collected from Ethiopia. The middle line in the box represents the median, lower hinge Q1 and upper hinge Q3 of the quartiles, and the ends of the whiskers indicate the highest and lowest concentration values. The y-axis is shown as a logarithmic scale. See Table 1 for the number of samples for each crop which are for those samples greater than the LOD for each analyte. See Table 2 for the names of the elements.

**Fig. 3:**
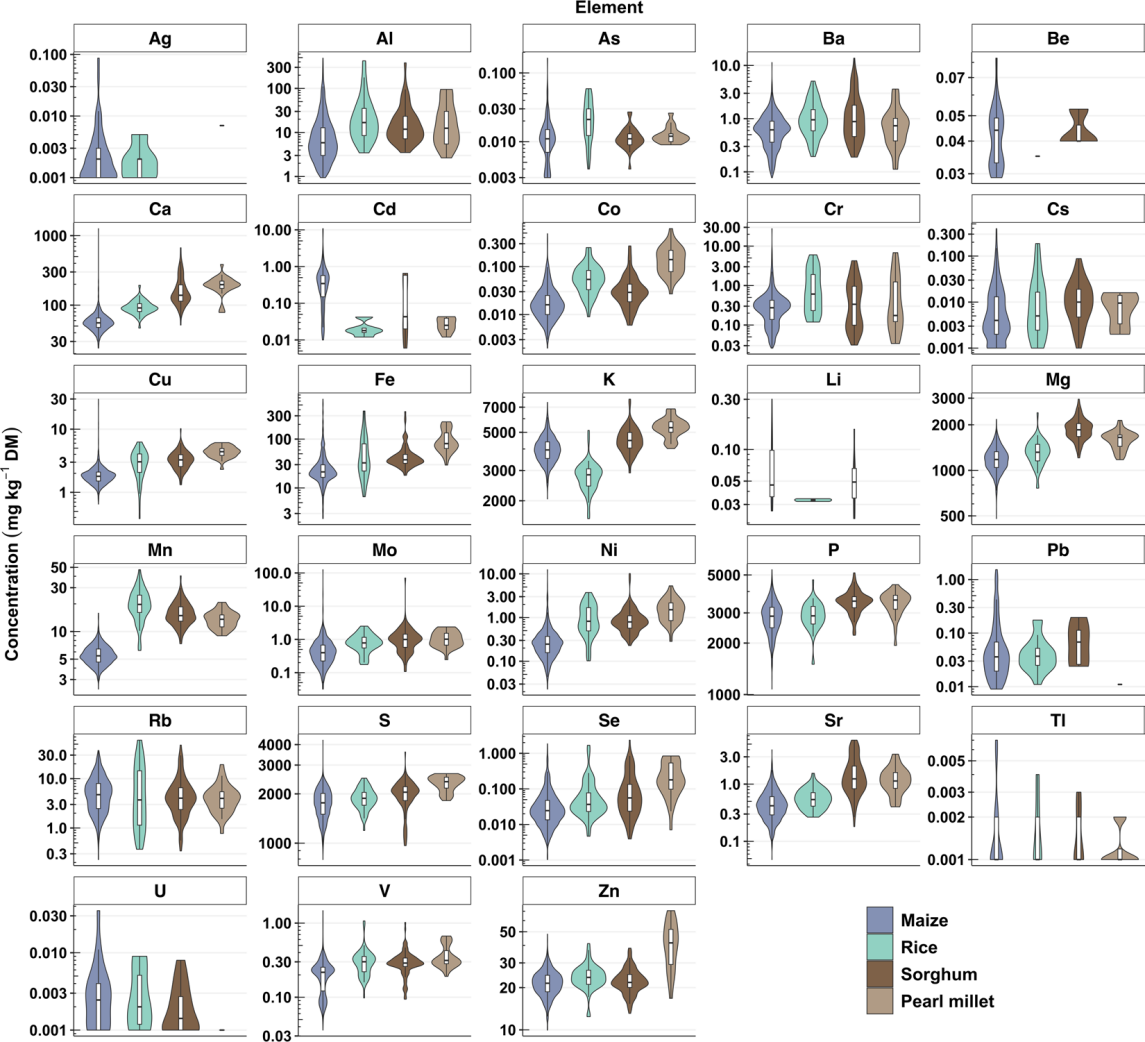
Combined violin, and box-and-whisker plots of the elemental concentration in maize, rice, sorghum and pearl millet grains collected from Malawi. The middle line in the box represents the median, lower hinge Q1 and upper hinge Q3 of the quartiles, and the ends of the whiskers indicate the highest and lowest concentration values. The y-axis is shown as a logarithmic scale. See Table 1 for the number of samples for each crop which are those samples greater than the LOD for each analyte. See Table 2 for the names of the elements.

Elemental concentrations (see Figs. 2 and 3 and Supplementary file 4) of grain were determined after microwave digestion of approximately 0.2 g of ground samples with concentrated nitric acid (70% HNO_3_, trace analysis grade). Samples collected from the Amhara Region of Ethiopia in 2017 were microwave digested using a Multiwave 3000 48-vessel MF50 rotor (Anton Paar GmbH, Graz, Austria) in 2 mL HNO_3_, 1 mL Milli-Q water (18.2 MΩ cm; Fisher Scientific) and 1 mL H_2_O_2_ at a power of 1400 W, temperature 140 °C, pressure of 2 MPa for 45 min. Samples collected in Malawi and Ethiopia in 2018–2019 were microwave digested in a Multiwave Pro with a 41HVT56 rotor and pressure-activated venting vessels made of modified polytetrafluoroethylene (56-ml ‘SMART VENT’, Anton Paar). The digestion was achieved using 6 mL of HNO_3_. at a power of 1,500 W, with 10 min heating to 140 °C, 20 min holding at 140 °C, and 15 min cooling to 55 °C. After digestion, the samples were made to 15 mL using Milli-Q water, then stored in capped tube at room temperature for ≈ 1 week until the chemical analysis. Prior to analysis by inductively coupled plasma mass spectrometry (ICP-MS; Thermo Fisher Scientific iCAP Q, Thermo Fisher Scientific, Bremen, Germany), samples were further diluted 1:5 with Milli-Q water.

Due to low concentrations of Se in many of the grain samples taken in Malawi, a substantial number of values were below the limit of detection (LOD) when measured at a mass to charge ratio of 78 (m/z = 78) on ICP-MS. Consequently, samples collected in Malawi and Ethiopia were re-analysed for Se using QQQ-ICP-MS (iCAP TQ; Thermo Fisher Scientific, Bremen, Germany) with oxygen mass shifting of the Se peak at m/z = 80 to m/z 96.

### Soil chemical analysis

#### Soil characterisation

Unless otherwise specified, all analysis were performed on samples sieved to < 2 mm. Soil pH was measured in de-ionised water (pH_w) suspension (1:2.5 solid to solution ratio) using a Jenway 3540 meter (Cole-Parmer, Stone, Staffordshire, UK), with a temperature-compensated combination pH electrode. Soil pH was also measured in the 0.01 M Ca(NO_3_)_2_ (pH_CaNO) suspension using a Mettler-Toledo AG pH meter (Mettler-Toledo, Beaumont Leys, Leicester, UK) also with a temperature compensating electrode. Total C and N were determined by dry combustion^19^ using a Leco TruMac CN Combustion analyzer (LECO Corporation, St. Joseph, Michigan, USA). Inorganic C was determined by Skalar Primacs Inorganic Carbon Analyser (Skalar Analytical BV, Breda, Netherlands). Estimates of amorphous oxides and poorly crystalline oxides (E_Oxa_) were determined following ammonium oxalate extraction^20^. Soil effective cation exchange capacity (eCEC) and exchangeable cations (Na_Exch_, Mg_Exch,_ K_Exch,_ and Ca_Exch_) were determined using one-step extraction^21^ with cobalt(III) hexamine chloride solution and analysis by inductively coupled plasma optical emission spectrometry (ICP–OES; Perkin Elmer Life and Analytical, Shelton, USA). Available phosphorus (P_Olsen_) was determined after extraction with sodium bicarbonate as described by Olsen^22^. Phosphate buffering index (PBI) was also determined, as an indicator of the soil’s ability to control changes in P concentration in the soil solution^23^.

#### Total elemental concentration

Quasi-total concentrations of major and trace elements (E_Tot_) in the soils were determined after aqua regia extraction^24^ of finely ground samples using ICP-OES (ICP-OES; PerkinElmer Life and Analytical, Shelton, Connecticut, USA) and ICP-MS.

#### Extractable elemental concentration

Elements extractable with DTPA (E_DTPA;_ potentially phytoavailable) were determined by shaking c. 5 g soil with 10 mL of 0.005 M DTPA, 0.1 M triethanolamine (TEA) and 0.01 M CaCl_2_ at pH = 7.3 for 2 h on an end-over-end shaker^25^, followed by centrifugation (3500 rpm), filtration (0.22 µm) and analysis by ICP-MS (iCAP Q; Thermo Fisher Scientific, Bremen, Germany). Soluble major and trace elements (E_Sol_Ca_; readily available) were determined in the solution phase of soil suspensions in 0.01 M Ca(NO_3_)_2_ (1:10 soil: solution ratio) following equilibration for 4 days on an end-over-end shaker. The solutions were isolated by centrifugation and filtration (0.22 μm) prior to elemental analysis by ICP-MS (iCAP Q). For soil samples collected from the Amhara Region of Ethiopia in 2017, soluble major and trace elements (E_CaCl_2_) were also determined in the solution phase of soil suspension in 0.01 M CaCl_2_ (1:10 soil:solution ratio) and equilibration for 2 hours on end-over-end shaker followed by centrifuge and filtration prior to analysis by (ICP–OES; Perkin Elmer Life and Analytical, Shelton, USA). Non-purgeable organic carbon (NPOC) was also determined in 0.01 M CaCl_2_ extraction using Chemical UV Oxidation Total Organic Carbon Analyser (Shimadzu Corporation, Japan). Concentrations of the free ion activity of Zn were predicted for these samples (Amhara Region of Ethiopia) using the Windermere Humic Aqueous (WHAM) geochemical model as described in detail in Mossa *et al*.^11^.

#### A three-step sequential extraction scheme of sulfur and selenium in soil

This fractionation scheme was adapted from the one used by Mathers *et al*.^26^ for soil Se and Shetaya *et al*.^27^ for soil iodine and is intended as a general scheme for extraction of oxy-acid anions. The method aims to sequentially extract (i) a ‘soluble’ fraction in 0.01 M KNO_3_, (ii) a ‘specifically adsorbed’ fraction in 0.016 M KH_2_PO_4_, and (iii) an organically bound fraction in 10% tetra methyl ammonium hydroxide (TMAH). It is important to note that none of the three fractions are likely to contain the analyte as a single species. For example, the ‘soluble’ Se fraction will typically include selenate, selenite and dissolved organic forms of Se. A mass of dried soil equivalent to ≈4.0 g, sieved to <2 mm, was weighed into a polyethylene centrifuge tube. After adding 20 mL of 0.01 M KNO_3_, the tubes were shaken for 2 h on an end-over-end shaker, then centrifuged at 3500 rpm for 30 min. A volume of 9 mL of supernatant was filtered (0.22 µm) using PTFE syringe filters, into tubes containing 1 mL of a mixture of 0.1 M KH_2_PO_4_ and 10% TMAH to preserve the samples before analysis. After removing the excess supernatant, the centrifuge tubes with wet soil pellets were weighed to account for carry over of 0.01 M KNO_3_ extract then 20 mL of 0.016 M KH_2_PO_4_ was added. The tubes were vortexed to disaggregate the soil pellet and then shaken for 1 h before centrifugation at 3500 rpm for 30 min. A volume of 9 mL of the supernatant was filtered to (0.22 µm), into a tube containing 1 mL of 10% TMAH. After removing excess supernatant, the tubes were weighed again before 10 mL of 10% TMAH was added. The tubes were vortexed to disaggregate the pellet, loosely capped and incubated at 70 °C for ∼16 h before centrifugation (3500 rpm for 30 min). Extracts (1 mL) were then diluted with 9 mL of ultrapure MQ water to give a final solution of 1% TMAH. Samples were analysed for S and Se using a QQQ-ICP-MS operated in oxygen cell mode with rhenium (^187^Re; 20 µg L^−1^) and indium (^115^In; 10 µg L^−1^) as internal standards to correct for instrumental drift. Sulfur and Se were measured in mass-shift mode following reaction with oxygen to form the analyte ions SO^+^ (m/z 32 → 48), and SeO^+^ (m/z 80 → 96).

#### Zinc isotopic dilution assay

**Fig. 4:**
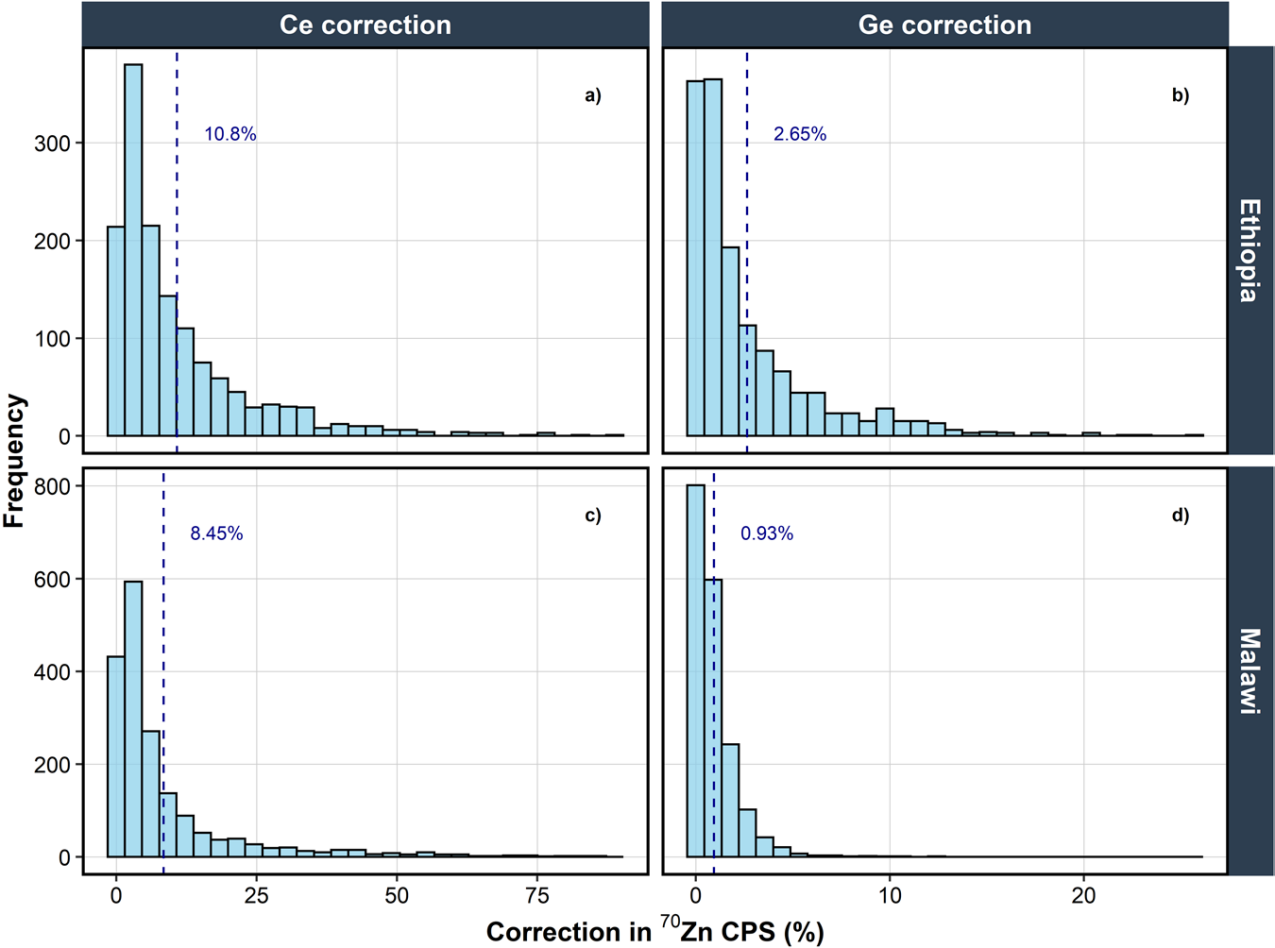
Histograms showing the percentage correction in ^70^Zn counts per second (CPS) resulting from cerium (Ce) interferences in samples from (**a**) Ethiopia and (**c**) Malawi, and percentage correction resulting from germanium (Ge) interferences in samples from (**b**) Ethiopia and (**d**) Malawi. Vertical blue dashed lines represent mean values.

Isotopically exchangeable Zn was determined using the method described in detail in Mossa *et al*.^11^. Briefly, a mass of 2 g sieved, air–dried soil was equilibrated with 20 mL of 0.01 M Ca(NO_3_)_2_ for 24 h. The soil suspension was then spiked with a ^70^Zn isotopic tracer and further equilibrated for 72 h. To avoid acidification, the pH of the spike solution was adjusted to pH 4.0–4.5 using an ammonium acetate buffer immediately before use. Samples were centrifuged (3500 rpm for 15 minutes), filtered (0.22 µm) and the supernatant acidified to 2% HNO_3_. Isotopic analysis was carried out using ICP-MS (iCAP Q) operating in collision cell mode using He for kinetic energy discrimination (KED). Significant and variable interferences (soil derived ^70^Ge^+^ and (plasma generated) doubly charged ^140^Ce^++^) on ^70^Zn required correction and was achieved by analysing Ge and Ce standards alongside the samples and inferring the intensity (count per second, CPS) from the measured CPS ratio 72/70 for Ge standards and 70/140 for Ce standards. The interference from Ge produced a correction for ^70^Zn CPS ranging from 0.01–25% (median = 0.74%; mean = 1.68%), while the correction resulted from Ce interference ranged from 0.03–88% (median = 4.63%; mean = 9.48%) (Fig. 4).

## Data Records

The cereal grain elemental concentration and soil chemistry properties dataset is provided as a set of OpenDocument workbooks and zipped folders containing comma separated value (CSV) files of the worksheets for Ethiopia and for Malawi. The Ethiopian workbook contain six worksheets: ETH_CropSoilData_Raw, ETH_CropSoilData_NA, ETH_Crop_LOD_ByICPRun, CropElements, SoilProperties and Notes. The worksheets in the Malawian workbook are MWI_CropSoilData_Raw, MWI_CropSoilData_NA, Crop_LOD_ByICPRun, CropElements, SoilProperties, and Notes. The individual CSV filename is the same as the worksheet name in the workbooks. The ‘Notes’ worksheets in both Ethiopian and Malawian workbooks and CSV zipped folders provide greater detail about the data structure and contents of each worksheet and fields. It also provides details of how data from different worksheets may be linked using the various identifier fields (e.g., how the LOD for a given cereal crop elemental concentration data record can be linked using the Crop_ICP_Run field). The dataset is accessible currently from the figshare data repository^28^ at 10.6084/m9.figshare.15911973. Figshare uses MD5 checksums when storing a file, which are checked against the file regularly to ensure the file is intact and to verify the integrity of downloads. See Usage Notes before data re-use.

### Grain and soil chemistry data

Workbook and spreadsheet names containing these data begin with the three-letter ISO country code for Ethiopia (ETH) and Malawi (MWI), as well as details about what is being stored. The workbook and CSV folders containing the cereal grain and soil properties data for Ethiopia is named **ETH_CropSoilChemData** and for Malawi **MWI_CropSoilChemData**. Due to the complexity in data recording and reporting, we have reported the data in two ways^28^. In each workbook, the first worksheet (**Country_CropSoilData_Raw**) contains raw data recorded from the analytical equipment and software used to process the data. To avoid blank cells, “NM” (not measured) is used here to indicate where data were not measured. In the second worksheet (**Country_CropSoilData_NA**) data below the LOD, including negative data values, and missing data are replaced by NA (not available).

### Ethiopian data

The first 12 fields in the raw and cleaned Ethiopian cereal grain and soil chemistry data worksheets are auxiliary and field data for the cereal grain-soil sample pairs (records). These are described as follows:

1. **FundingSource**: The source of funding to carry out the research that generated these data. **BMGF** = Bill & Melinda Gates Foundation (INV-009129); **GCRF** = Global Challenges Research Fund (BB/P023126/1).
2. **ID**: Data record (row) identification number (ID) for the dataset. These are unique IDs that can be used as a primary key to conduct separate analyses on cereal grain and soil chemistry properties data.
3. **Crop_ICP_Run**: The ICP-MS (Inductively Coupled Plasma Mass Spectrometer) run number to determine the crop elemental LOD for the raw cereal grain dataset using the LOD data in the ***ETH_Crop_LOD_ByICPRun*** worksheet.
4. **Latitude**: In decimal degrees, WGS84 datum, coordinate reference system EPSG:4326.
5. **Longitude**: In decimal degrees, WGS84 datum, coordinate reference system EPSG:4326.
6. **Altitude**: The altitude in meters above sea level.
7. **LocationPrecision**: The horizontal and vertical positional accuracy, in meters.
8. **SamplingStart**: Start of sampling date and time stamp, UTC + 3. This is the date and time automatically recorded by the KoBoCollect app when the enumerators start recording cereal grain and soil sample information in the field. The start sampling date for the ID ETH1219 was incorrect and removed.
9. **SamplingEnd**: End of sampling date and time stamp, UTC + 3. This is the date and time automatically recorded by the KoBoCollect app when the enumerators either save or submit the sample metadata recording questionnaire.
10. **Crop:** Type of crop from which cereal grains were sampled.
11. **GrainSource**: Source of cereal grain (i.e., standing crop, field stack or store).
12. **Site**: Whether the site is “main” or “close pair”. Please see Gashu *et al*.^7^ for further details on this.

### Malawian data

Identical to the Ethiopian dataset, the first 12 fields in the raw and cleaned Malawian cereal grain and soil chemistry data worksheets are auxiliary and field data for the cereal grain-soil sample pairs (records). The data details which vary from above are as follows:

- **Crop_ICP_Run**: The ICP-MS run number to determine the crop elemental LOD for the raw cereal grain dataset except Se using the data in the **Crop_LOD_ByICPRun** worksheet.
- **Crop_ICP_Run_Se**: The ICP-MS run number to determine the cereal grain Se concentration LOD for the raw cereal grain dataset using the LOD data in the **Crop_LOD_ByICPRun** worksheet.

#### Cereal grain elemental concentration

In both Ethiopia and Malawi datasets, the cereal grain elemental concentration for 29 elements (columns or data fields) was reported (Table 2). These appear in alphabetical order of element symbol next to the field and auxiliary data described in the sections above. The data field names also contain **_grain** suffix after the elemental symbol.

#### Soil chemistry

In the Ethiopian and Malawian datasets, 84 and 69 soil chemical properties, respectively were reported (Online-only Table 1). Please refer to the individual country dataset to find out which soil chemical properties were analysed and reported. These are presented in alphabetical order after the cereal grain elemental concentration data columns.

## Technical Validation

### Grain analysis

Quality control protocols for grain analysis included two operational blanks in each digestion batch and duplicate samples of a certified reference material (CRM) (Wheat flour SRM 1567b, National Institute of Standards and Technology, Gaithersburg, MD, USA) in approximately every fourth digestion batch. An LOD was calculated as 3 times the standard deviation of 10–14 blanks assuming a hypothetical mass of 0.2 g of sample. The data for percentage recoveries of CRM is shown in Table 3.

**Table 3:** The percentage recovery of elements of the certified material (Wheat flour SRM 1567b) used in microwave acid digestion of grain samples.

Country	ICP_Run	Recovery (%) by element															
		Al	As	Ca	Cd	Cu	Fe	K	Mg	Mn	Mo	P	Pb	Rb	Se	V	Zn
**ETH_GCRF**	**1**	75.4	78.7	96.3	97.5	87.2	92.3	102.0	95.5	96.2	82.8	96.2	78.9	97.1	98.0	88.7	91.1
	**2**	80.9	72.7	97.5	84.2	90.4	89.6	100.5	95.1	96.0	100.1	95.9	62.2	94.0	95.0	89.4	89.9
**ETH_BMGF**	**1**	37.7	105.1	102.7	233.1	94.8	124.0	105.9	99.3	98.8	96.1	97.0	119.2	96.2	100.0	−10.8	79.3
	**2**	71.1	54.3	101.9	62.6	92.5	89.7	100.0	93.2	96.0	94.5	98.2	78.2	94.3	97.7	320.9	91.7
	**3**	71.0	81.1	99.2	123.5	96.5	90.2	100.3	95.3	96.2	669.0	95.2	124.6	96.1	99.6	426.1	91.5
	**4**	69.6	53.3	105.4	96.6	97.7	98.7	104.8	97.6	99.4	98.4	102.6	42.7	99.9	101.2	190.8	95.2
	**5**	77.6	72.1	98.9	117.2	86.9	80.0	98.8	92.3	93.2	93.0	95.3	−162.4	93.8	94.3	321.0	85.9
**MWI**	**1**	77.7	81.7	106.2	94.1	97.9	118.5	109.1	101.2	99.6	83.5	96.4	1176.9	98.9	101.6	184.8	102.4
	**2**	77.4	70.0	103.0	94.2	92.9	90.9	107.1	99.5	95.3	106.0	97.5	124.8	95.2	98.3	112.8	92.8
	**3**	78.2	57.6	104.5	96.2	97.9	200.9	110.8	104.9	99.9	94.6	97.4	119.5	98.6	102.8	102.8	92.2
	**4**	96.5	615.8	105.4	74.9	96.3	88.7	118.0	109.1	98.9	96.3	102.6	136.0	100.0	101.5	428.8	89.7
	**5**	85.6	205.3	106.6	−51.0	100.6	94.6	121.7	115.0	103.6	116.4	102.2	−11.5	103.3	103.6	87.2	101.2
	**6**	80.6	115.1	100.5	123.2	91.3	90.2	113.5	108.2	95.8	92.8	95.5	−101.6	96.7	95.6	178.8	86.7
	**7**	76.6	215.0	99.6	93.4	98.3	89.9	115.6	109.4	98.2	114.9	98.2	165.3	99.3	99.2	234.8	91.8
	**8**	66.1	132.4	99.0	97.4	95.2	106.7	116.4	110.4	99.7	99.5	99.7	121.0	98.4	99.2	86.7	88.5

The cereal grains collected from Ethiopia (ETH_GCRF and ETH_BMGF) and Malawi (MWI). ICP Run is the inductively coupled plasma mass spectrometer (ICP-MS) run number.

### Soil chemical analysis

Quality control protocols included two operational blanks in each digestion batch and two samples of an internal or external reference material. Ten percent of samples were repeated through both the extraction/digestion and analysis stages, and the results were expected to fall within ± 5%, if not, the sample batch was repeated apart from results near to the LOD (which occurs with some samples that have low trace element concentrations). All instruments used were drift-corrected during each run as part of the calibration procedure. In each batch of soil pH_w determination, 10% unknowns were repeated and duplicate samples of in-house standard Broadbalk 082 (Rothamsted Research) were included for quality control, and we participated in the Wageningen Evaluating Programmes for Analytical Laboratories (WEPAL) inter-laboratory proficiency testing for soil pH. Total C and N analysis was set up with LECO Soil standard LCRM 502–697, lot 1000, and validated over time using proficiency WEPAL testing. No CRM was available for inorganic C, so in-house standards Summerdells, Broadbalk, Sacrewell (Rothamsted Research) were used in each batch, as well as WEPAL proficiency testing. No CRM was available for E_Oxa_, and in-house standards Leuven and Woburn (Rothamsted Research) were used. No CRM was available for eCEC, and in-house standard Leuven soil (Rothamsted Research) was used. No CRM was available for P_Olsen_, and in-house Hoosfield Plot 714 and Plot 444 (Rothamsted Research) were used, as well as WEPAL proficiency testing. No CRM was available for PBI, and in-house standards Leuven and Woburn were used.

For quasi-total concentrations, 10% blanks (extraction solution) were included to check for potential contamination of analytes of interests. Batches were rejected if the blanks showed apparent signals greater than 3x SD of the background signal of the instrument (limit of detection). WEPAL soil reference material ISE 962 were used to check analytical precision of every batch (Online-only Table 2), as well as WEPAL proficiency testing. Comparing average results after blank subtraction for all elements with the results for ISE 962 from WEPAL shows recoveries of +/−10% for most of the 20 elements. Exceptions are As, Cd, Na and Ti by ICP-OES, and Cd, Mo and Se by ICP-MS (Online-only Table 3). Note that As, Cd and Pb were determined by ICP-OES only for the GCRF samples from Ethiopia (11 batches) but for all of the other samples in the BMGF project these elements were determined by ICP-MS. Analysis by ICP-MS compared to ICP-OES improved both the recovery and variation for As; for Cd the mean value decreased and so did the variation; Pb was similar but with decreased variation. Na recovery by ICP-OES and aqua regia extraction can be incomplete, and the results WEPAL given for Ti are only indicative values. Limits of detection for each of the above methods of soil analysis, except pH, are shown in Online-only Tables 4, 6, 8, 10, 12 to enable users to decide whether results for individual samples are reliable. Results for blanks are also shown in online-only Tables 5, 7, 9, 11, 13 and, except for quasi-total elements, these have not been subtracted from the results in the soil data. However, this information has been provided to enable users to judge the magnitude of the blanks compared to sample results for each method, and to enable them to decide whether they subtract them.

**Fig. 5:**
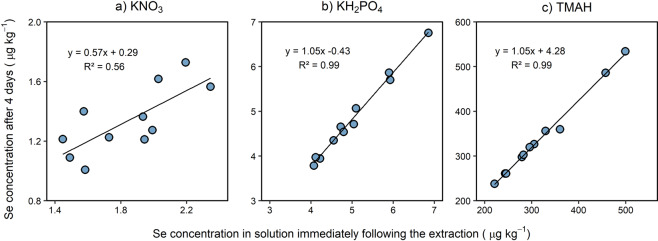
Selenium concentrations in soil measured immediately following (**a**) potassium nitrate (KNO_3_) extraction; (**b**) potassium phosphate (KH_2_PO_4_) extraction; (**c**) Tetramethylammonium hydroxide (TMAH) extraction and measured again after 4 days of storage of soils from Amhara region, Ethiopia.

For the three-step sequential extraction scheme of S and Se in soil, a preliminary experiment was conducted to test the temporal stability of the analytes in a 1% TMAH matrix; analytes were measured after the extraction was completed and then again after storage for 4 days. The results showed that the agreement between the two measurements was quite poor for the KNO_3_ extraction, especially in case of Se (Fig. 5), whereas there were very good agreements between the two measurements in the case of the KH_2_PO_4_ and TMAH extractions. Consequently, concentrations in the soluble fractions (0.01 M KNO_3_) were preserved in a mixture of 0.01 M KH_2_PO_4_ and 1% TMAH.

**Fig. 6:**
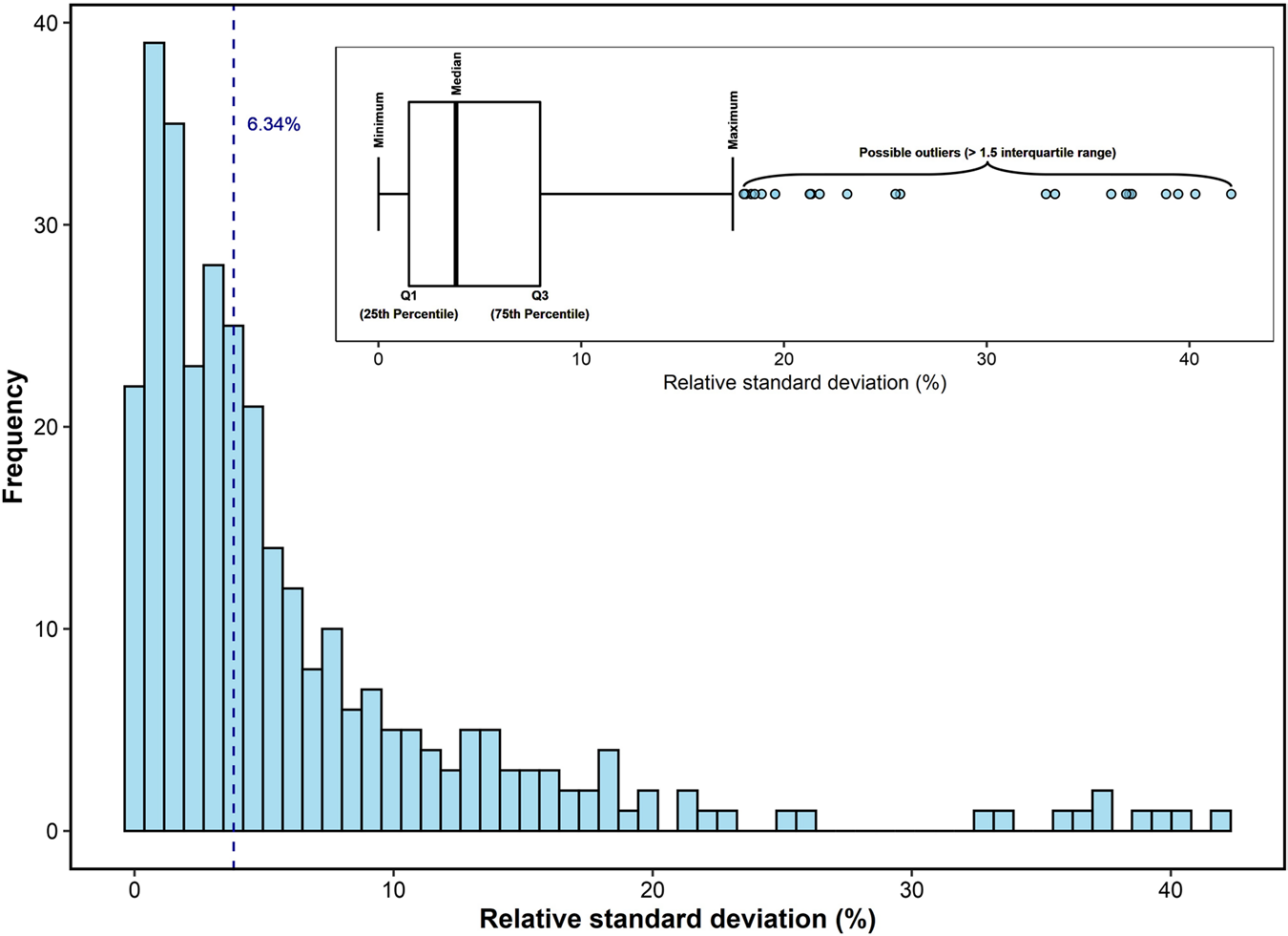
Frequency of the relative standard deviation (RSD) of duplicate E-value measurements on a subset of soils from Ethiopia and Malawi. Vertical dashed blue line represents the mean value of RSD. The inserted graph shows a whisker-boxplot of the distribution of the RSD of duplicate measurements.

The reproducibility of the isotopic dilution analysis was tested by repeating c. 10% of the samples with precision determined by calculating the relative standard deviation (RSD; %) of the duplicates. The average (error) RSD was 6.34% (sd = 7.68%), and 80% of the repeated samples had values of RSD < 10% (Fig. 6).

Auxiliary information on the soil samples for the various soil analytical approaches are presented in Supplementary files 5 and 6.

## Usage Notes

- Cereal grain elemental concentration data presented in Supplementary File 4 were derived from data which excluded concentration values ≤ LOD, including negative values. For some elements, where there are larger numbers of cereal grain samples with concentrations ≤ LOD, this can lead to over-estimation of the median concentration values compared with when all data are used. For example, if all data, including those ≤ LOD, are used, the median Se concentration in maize in Malawi is 0.0168 mg kg^−1^ (n = 1,603). When data values ≤ LOD are excluded, the median Se concentration in maize is 0.02448 mg kg^−1^ (n = 1199). Users wanting to use descriptive data from summary tables should be aware of this, and select data in formats which are appropriate to their purpose. This note also applies to some of the soil chemistry summary table where there are many negative concentrations in the data.
- Users should be aware that some soil properties were not measured in both the GCRF-funded and BMGF-funded rows in the Ethiopian dataset. The following soil chemistry data were not measured in the GCRF-funded records: Cd_DTPA, Co_DTPA, Cu_DTPA, Fe_DTPA, Mn_DTPA, Ni_DTPA and Pb_DTPA. Similarly, the following soil chemistry properties were not measured in BMGF- funded records: Co_CaCl2, Cu_CaCl, Fe_CaCl, K_CaCl, Mg_CaCl, Mn_CaCl, Mo_CaCl, Na_CaCl, Ni_CaCl, NOPC, P_CaCl, Se_CaCl, Zn_CaCl and Zn_FIA.
- Users wanting to carry out any geospatial analyses using the latitude and longitude fields in these data should ensure that the spatial (horizontal and vertical) accuracy reported meets the requirements of their analyses.

## Supplementary information

Supplementary file 3

Supplementary file 4

Supplementary file 5

Supplementary file 6

ETH_CropSoilData_Raw

ETH_CropSoilData_NA

ETH_Crop_LOD_ByICPRun

ETH_CropElements

ETH_SoilProperties

ETH_Notes

MWI_CropSoilData_Raw

MWI_CropSoilData_NA

MWI_Crop_LOD_ByICPRun

Supplementary file 1

Supplementary file 2

MWI_CropElements

MWI_Notes

## Data Availability

There is no specific code developed to access these data. Users can use and process the data in software of their choice.

## Online-only Tables

**Online-only Table 1:** List of data field name, analyte, chemical property, extraction methods and units of measurement reported in the Ethiopian and Malawian dataset.

Field name	Analyte	Property	Extraction	Unit
**Al_Oxa**	Al	Amorphous oxide	Oxalate	mg kg^−1^ DM
**Al_Sol_Ca**	Al	Soluble	0.01 M Ca(NO_3_)_2_	mg kg^−1^ DM
**Al_Tot_Aqu**	Al	Total	aqua regia	mg kg^−1^ DM
**As_Tot_Aqu**	As	Total	Aqua regia	mg kg^−1^ DM
**Ba_Sol_Ca**	Ba	Soluble	0.01 M Ca(NO_3_)_2_	mg kg^−1^ DM
**C_inorg**	C	Inorganic C	H_3_PO_4_	%
**C_org**	C	Organic C	Calculated (C_tot – C_inorg)	%
**C_tot**	C	Total C	Combustion	%
**Ca_CEC**	Ca	Exchangeable	Cobalt (III) hexamine	cmolc kg^−1^
**Ca_Tot_Aqu**	Ca	Total	Aqua regia	mg kg^−1^ DM
**Cd_DTPA**	Cd	Potentially phytoavailable	DTPA	mg kg^−1^ DM
**Cd_Sol_Ca**	Cd	Soluble	0.01 M Ca(NO_3_)_2_	mg kg^−1^ DM
**Cd_Tot_Aqu**	Cd	Total	Aqua regia	mg kg^−1^ DM
**Co_CaCl**	Co	Soluble	0.01 M CaCl_2_	mg kg^−1^ DM
**Co_DTPA**	Co	Potentially phytoavailable	DTPA	mg kg^−1^ DM
**Co_Sol_Ca**	Co	Soluble	0.01 M Ca(NO_3_)_2_	mg kg^−1^ DM
**Co_Tot_Aqu**	Co	Total	Aqua regia	mg kg^−1^ DM
**Cr_Tot_Aqu**	Cr	Total	Aqua regia	mg kg^−1^ DM
**Cu_CaCl**	Cu	Soluble	0.01 M CaCl_2_	mg kg^−1^ DM
**Cu_DTPA**	Cu	Potentially phytoavailable	DTPA	mg kg^−1^ DM
**Cu_Sol_Ca**	Cu	Soluble	0.01 M Ca(NO_3_)_2_	mg kg^−1^ DM
**Cu_Tot_Aqu**	Cu	Total	Aqua regia	mg kg^−1^ DM
**eCEC**	eCEC	Effective cation exchange capacity	Cobalt (III) hexamine	cmolc kg^−1^
**Fe_CaCl**	Fe	Soluble	0.01 M CaCl_2_	mg kg^−1^ DM
**Fe_DTPA**	Fe	Potentially phytoavailable	DTPA	mg kg^−1^ DM
**Fe_Oxa**	Fe	Amorphous oxide	Oxalate	mg kg^−1^ DM
**Fe_Sol_Ca**	Fe	Soluble	0.01 M Ca(NO_3_)_2_	mg kg^−1^ DM
**Fe_Tot_Aqu**	Fe	Total	Aqua regia	mg kg^−1^ DM
**K_CaCl**	K	Soluble	0.01 M CaCl_2_	mg kg^−1^ DM
**K_CEC**	K	Exchangeable	Cobalt (III) hexamine	cmolc kg^−1^
**K_Sol_Ca**	K	Soluble	0.01 M Ca(NO_3_)_2_	mg kg^−1^ DM
**K_Tot_Aqu**	K	Total	Aqua regia	mg kg^−1^ DM
**Mg_CaCl**	Mg	Soluble	0.01 M CaCl_2_	mg kg^−1^ DM
**Mg_CEC**	Mg	Exchangeable	Cobalt (III) hexamine	cmolc kg^−1^
**Mg_Sol_Ca**	Mg	Soluble	0.01 M Ca(NO_3_)_2_	mg kg^−1^ DM
**Mg_Tot_Aqu**	Mg	Total	Aqua regia	mg kg^−1^ DM
**Mn_CaCl**	Mn	Soluble	0.01 M CaCl_2_	mg kg^−1^ DM
**Mn_DTPA**	Mn	Potentially phytoavailable	DTPA	mg kg^−1^ DM
**Mn_Oxa**	Mn	Amorphous oxide	Oxalate	mg kg^−1^ DM
**Mn_Sol_Ca**	Mn	Soluble	0.01 M Ca(NO_3_)_2_	mg kg^−1^ DM
**Mn_Tot_Aqu**	Mn	Total	Aqua regia	mg kg^−1^ DM
**Mo_CaCl**	Mo	Soluble	0.01 M CaCl_2_	mg kg^−1^ DM
**Mo_Sol_Ca**	Mo	Soluble	0.01 M Ca(NO_3_)_2_	mg kg^−1^ DM
**Mo_Tot_Aqu**	Mo	Total	Aqua regia	mg kg^−1^ DM
**N_tot**	N	Total N	Combustion	%
**Na_CaCl**	Na	Soluble	0.01 M CaCl_2_	mg kg^−1^ DM
**Na_CEC**	Na	Exchangeable	Cobalt (III) hexamine	cmolc kg^−1^
**Na_Sol_Ca**	Na	Soluble	0.01 M Ca(NO_3_)_2_	mg kg^−1^ DM
**Na_Tot_Aqu**	Na	Total	Aqua regia	mg kg^−1^ DM
**Ni_CaCl**	Ni	Soluble	0.01 M CaCl_2_	mg kg^−1^ DM
**Ni_DTPA**	Ni	Potentially phytoavailable	DTPA	mg kg^−1^ DM
**Ni_Sol_Ca**	Ni	Soluble	0.01 M Ca(NO_3_)_2_	mg kg^−1^ DM
**Ni_Tot_Aqu**	Ni	Total	Aqua regia	mg kg^−1^ DM
**NOPC**	non-purgeable organic carbon	Dissolved organic C		mg L^−1^
**P_CaCl**	P_	Soluble	0.01 M CaCl_2_	mg kg^−1^ DM
**P_Olsen**	P	Extractable	Olsen method	mg kg^−1^ DM
**P_Oxa**	P	Amorphous oxide	Oxalate	mg kg^−1^ DM
**P_Sol_Ca**	P	Soluble	0.01 M Ca(NO_3_)_2_	mg kg^−1^ DM
**P_Tot_Aqu**	P	Total	Aqua regia	mg kg^−1^ DM
**Pb_DTPA**	Pb	Potentially phytoavailable	DTPA	mg kg^−1^ DM
**Pb_Sol_Ca**	Pb	Soluble	0.01 M Ca(NO_3_)_2_	mg kg^−1^ DM
**Pb_Tot_Aqu**	Pb	Total	Aqua regia	mg kg^−1^ DM
**PBI**	PBI	Phosphorus Buffering Index		no unit
**pH_CaNO**	pH	pH measured in 0.01 M Ca(NO_3_)_2_	0.01 M Ca(NO_3_)_2_	no unit
**pH_w**	pH	pH measured in soil:water suspension	Water	no unit
**S_Ads_Seq**	S	Adsorbed	Sequential (0.016 M KH_2_PO_4_)	mg kg^−1^ DM
**S_CaCl**	S	Soluble	0.01 M CaCl_2_	mg kg^−1^ DM
**S_Org_Seq**	S	Organic	Sequential (10% TMAH)	mg kg^−1^ DM
**S_Sol_Seq**	S	Soluble	Sequential (0.01 M KNO_3_)	mg kg^−1^ DM
**S_Tot_Aqu**	S	Total	Aqua regia	mg kg^−1^ DM
**Se_Ads_Seq**	Se	Adsorbed	Sequential (0.016 M KH_2_PO_4_)	µg kg^−1^ DM
**Se_CaCl**	Se	Soluble	0.01 M CaCl_2_	mg kg^−1^ DM
**Se_Org_Seq**	Se	Organic	Sequential (10% TMAH)	µg kg^−1^ DM
**Se_Sol_Seq**	Se	Soluble	Sequential (0.01 M KNO_3_)	µg kg^−1^ DM
**Se_Tot_Aqu**	Se	Total	Aqua regia	mg kg^−1^ DM
**Ti_Tot_Aqu**	Ti	Total	Aqua regia	mg kg^−1^ DM
**U_Sol_Ca**	U	Soluble	0.01 M Ca(NO_3_)_2_	mg kg^−1^ DM
**Zn_CaCl**	Zn	Soluble	0.01 M CaCl_2_	mg kg^−1^ DM
**Zn_DTPA**	Zn	potentially available	DTPA	mg kg^−1^ DM
**Zn_E**	Zn	Isotopically exchangeable	Isotopic dilution	mg kg^−1^ DM
**Zn_FIA**	Zn	Free ion activity	Predicted by geochemical Windermere Humic Aqueous Model (WHAM)	mole L^−1^
**Zn_LogKd**	Zn	Partition coefficient	Isotopic dilution	log (L kg^−1^)
**Zn_Sol_Ca**	Zn	Soluble	0.01 M Ca(NO_3_)_2_	mg kg^−1^ DM
**Zn_Tot_Aqu**	Zn	Total	Aqua regia	mg kg^−1^ DM

DM = dry matter. This field information is presented as separate worksheet or CSV file in the respective workbook or folder for each country.

**Online-only Table 2:** Percentage recovery of elements in the reference soil (WEPAL ISE962) by ICP-OES and ICP-MS following aqua regia acid digestion of soil samples.

			ICP-OES																		ICP-MS				
Country_Project	ICP_Run	AquaRegia_BatchID	Al	As	Ca	Cd	Co	Cr	Cu	Fe	K	Mg	Mn	Na	Ni	P	Pb	S	Ti	Zn	As	Cd	Mo	Pb	Se
ETH-GCRF	set 01	53	86.0	86.4	101.9	202.9	94.0	90.2	83.3	106.5	87.9	99.9	109.9	85.3	104.7	96.0	94.8	99.4	75.9	93.6	n/m^1^	n/m	86.8	n/m	107.3
ETH-GCRF	set 02	60	88.8	84.2	99.3	175.4	93.7	90.5	85.2	106.9	92.4	102.4	110.4	90.7	109.2	95.6	96.6	97.7	65.2	94.6	n/m	n/m	79.6	n/m	119.4
ETH-GCRF	set 03	61	88.7	99.7	102.0	202.4	92.5	88.9	82.5	109.6	89.1	101.2	112.5	82.2	97.9	97.1	95.3	98.9	62.7	94.9	n/m	n/m	73.5	n/m	113.2
ETH-GCRF	set 04	62	89.3	91.8	98.2	185.9	89.8	93.8	87.1	109.2	85.9	99.0	110.4	81.4	100.5	102.7	94.0	95.3	64.5	89.8	n/m	n/m	88.9	n/m	120.3
ETH-GCRF	set 05	64	89.1	85.0	95.9	214.7	88.4	91.7	85.9	107.9	82.9	100.4	109.3	77.0	95.6	97.8	90.5	96.2	80.4	88.4	n/m	n/m	94.3	n/m	128.8
ETH-GCRF	set 06	63	89.6	97.8	97.0	199.4	90.3	92.4	86.2	109.6	84.1	100.9	111.2	77.6	96.7	98.2	92.6	96.0	82.0	88.4	n/m	n/m	97.9	n/m	115.1
ETH-GCRF	set 07	65	93.9	82.8	100.1	197.0	95.3	96.3	87.3	110.3	92.3	106.4	113.4	96.5	91.9	96.2	98.7	96.7	129.4	96.4	n/m	n/m	87.1	n/m	115.9
ETH-GCRF	set 08	66	85.2	93.6	98.1	226.9	102.2	93.9	87.9	105.5	87.5	101.3	108.0	93.1	85.8	98.3	97.2	97.6	84.9	90.2	n/m	n/m	93.9	n/m	123.5
ETH-GCRF	set 09	67	85.2	115.7	97.7	188.5	100.6	94.5	85.2	104.5	88.1	101.6	106.7	83.5	84.3	93.5	95.2	94.3	69.8	87.4	n/m	n/m	76.7	n/m	115.7
ETH-GCRF	set 10	68	92.8	88.0	96.9	179.2	85.9	96.7	86.6	105.7	95.0	105.0	107.0	81.0	88.7	98.4	96.6	95.0	69.4	89.1	n/m	n/m	77.8	n/m	119.0
ETH-GCRF	set 11	187	90.5	112.0	97.8	229.0	94.2	90.2	86.1	106.0	90.6	102.6	107.0	88.6	89.5	93.1	91.2	96.5	82.2	90.0	n/m	n/m	87.5	n/m	120.3
ETH-BMGF	set 01	374	91.1	109.2	96.6	89.5	88.5	94.3	80.9	105.1	87.5	97.4	109.6	76.8	95.1	89.5	176.9	93.2	73.4	100.3	95.3	89.0	75.2	230.5	146.8
ETH-BMGF	set 02	375	95.8	118.3	97.7	92.8	87.4	97.5	90.1	107.0	93.1	106.5	111.6	82.4	98.4	89.2	87.9	95.3	78.6	94.1	93.3	89.2	83.7	84.3	148.3
ETH-BMGF	set 03	376	91.5	103.1	97.4	93.2	86.9	94.3	85.1	107.4	87.6	106.5	112.8	81.5	105.6	91.1	316.0	97.8	51.5	103.2	91.8	87.9	72.8	302.8	128.5
ETH-BMGF	set 04	377	86.7	149.1	94.1	87.8	83.0	93.2	65.7	97.5	91.4	97.4	105.0	71.0	101.8	83.9	85.6	92.2	40.7	89.7	85.6	81.2	74.7	83.3	130.4
ETH-BMGF	set 05	378	90.8	101.8	100.0	93.0	95.1	100.5	70.0	99.1	97.8	98.8	101.3	92.6	106.6	96.0	92.3	96.8	77.5	96.5	94.9	87.3	69.6	89.2	80.3
ETH-BMGF	set 06	379	95.6	99.2	94.7	83.8	84.3	95.5	71.7	109.2	92.6	98.2	112.5	83.8	101.7	84.3	86.7	96.5	75.5	88.1	103.9	94.3	87.2	95.1	166.9
ETH-BMGF	set 07	422	88.0	109.7	98.4	100.8	89.5	94.1	78.6	105.9	88.1	99.7	106.5	87.5	102.4	91.5	97.5	98.6	67.7	92.7	101.9	94.5	76.1	99.2	145.8
ETH-BMGF	set 08	423	93.5	81.3	100.3	73.1	79.4	96.7	75.3	110.6	94.7	102.0	107.9	105.2	97.9	91.1	96.4	89.4	72.6	96.4	100.7	90.8	86.6	100.0	175.2
ETH-BMGF	set 09	424	85.8	137.3	96.2	85.4	91.1	92.4	74.5	102.5	85.8	96.2	104.7	81.0	94.7	90.3	94.8	94.6	58.7	89.7	93.8	90.9	77.8	84.9	122.6
ETH-BMGF	set 10	425	95.3	63.1	103.4	116.3	94.4	93.4	86.5	105.1	94.8	101.9	105.9	96.1	94.9	94.3	93.8	93.9	75.9	90.2	95.5	91.4	88.4	95.9	143.1
ETH-BMGF	set 11	426	94.3	78.1	102.1	80.9	88.1	88.8	86.3	103.5	95.4	101.9	106.7	84.9	89.0	90.6	92.6	92.3	75.6	91.5	103.0	90.6	83.4	91.9	139.1
ETH-BMGF	set 12	427	92.5	84.5	96.6	110.2	94.5	89.8	80.4	105.8	96.2	101.6	108.1	79.6	93.2	87.4	165.9	92.8	80.2	95.8	98.0	93.2	83.0	169.0	124.1
ETH-BMGF	set 13	428	94.2	112.6	99.4	117.5	101.3	88.7	77.3	106.3	98.0	100.6	106.2	92.1	94.6	93.2	91.7	98.6	72.8	90.5	98.4	90.2	81.3	94.5	125.4
ETH-BMGF	set 14	429	94.0	85.5	96.6	86.1	90.1	87.3	86.5	109.8	93.1	99.4	110.5	87.0	89.9	83.0	89.5	94.5	68.6	91.6	96.9	92.4	79.0	95.5	121.2
ETH-BMGF	set 15	430	89.2	76.0	100.2	99.5	96.1	95.2	88.5	104.8	91.1	101.5	109.1	90.9	103.6	88.3	109.0	96.5	60.5	91.2	101.1	92.6	80.5	93.2	159.7
ETH-BMGF	set 16	431	94.6	92.3	97.2	103.3	93.6	94.9	85.2	109.1	93.2	101.6	110.9	85.6	103.3	89.7	95.3	97.7	64.0	92.1	97.3	90.7	78.6	91.2	125.5
ETH-BMGF	set 17	432	93.9	102.2	97.7	113.3	92.2	95.2	87.8	114.6	92.9	107.1	117.6	86.1	95.9	96.4	99.2	99.0	60.4	99.1	97.6	95.4	77.7	90.4	126.2
ETH-BMGF	set 18	433	89.5	91.9	95.2	70.3	94.2	93.2	86.3	108.1	86.8	100.0	113.2	85.8	90.5	94.9	91.9	96.7	55.3	97.3	95.5	88.8	78.8	99.8	150.3
ETH-BMGF	set 19	434	92.8	131.5	100.0	91.6	87.3	97.0	81.7	105.0	94.3	101.7	110.2	88.3	101.2	93.7	106.0	98.8	65.0	96.6	101.0	92.4	81.7	90.4	167.2
ETH-BMGF	set 20	435	92.1	109.7	98.8	72.5	101.2	92.6	92.1	116.7	89.7	106.5	121.5	86.5	96.9	96.3	100.9	102.8	56.5	92.4	92.6	83.5	75.4	82.4	137.9
ETH-BMGF	set 21	436	100.3	72.3	96.5	60.1	89.6	95.7	83.9	110.9	94.5	110.6	115.6	87.1	95.2	87.9	89.6	97.2	71.5	91.2	101.4	90.9	85.8	93.6	153.0
ETH-BMGF	set 22	437	90.2	84.5	99.5	79.9	92.3	95.8	87.6	105.5	91.7	99.2	110.5	80.7	104.0	93.0	92.6	100.2	105.6	95.8	95.5	94.3	95.8	94.8	136.6
MWI-BMGF	set 01	457	89.0	84.0	101.6	114.8	91.0	92.6	84.6	106.3	88.8	100.1	103.0	85.7	106.3	93.6	96.7	98.0	63.0	87.6	94.0	88.5	74.9	96.8	133.8
MWI-BMGF	set 02	458	90.0	75.7	97.5	99.2	88.2	95.1	88.0	108.5	87.9	100.2	107.3	81.2	104.6	92.8	91.5	99.6	70.3	89.6	95.0	90.0	86.9	86.7	130.8
MWI-BMGF	set 03	664	89.8	60.9	102.5	108.5	97.9	96.9	94.8	106.4	94.4	99.5	103.7	93.6	99.8	94.0	98.9	100.5	55.6	91.4	91.1	93.7	74.8	88.0	129.7
MWI-BMGF	set 04	460	89.6	74.5	101.5	95.5	100.8	93.7	88.0	104.6	93.1	101.8	107.3	93.3	99.1	95.4	100.9	97.4	71.7	86.6	96.5	88.4	87.9	96.2	156.3
MWI-BMGF	set 05	461	89.8	80.7	102.0	108.8	92.8	95.7	84.3	101.3	91.0	101.5	100.1	78.4	87.3	93.3	97.5	99.1	62.7	91.5	96.2	102.1	68.8	94.8	148.3
MWI-BMGF	set 06	462	86.6	73.8	97.0	97.2	98.5	92.0	87.3	100.8	89.8	100.0	101.9	88.5	96.8	93.9	96.2	99.1	72.1	91.9	91.0	88.7	86.3	84.6	137.5
MWI-BMGF	set 07	463	87.7	75.6	99.8	94.5	95.2	87.8	81.9	108.1	86.2	100.1	106.8	75.8	95.0	91.8	96.1	96.7	67.5	83.7	96.8	84.4	82.1	100.7	147.2
MWI-BMGF	set 08	464	92.5	79.4	100.8	102.0	106.0	98.8	92.0	104.9	94.8	102.5	107.9	90.6	105.1	99.6	103.5	101.9	31.7	98.1	91.6	88.5	71.6	110.4	138.5
MWI-BMGF	set 09	465	88.2	84.7	96.5	109.8	107.8	96.4	89.6	103.0	95.5	98.6	103.6	196.8	102.0	95.5	102.1	100.2	90.2	95.5	92.8	90.9	87.0	106.4	142.7
MWI-BMGF	set 10	466	97.4	80.2	100.7	99.8	108.0	103.2	95.3	112.1	98.8	103.2	109.7	90.8	108.5	97.3	108.4	101.5	82.8	97.6	98.0	92.5	89.2	96.1	139.7
MWI-BMGF	set 11	680	89.0	62.3	101.9	109.1	99.1	90.8	87.4	107.0	94.1	102.1	100.8	93.9	96.3	93.3	94.1	99.4	56.9	94.1	93.0	85.0	71.0	94.9	130.4
MWI-BMGF	set 12	681	85.5	53.5	93.6	71.4	90.1	80.4	82.7	102.1	87.0	99.2	97.8	71.8	85.0	88.9	81.5	93.1	87.2	84.8	92.8	83.0	86.8	93.8	126.5
MWI-BMGF	set 13	687	93.7	68.2	102.8	113.7	101.8	92.2	87.1	113.3	95.3	107.0	118.0	87.7	97.8	94.7	92.6	100.5	74.8	86.5	96.9	86.4	78.9	88.1	117.5
MWI-BMGF	set 14	689	89.2	71.2	100.2	121.6	102.7	94.0	98.0	109.0	90.3	103.5	110.6	84.6	98.7	95.5	99.7	99.8	99.8	87.7	93.3	82.6	85.1	93.2	130.1
MWI-BMGF	set 15	690	90.0	67.6	97.9	98.8	95.3	90.1	95.1	106.6	91.9	102.8	110.1	86.0	94.0	91.2	99.5	99.0	72.2	81.0	97.9	84.7	100.3	102.1	185.6
MWI-BMGF	set 16	691	85.6	65.8	99.2	102.6	98.8	91.5	94.5	103.6	87.5	100.5	104.6	83.7	96.7	92.7	94.7	94.9	64.4	84.2	91.2	81.2	70.7	93.5	120.6
MWI-BMGF	set 17	692	86.5	56.9	97.9	104.6	95.8	90.7	93.6	104.9	88.5	101.6	109.1	90.6	96.2	91.6	93.1	91.0	59.5	79.1	91.8	81.7	74.7	87.9	127.2
MWI-BMGF	set 18	693	86.7	59.3	96.4	91.8	98.9	93.2	100.2	101.5	90.7	100.4	103.4	94.4	98.3	90.0	95.1	96.2	74.6	82.2	95.8	86.5	90.5	91.2	187.5
MWI-BMGF	set 19	694	94.0	49.3	97.2	108.1	105.4	103.4	90.9	104.9	98.5	102.7	107.5	97.6	102.4	94.5	103.4	97.0	78.6	93.1	85.6	93.5	87.2	90.6	137.5
MWI-BMGF	set 20	694	88.4	71.5	101.8	90.1	102.3	95.6	89.5	105.9	88.9	100.3	108.1	86.8	101.2	96.5	101.2	101.1	73.4	87.3	93.2	81.7	80.4	100.1	174.4
MWI-BMGF	set 21	695	92.3	65.0	101.0	113.6	101.2	97.6	92.1	105.1	94.1	100.9	108.2	86.9	101.2	96.5	101.2	99.7	69.5	87.7	93.1	81.9	72.3	88.2	165.3
MWI-BMGF	set 22	696	85.0	54.4	101.4	113.5	101.8	93.3	99.0	104.1	90.1	99.9	109.0	87.0	98.5	96.9	96.9	101.4	58.3	94.0	89.9	82.5	82.2	97.1	164.3
MWI-BMGF	set 23	697	89.7	61.1	94.5	96.1	99.0	91.3	86.3	104.4	92.2	100.0	107.6	81.6	95.3	92.8	95.2	95.1	84.2	98.7	89.9	82.5	82.2	97.1	164.3
MWI-BMGF	set 24	698	95.5	54.8	96.0	109.5	99.9	94.0	92.6	106.4	101.3	102.0	109.2	97.1	95.9	97.9	100.2	99.6	79.4	95.4	94.6	94.1	81.5	97.6	160.3
MWI-BMGF	set 25	699	93.6	48.6	101.8	94.6	103.6	96.1	100.9	110.5	96.5	105.0	109.4	86.7	99.9	100.3	97.6	100.9	79.4	95.0	93.6	82.2	85.8	93.7	157.9
MWI-BMGF	set 26	700	91.4	63.7	101.4	103.9	105.2	96.2	102.7	109.9	94.3	106.2	112.6	92.9	102.5	102.0	101.9	103.2	84.2	98.8	93.4	84.8	87.0	97.1	154.3
MWI-BMGF	set 27	701	93.4	68.9	101.1	109.2	104.7	95.6	100.3	108.7	98.5	102.7	108.6	92.6	98.2	99.0	97.1	99.2	74.9	94.7	91.8	86.6	82.6	93.8	174.8
MWI-BMGF	set 28	702	84.5	48.0	98.7	103.0	97.2	92.5	86.9	105.5	86.0	99.3	106.7	82.5	89.8	92.9	99.6	95.2	52.5	94.2	91.4	84.2	77.1	95.8	174.7
MWI-BMGF	set 29	703	85.5	60.0	100.7	92.2	100.3	94.6	87.2	104.3	88.8	98.5	104.0	91.2	99.7	94.6	102.5	99.9	69.0	97.7	93.9	84.0	83.3	98.8	174.1
MWI-BMGF	set 30	704	87.5	63.6	99.1	105.8	99.9	90.7	87.6	105.4	90.9	100.8	105.9	86.8	96.0	92.9	95.6	0.0	58.6	93.3	92.2	85.7	75.3	99.0	166.7
MWI-BMGF	set 31	705	90.6	66.0	103.7	127.1	95.1	95.3	84.0	110.3	91.2	103.0	108.3	86.4	98.7	97.5	99.5	99.8	72.1	92.4	93.2	83.4	81.0	92.5	162.1
MWI-BMGF	set 32	706	93.4	76.4	96.8	97.1	92.0	95.5	90.1	107.6	93.1	103.0	108.9	100.3	95.8	91.6	96.8	99.2	44.6	84.5	93.0	83.1	73.1	100.4	166.8
MWI-BMGF	set 33	707	83.3	48.2	100.1	82.1	88.5	89.0	74.8	101.0	86.2	97.8	105.8	90.2	88.5	89.1	92.6	94.6	48.3	84.1	86.6	83.5	71.3	92.2	151.2
MWI-BMGF	set 34	708	94.9	55.3	102.6	105.9	98.4	99.7	93.0	111.3	98.6	102.7	105.0	90.1	100.7	95.7	98.6	99.4	68.8	94.9	90.6	83.5	69.3	88.8	125.7
MWI-BMGF	set 35	709	85.5	52.9	100.5	118.6	96.8	96.2	84.9	104.7	87.1	97.6	105.3	83.6	95.8	93.8	93.2	96.8	52.7	94.3	94.9	90.2	76.7	102.9	160.6
MWI-BMGF	set 36	710	88.6	37.2	99.9	97.4	97.9	98.2	82.6	104.4	94.0	99.0	105.2	89.0	94.9	93.3	93.9	97.3	69.2	95.7	95.7	92.0	82.7	105.1	155.1
MWI-BMGF	set 37	711	87.2	51.3	98.8	93.9	95.7	98.4	90.6	105.1	88.8	97.1	107.3	84.4	97.8	91.8	98.0	100.5	66.1	93.7	95.9	87.7	83.2	101.9	145.8
MWI-BMGF	set 38	712	89.6	57.4	100.1	89.2	94.6	96.1	86.0	108.0	88.8	103.9	111.4	83.4	98.1	92.5	96.6	97.5	61.4	104.9	94.9	96.8	83.3	95.0	153.9
MWI-BMGF	set 39	713	86.4	41.3	98.3	89.8	94.1	93.5	78.8	105.0	84.9	96.6	109.0	81.3	97.7	93.3	96.6	101.7	56.4	94.7	95.3	87.7	77.8	102.1	174.7
MWI-BMGF	set 40	714	90.9	49.5	95.6	80.6	93.3	95.4	79.7	106.7	88.4	103.1	108.9	90.4	97.3	89.3	91.2	98.7	60.6	91.0	93.7	87.6	104.9	94.3	180.7
MWI-BMGF	set 41	715	89.3	53.4	98.6	89.6	95.4	95.8	84.7	105.3	88.0	95.9	105.6	82.9	97.6	93.0	94.5	101.0	66.1	95.7	95.8	87.0	86.5	88.3	181.3
MWI-BMGF	set 42	754	93.1	72.5	100.0	95.8	101.7	96.4	89.5	106.1	98.0	103.0	101.9	92.8	97.9	93.3	94.4	99.3	78.7	93.9	90.1	88.1	82.7	96.2	129.4
n/m^1^	These measurements were not made by ICP-MS in ETH-GCRF																								

ICP Run is the ICP run number and Batch/n is the lab batch number and number of samples. All values represent results following subtraction of blank values for each element. ETH = Ethiopia, MWI = Malawi, BMGF = Bill & Melinda Gates Foundation, GCRF = Global Challenges Research Fund.

**Online-only Table 3:** Comparison of mean, median, errors and average % recovery between this study and WEPAL for reference soil (ISE962) by ICP-OES and ICP-MS following aqua regia acid digestion.

			ICP-OES																																			ICP-MS									
Element		Al		As		Ca		Cd		Co		Cr		Cu		Fe		K		Mg		Mn		Na		Ni		P		Pb		S		Ti		Zn		As		Cd		Mo		Pb		Se	
		WEPAL	This	WEPAL	This	WEPAL	This	WEPAL	This	WEPAL	This	WEPAL	This	WEPAL	This	WEPAL	This	WEPAL	This	WEPAL	This	WEPAL	This	WEPAL	This	WEPAL	This	WEPAL	This	WEPAL	This	WEPAL	This	WEPAL^2^	This	WEPAL	This	WEPAL	This	WEPAL	This	WEPAL	This	WEPAL	This	WEPAL^3^	This
Median	mg/kg	27000	23968	12	9	36500	35959	0.24	0.24	9.75	9.28	46.0	43.7	13.2	11.4	31100	32954	6240	5791	9320	9462	921	1001	244	212	30.1	29.4	726	679	30.1	28.7	1850	1798	380	259	85.2	79.1	12.0	11.3	0.24	0.21	0.44	0.37	30.1	28.3	0.29	0.39
Mean	mg/kg	26700	24106	12	9	36300	35929	0.24	0.27	9.75	9.31	46.3	43.5	13.2	11.4	31100	33114	6350	5807	9340	9467	926	1001	244	215	30.1	29.3	727	680	29.8	30.1	1840	1773	372	260	85.6	78.9	12.0	11.3	0.24	0.21	0.45	0.36	29.8	30.1	0.28	0.40
Std.Dev.	mg/kg	2920	929	1.28	3	1710	864	0.03	0.09	0.66	0.59	5.31	1.66	1.3	0.9	2470	1035	792	257	725	263	58.4	37	29.9	35	2.28	1.62	47	28	3.03	8.5	105	214	93.9	52.9	5.22	4.2	1.28	0.45	0.03	0.01	0.08	0.03	3.03	9.7	0.06	0.06
CV%		11	4	11	30	5	2	14	35	7	6	12	4	10	8	8	3	13	4	8	3	6	4	12	16	8	6	7	4	10	28	6	12	25	20	6	5	11	4	14	5	18	9	10	32	23	15
N^1^		75	75	103	75	87	75	107	75	94	75	130	75	141	75	98	75	81	75	86	75	102	75	66	75	133	75	84	75	135	75	67	75	30	75	139	75	103	64	107	64	47	75	135	64	34	75
Average % Recovery cf mean			90		78		99		113		95		94		87		106		91		101		108		88		97		94		101		96		70		92		95		88		82		101		143
Average % Recovery cf median			89		78		98		112		95		95		87		106		93		102		109		88		97		94		100		96		68		93		95		87		82		100		138

^1^WEPAL report results with outliers removed; N = no of lab results accepted

^2^WEPAL give indicative values only for Ti

^3^WEPAL includes a mixture of ICP-OES and -MS results for Se; on OES we found that Se values were lower but had greater error

**Online-only Table 4:** Limits of detection (3x standard deviation of blank samples expressed as mg kg^−1^ assuming sample 0.25g vol 25 mL), and number of blanks analysed after aqua regia extraction of soils.

			ICP-OES (mg/kg)																		ICP-MS (mg/kg)				
			Al	As	Ca	Cd	Co	Cr	Cu	Fe	K	Mg	Mn	Na	Ni	P	Pb	S	Ti	Zn	As	Cd	Mo	Pb	Se
LOD (Blank Stdev x3)			353.83	2.59	761.24	0.10	0.74	0.67	1.08	179.49	140.52	16.28	4.28	99.26	2.10	21.56	0.79	16.79	87.92	2.89	0.13	0.02	0.07	0.37	0.04
		N	75	75	75	75	75	75	75	75	75	75	75	75	75	75	75	75	75	75	75	75	75	75	75

**Online-only Table 5:** Elemental concentrations in blanks used in for aqua regia extraction of soils (average two blanks in each batch). ETH = Ethiopia, MWI = Malawi, BMGF = Bill & Melinda Gates Foundation, GCRF = Global Challenges Research Fund.

All elements blank subtracted																					
Average of two blanks in each batch				ICP-OES (mg/kg)																	
Country_Project	ICP_Run	AY	AquaRegia_BatchID	Al	As	Ca	Cd	Co	Cr	Cu	Fe	K	Mg	Mn	Na	Ni	P	Pb	S	Ti	Zn
ETH-GCRF	set 01	2018/19	53	−178.17	−1.68	55.06	−0.03	0.42	0.05	0.63	−268.58	9.96	8.98	−6.20	36.25	2.02	3.42	0.37	7.65	1.37	1.74
ETH-GCRF	set 02	2018/19	60	−178.60	−0.96	58.83	0.01	0.10	0.03	0.14	−237.11	3.63	12.97	−6.13	25.56	0.11	−1.96	0.45	7.41	16.88	0.54
ETH-GCRF	set 03	2018/19	61	1.71	−0.60	62.65	−0.06	−0.05	−0.17	−0.12	6.74	−11.91	11.41	−1.34	31.06	−0.10	−13.61	−0.40	5.98	69.55	0.38
ETH-GCRF	set 04	2018/19	62	13.61	1.13	50.36	−0.04	−0.19	−0.11	−0.05	24.22	−14.07	5.64	−0.14	30.36	0.29	−17.80	0.11	7.10	2.89	0.68
ETH-GCRF	set 05	2018/19	63	−42.95	1.84	61.84	−0.01	−0.08	−0.14	0.07	−70.11	−5.50	11.88	−2.34	27.72	−0.11	−16.70	−0.04	1.18	34.07	0.99
ETH-GCRF	set 06	2018/19	64	−34.17	0.05	25.84	−0.04	−0.09	0.47	0.07	−66.59	−10.95	0.73	−0.52	38.12	0.32	−14.87	−0.17	−0.48	47.82	0.80
ETH-GCRF	set 07	2018/19	65	−0.40	0.00	0.60	0.00	0.00	0.00	0.00	−0.60	−0.03	0.13	−0.02	0.26	0.00	0.00	0.00	0.07	0.90	0.02
ETH-GCRF	set 08	2018/19	66	53.99	−1.25	50.52	0.02	0.05	0.01	0.50	55.04	−9.65	7.21	1.52	26.39	0.04	−4.48	0.28	1.08	2.26	0.81
ETH-GCRF	set 09	2018/19	67	−52.10	1.45	18.96	−0.02	−0.18	−0.05	−0.12	−105.68	−16.31	2.32	−2.43	29.46	−0.19	−9.86	−0.01	0.08	9.90	0.85
ETH-GCRF	set 10	2018/19	68	−26.35	0.75	18.67	−0.04	−0.10	−0.08	−0.04	−62.91	−19.08	1.71	−2.19	30.02	−0.10	−13.03	0.20	4.58	55.78	0.60
ETH-GCRF	set 11	2018/19	187	−12.18	1.12	74.32	0.01	−0.32	0.20	0.28	7.92	14.18	15.29	−0.21	36.77	0.41	0.24	0.79	12.64	11.63	1.36
ETH-BMGF	set 01	2019/20	374	54.44	−0.38	335.92	0.02	0.08	0.28	0.74	−3.13	110.71	18.87	0.61	46.42	0.21	1.15	0.41	2.09	31.35	2.08
ETH-BMGF	set 02	2019/20	375	32.02	−0.63	57.61	0.04	−0.06	−0.01	1.06	29.13	43.87	10.88	0.30	35.02	−0.03	−0.03	−0.14	2.60	8.30	4.51
ETH-BMGF	set 03	2019/20	376	154.27	0.82	679.91	−0.03	0.03	0.07	0.28	5.86	−5.80	12.28	0.57	33.05	−0.03	−6.21	0.08	6.81	41.87	1.17
ETH-BMGF	set 04	2019/20	377	−99.66	2.56	22.10	−0.04	−0.09	−0.14	1.24	−123.41	193.06	5.77	0.23	62.18	−0.29	−13.89	−0.62	−1.93	64.08	2.15
ETH-BMGF	set 05	2019/20	378	−101.22	0.95	33.97	−0.03	−0.08	−0.04	1.24	−131.08	62.92	9.75	−4.73	26.59	−0.08	−5.83	0.45	−0.23	0.06	2.80
ETH-BMGF	set 06	2019/20	379	−31.69	0.38	79.19	−0.05	−0.13	−0.13	0.75	−58.14	30.41	8.24	−2.36	23.93	−0.26	−10.72	0.31	1.58	19.78	3.85
ETH-BMGF	set 07	2019/20	422	13.98	0.17	28.67	−0.06	−0.07	−0.09	0.53	14.82	125.71	5.71	0.45	38.80	−0.09	−12.54	0.18	−0.38	28.15	0.61
ETH-BMGF	set 08	2019/20	423	572.54	−0.15	1334.38	−0.05	−0.26	0.22	1.36	15.51	99.80	25.49	0.84	58.60	−0.19	−16.58	−0.05	−2.44	14.58	4.27
ETH-BMGF	set 09	2019/20	424	12.21	−0.95	75.65	−0.04	−0.19	−0.19	1.19	4.23	141.65	6.08	0.15	32.71	−0.14	−12.56	−0.13	−5.11	15.92	3.09
ETH-BMGF	set 10	2019/20	425	52.21	0.02	47.05	−0.04	−0.11	−0.04	0.10	60.68	−1.88	11.11	0.78	23.41	−0.02	−4.02	0.26	3.25	7.96	0.89
ETH-BMGF	set 11	2019/20	426	139.79	−0.06	272.34	0.02	−0.09	0.01	0.20	38.77	10.67	14.10	1.34	35.54	−0.07	−3.97	0.10	11.48	14.92	2.20
ETH-BMGF	set 12	2019/20	427	445.10	0.72	1172.30	0.00	−0.07	0.45	0.38	−54.08	19.29	28.79	−1.73	59.37	−0.07	−3.51	0.11	−0.99	−1.12	3.33
ETH-BMGF	set 13	2019/20	428	55.18	0.27	157.28	−0.01	−0.27	−0.01	1.00	51.49	12.37	25.88	2.14	29.27	−0.02	−14.23	0.09	−0.68	1.27	1.22
ETH-BMGF	set 14	2019/20	429	−58.10	−0.27	242.20	−0.07	−0.28	−0.05	0.07	−141.76	−9.91	4.72	0.36	17.82	−0.21	−4.16	0.31	−3.71	14.07	3.42
ETH-BMGF	set 15	2019/20	430	46.88	−0.76	28.63	0.01	−0.07	−0.13	0.14	60.39	5.80	4.84	1.26	21.83	−0.06	−5.22	0.06	1.87	14.30	1.38
ETH-BMGF	set 16	2019/20	431	95.76	−0.63	59.41	0.01	0.06	0.18	0.12	112.53	−10.58	14.65	0.33	23.93	−0.18	−13.98	−0.17	1.33	4.47	0.78
ETH-BMGF	set 17	2019/20	432	31.48	−0.84	39.72	−0.05	−0.15	0.10	0.14	28.32	−8.11	9.30	0.21	31.63	0.03	−8.89	0.13	−1.77	10.42	0.94
ETH-BMGF	set 18	2019/20	433	−50.89	−0.70	44.56	−0.04	−0.03	0.05	0.24	−79.47	10.78	10.57	0.27	24.21	−0.19	−11.67	−0.26	−4.05	11.57	1.32
ETH-BMGF	set 19	2019/20	434	−11.87	−3.98	28.14	−0.08	−0.45	−0.19	−0.07	−15.29	−18.59	7.57	0.15	20.55	−0.16	−19.53	−0.23	−0.50	11.94	1.79
ETH-BMGF	set 20	2019/20	435	19.29	−0.33	65.97	−0.11	−0.06	−0.32	−0.09	5.45	−29.72	7.18	−0.67	20.83	−0.39	−23.43	−0.39	−4.97	9.74	0.14
ETH-BMGF	set 21	2019/20	436	165.34	−1.19	301.72	−0.13	−0.41	−0.35	−0.06	64.33	−25.71	13.85	0.04	24.96	−0.55	−26.96	−0.38	−6.69	0.44	1.97
ETH-BMGF	set 22	2019/20	437	70.14	−0.34	165.45	0.01	0.06	0.04	0.07	54.31	−11.90	13.55	0.20	29.21	−0.13	−7.05	0.41	1.87	129.67	1.39
MWI-BMGF	set 01	2018/19	457	−6.96	0.01	29.82	−0.01	−0.55	−0.13	0.25	6.75	36.19	5.79	−0.07	35.30	−0.02	−7.40	0.24	6.40	19.25	2.28
MWI-BMGF	set 02	2018/19	458	111.41	0.41	302.40	−0.02	−0.57	0.04	0.01	13.97	−10.99	6.32	−0.06	40.05	0.12	−10.98	−0.16	3.60	7.89	0.89
MWI-BMGF	set 03	2018/19	664	8.93	0.13	82.96	−0.01	0.07	0.02	0.32	15.27	2.64	15.58	−0.19	37.20	−0.11	−6.60	0.21	2.08	58.24	0.55
MWI-BMGF	set 04	2018/19	460	6.35	−0.49	57.04	−0.01	−0.22	0.02	−0.10	−8.00	−3.20	4.64	0.07	29.60	0.05	−3.32	−0.09	5.47	−4.71	0.73
MWI-BMGF	set 05	2018/19	461	55.53	0.34	134.76	0.01	1.58	0.26	0.55	14.25	237.76	12.85	0.67	302.06	4.20	6.96	0.38	16.70	41.93	1.51
MWI-BMGF	set 06	2018/19	462	13.23	0.20	68.01	−0.01	−0.04	0.00	0.13	19.95	1.53	9.56	0.35	53.04	0.29	−3.79	0.08	4.72	5.50	0.24
MWI-BMGF	set 07	2018/19	463	365.82	−0.25	827.32	0.02	0.05	0.39	0.46	37.15	7.42	16.85	0.43	65.59	0.19	0.07	0.08	8.04	26.86	0.39
MWI-BMGF	set 08	2018/19	464	20.69	0.24	43.75	0.02	0.15	0.22	0.25	32.73	−2.88	10.23	0.60	30.13	−0.04	1.43	0.58	5.48	161.55	0.38
MWI-BMGF	set 09	2018/19	465	473.36	−0.02	1090.30	−0.01	−0.09	0.42	0.54	16.47	11.48	15.16	0.55	67.50	0.32	−2.02	0.43	6.41	18.66	1.13
MWI-BMGF	set 10	2018/19	466	−8.76	−0.40	64.53	0.03	−0.09	0.24	0.23	−14.59	23.56	14.33	0.76	48.40	0.05	−7.16	0.56	5.27	1.41	0.64
MWI-BMGF	set 11	2018/19	680	42.57	0.80	147.46	−0.03	0.06	0.41	0.48	−5.45	10.24	9.47	−0.17	38.29	0.02	−4.73	0.22	0.64	45.55	0.66
MWI-BMGF	set 12	2018/19	681	19.12	−0.23	73.38	0.04	0.11	1.31	0.20	19.63	19.61	7.00	0.13	63.14	0.66	−0.82	0.45	5.86	1.45	1.32
MWI-BMGF	set 13	2018/19	686	51.86	0.14	90.40	0.04	0.15	0.14	0.19	36.59	19.45	7.92	0.03	41.18	0.11	4.87	0.62	25.78	29.86	0.87
MWI-BMGF	set 14	2018/19	687	32.15	0.48	110.53	0.00	0.06	0.14	0.32	6.75	11.04	13.73	−0.02	47.74	0.28	1.43	0.28	4.11	12.88	0.76
MWI-BMGF	set 15	2018/19	689	4.55	0.18	50.67	0.02	−0.14	−0.09	0.14	7.92	1.53	11.65	−0.54	33.05	−0.08	−15.17	0.34	10.00	14.14	0.49
MWI-BMGF	set 16	2018/19	690	0.99	−0.17	40.54	0.00	0.01	0.09	0.35	−8.40	14.90	5.65	−0.29	35.98	0.10	−2.56	0.12	14.62	12.88	0.60
MWI-BMGF	set 17	2018/19	691	7.72	0.15	36.45	0.00	−0.01	−0.02	0.18	−3.25	1.05	4.90	0.13	29.44	0.04	−6.59	−0.06	7.37	14.26	0.84
MWI-BMGF	set 18	2018/19	692	0.91	0.31	7.36	0.01	−0.10	−0.05	0.22	−0.40	−0.52	0.82	0.02	12.87	−0.09	−8.74	0.18	10.82	9.05	0.77
MWI-BMGF	set 19	2018/19	693	−18.31	0.17	63.09	−0.02	−0.05	−0.01	0.36	−31.92	−8.06	12.26	−1.79	34.38	−0.09	−5.62	0.10	0.15	−2.63	0.42
MWI-BMGF	set 20	2018/19	694	23.42	0.00	30.43	−0.02	−0.11	−0.02	0.19	0.88	−10.68	7.49	0.14	28.49	−0.07	−12.88	0.30	1.68	4.20	0.47
MWI-BMGF	set 21	2018/19	695	5.29	0.20	43.75	−0.01	−0.16	−0.03	0.29	6.94	3.82	8.96	0.05	33.11	−0.20	−14.12	0.25	5.73	−0.26	3.04
MWI-BMGF	set 22	2018/19	696	12.90	−0.03	58.99	0.01	0.02	0.04	0.28	21.60	8.09	12.20	−2.70	30.33	−0.08	−3.13	0.43	0.87	27.70	0.51
MWI-BMGF	set 23	2018/19	697	53.06	0.23	181.70	0.02	0.02	0.07	0.64	7.90	85.86	11.43	0.13	41.11	0.04	−0.74	0.28	0.41	12.76	1.45
MWI-BMGF	set 24	2018/19	698	−3.33	0.45	43.05	0.00	−0.06	0.01	0.21	−11.56	−2.87	11.29	0.12	27.89	0.00	−1.83	−0.06	−2.40	1.35	0.98
MWI-BMGF	set 25	2018/19	699	24.66	−0.26	100.49	−0.02	0.09	0.23	0.38	17.69	41.60	15.56	−0.22	42.45	0.01	4.20	0.67	4.01	10.02	0.87
MWI-BMGF	set 26	2018/19	700	12.77	−0.12	71.82	0.01	−0.01	0.48	0.28	17.12	29.03	15.82	−0.16	39.65	0.27	−1.42	0.35	6.04	7.81	0.52
MWI-BMGF	set 27	2018/19	701	0.76	0.11	59.88	0.01	0.09	0.15	0.27	0.70	31.00	14.14	−0.76	43.35	2.28	−2.07	0.21	7.16	30.79	0.83
MWI-BMGF	set 28	2018/19	702	0.78	0.70	59.63	0.00	−0.04	0.15	0.21	7.30	63.84	12.44	0.53	40.30	2.80	2.31	0.36	3.03	49.82	0.67
MWI-BMGF	set 29	2018/19	703	28.18	−0.22	106.87	−0.04	−0.20	0.10	0.23	−0.17	39.26	7.92	0.43	21.82	−0.06	−6.27	0.28	3.93	1.51	0.06
MWI-BMGF	set 30	2018/19	704	14.72	−0.93	70.75	0.00	0.03	0.08	0.40	23.64	13.17	14.12	0.50	29.02	0.09	4.34	0.02	−11.25	21.43	0.75
MWI-BMGF	set 31	2018/19	705	25.50	−1.04	80.82	−0.03	−0.02	0.06	1.08	23.95	7.93	14.44	0.03	57.17	0.08	−1.66	0.43	5.31	0.83	1.04
MWI-BMGF	set 32	2018/19	706	−0.70	−1.00	46.13	0.03	0.06	0.19	0.37	1.51	−1.44	11.87	0.66	35.75	0.03	−3.58	0.49	0.68	47.81	0.42
MWI-BMGF	set 33	2018/19	707	−8.32	0.51	60.30	0.00	0.09	0.16	1.43	9.15	3.58	14.01	0.97	32.21	0.93	−0.75	0.05	−0.98	35.22	1.01
MWI-BMGF	set 34	2018/19	708	−15.23	−1.86	43.89	−0.02	−0.04	0.00	0.51	−36.67	67.14	7.73	0.02	39.38	−0.01	−2.54	0.12	4.78	8.52	0.85
MWI-BMGF	set 35	2018/19	709	9.93	0.38	69.89	0.01	0.29	0.21	0.36	14.35	16.71	12.81	0.37	40.26	0.16	12.73	0.03	8.27	109.21	1.27
MWI-BMGF	set 36	2018/19	710	−2.73	−0.64	46.94	0.01	0.08	0.06	0.23	1.52	−0.13	10.85	0.33	31.73	0.03	−0.36	0.06	1.60	54.14	0.88
MWI-BMGF	set 37	2018/19	711	−39.01	0.20	48.01	0.02	−0.04	0.08	0.26	−39.30	37.44	8.87	−0.07	31.83	0.35	−4.42	−0.02	0.13	5.81	1.39
MWI-BMGF	set 38	2018/19	712	14.90	−0.06	31.89	−0.03	0.00	0.00	0.00	0.00	0.00	0.00	0.00	31.48	−0.12	−7.45	0.19	−0.49	8.60	1.55
MWI-BMGF	set 39	2018/19	713	0.63	−0.38	34.92	−0.02	0.05	0.00	0.61	−1.24	49.95	7.48	0.07	27.18	−0.10	−2.13	0.28	−2.01	10.68	1.07
MWI-BMGF	set 40	2018/19	714	4.28	−0.79	36.50	−0.04	0.02	−0.05	0.20	−2.45	15.80	6.01	0.01	35.55	−0.09	−1.46	0.19	−3.06	18.65	0.62
MWI-BMGF	set 41	2018/19	715	9.23	−0.47	34.82	−0.04	−0.02	0.02	0.08	2.67	−8.32	7.97	0.07	29.00	−0.10	−6.26	0.17	−2.93	31.94	0.45
MWI-BMGF	set 42	2018/19	754	53.57	0.16	133.66	−0.06	−0.08	−0.14	0.48	24.40	−23.54	5.42	−0.03	30.87	−0.19	−16.35	0.02	−3.62	61.94	1.63

**Online-only Table 6:** Limits of detection (3x standard deviation of blank samples expressed as mg kg^−1^) and number (n) of blanks analysed for soil effective cation exchange capacity (eCEC) and exchangeable cations.

			CoHex eCEC (cmolc/kg)				
			Ca	K	Mg	Na	eCEC
**LOD (Blank Stdev x3)**			1.95	0.02	0.09	0.01	2.88
		**N**	69	69	69	69	69

**Online-only Table 7:** Elemental concentrations (mg kg^−1^) in blanks used for soil eCEC and exchangeable cations determination (average two blanks in each batch). ETH = Ethiopia, MWI = Malawi, BMGF = Bill & Melinda Gates Foundation, GCRF = Global Challenges Research Fund.

Not blank corrected								
Average of two blanks in each batch				CoHex eCEC (cmolc/kg)				
Country_Project	ICP_Run	AY	eCEC_BatchID	Ca	K	Mg	Na	eCEC
ETH-GCRF	set01	2018/19	39	0.118	0.013	0.007	0.014	1.372
ETH-GCRF	set02	2018/19	40	0.133	0.041	0.010	0.015	−1.026
ETH-GCRF	set03	2018/19	41	0.110	0.002	0.006	0.014	1.989
ETH-GCRF	set04	2018/19	42	0.122	−0.009	0.009	−0.002	−0.824
ETH-GCRF	set05	2018/19	43	0.124	0.004	0.012	0.011	−3.733
ETH-GCRF	set06	2018/19	44	0.116	0.002	0.008	0.013	−0.632
ETH-GCRF	set07	2018/19	46	0.123	0.003	0.004	0.014	−1.910
ETH-GCRF	set08	2018/19	47	0.102	−0.001	0.002	0.011	−0.414
ETH-GCRF	set09	2018/19	48	0.107	0.000	0.003	0.012	−0.686
ETH-GCRF	set10	2018/19	49	0.115	0.001	0.003	0.012	−0.627
ETH-GCRF	set11	2018/19	186	0.179	−0.005	0.025	0.013	−0.443
ETH-BMGF	set01	2019/20	501	0.101	0.001	0.008	0.014	−0.641
ETH-BMGF	set02	2019/20	502	0.086	−0.001	0.004	0.011	0.229
ETH-BMGF	set03	2019/20	503	0.083	0.004	0.004	0.014	−0.164
ETH-BMGF	set04	2019/20	504	0.074	0.001	0.004	0.014	−0.524
ETH-BMGF	set05	2019/20	505	0.069	0.001	0.008	0.020	−0.376
ETH-BMGF	set06	2019/20	508	0.060	0.000	0.004	0.015	−0.222
ETH-BMGF	set07	2019/20	514	0.054	0.003	0.005	0.018	−0.369
ETH-BMGF	set08	2019/20	515	0.045	0.002	0.006	0.014	−0.584
ETH-BMGF	set09	2019/20	539	0.019	0.003	0.003	0.014	−1.789
ETH-BMGF	set10	2019/20	540	0.296	0.003	−0.030	0.014	−0.095
ETH-BMGF	set11	2019/20	541	1.605	0.002	0.033	0.017	−0.347
ETH-BMGF	set12	2019/20	108	1.270	−0.014	0.021	0.013	−0.233
ETH-BMGF	set13	2019/20	544	1.693	0.004	0.040	0.020	−0.463
ETH-BMGF	set14	2019/20	545	1.763	0.004	0.016	0.015	−0.160
ETH-BMGF	set15	2019/20	546	1.329	0.008	0.051	0.018	−1.549
ETH-BMGF	set16	2019/20	547	1.285	0.003	0.054	0.017	−0.995
ETH-BMGF	set17	2019/20	548	1.272	0.003	0.045	0.017	−0.047
ETH-BMGF	set18	2019/20	549	1.344	0.001	0.035	0.015	−0.637
ETH-BMGF	set19	2019/20	550	1.414	0.006	0.039	0.017	−1.375
ETH-BMGF	set20	2019/20	551	1.455	0.007	0.054	0.015	−2.979
MWI-BMGF	set01	2018/19	417	0.080	0.001	−0.027	0.010	−1.988
MWI-BMGF	set02	2018/19	418	0.082	0.004	−0.038	0.015	−1.573
MWI-BMGF	set03	2018/19	419	0.102	0.003	−0.042	0.013	−2.739
MWI-BMGF	set04	2018/19	420	0.099	0.002	−0.043	0.011	−2.119
MWI-BMGF	set05	2018/19	421	0.098	0.003	0.004	0.011	−0.767
MWI-BMGF	set06	2018/19	422	0.099	0.002	0.007	0.015	−0.964
MWI-BMGF	set07	2018/19	423	0.098	0.002	0.004	0.012	−0.813
MWI-BMGF	set08	2018/19	424	1.149	0.001	0.007	0.013	0.002
MWI-BMGF	set09	2018/19	425	1.129	0.001	0.006	0.012	0.386
MWI-BMGF	set10	2018/19	567	1.143	0.002	0.005	0.015	0.408
MWI-BMGF	set11	2018/19	833	0.146	0.004	0.006	0.011	−0.421
MWI-BMGF	set12	2018/19	834	0.135	0.001	0.006	0.012	−1.171
MWI-BMGF	set13	2018/19	835	0.124	0.003	0.005	0.010	−0.818
MWI-BMGF	set14	2018/19	836	0.126	0.001	0.005	0.012	−0.096
MWI-BMGF	set15	2018/19	837	0.108	0.001	0.008	0.012	−0.355
MWI-BMGF	set16	2018/19	838	0.101	−0.001	0.005	0.010	−2.510
MWI-BMGF	set17	2018/19	839	0.086	0.002	0.008	0.010	−1.034
MWI-BMGF	set18	2018/19	840	0.083	0.001	0.008	0.013	−1.317
MWI-BMGF	set19	2018/19	841	0.077	0.003	0.007	0.011	−1.867
MWI-BMGF	set20	2018/19	842	0.070	0.002	0.004	0.008	−2.867
MWI-BMGF	set21	2018/19	843	0.028	0.002	0.008	0.013	−2.117
MWI-BMGF	set22	2018/19	844	0.020	0.001	0.006	0.009	−1.084
MWI-BMGF	set23	2018/19	845	0.079	0.003	0.009	0.012	−0.902
MWI-BMGF	set24	2018/19	846	0.065	0.002	0.006	0.008	−1.679
MWI-BMGF	set25	2018/19	847	0.069	0.003	0.006	0.009	−1.548
MWI-BMGF	set26	2018/19	848	0.071	0.004	0.008	0.012	−0.181
MWI-BMGF	set27	2018/19	849	0.065	0.003	0.006	0.009	−0.663
MWI-BMGF	set28	2018/19	850	1.368	0.005	0.063	0.014	−1.880
MWI-BMGF	set29	2018/19	851	1.396	0.002	0.061	0.014	−1.339
MWI-BMGF	set30	2018/19	852	1.696	0.004	0.102	0.015	−1.221
MWI-BMGF	set31	2018/19	853	1.608	0.003	0.090	0.017	−1.596
MWI-BMGF	set32	2018/19	854	1.572	0.004	0.082	0.014	−0.259
MWI-BMGF	set33	2018/19	855	1.640	0.002	0.095	0.017	−0.892
MWI-BMGF	set34	2018/19	856	1.469	−0.001	0.057	0.012	−0.161
MWI-BMGF	set35	2018/19	857	1.643	0.000	0.060	0.014	0.036
MWI-BMGF	set36	2018/19	858	1.352	0.003	0.026	0.011	−0.426
MWI-BMGF	set37	2018/19	859	1.369	0.003	0.024	0.014	−1.147
MWI-BMGF	set38	2018/19	860	1.368	−0.001	0.018	0.009	0.231

**Online-only Table 8:** Limits of detection (3x standard deviation of blank samples expressed as mg kg^−1^) and number of blanks analysed for acid oxalate extraction of soil.

		Acid oxalate extractable (mg/kg)			
		Al	Fe	Mn	P
**LOD (Blank Stdev x3)**		62.6	76.7	4.0	102.8
	**N**	67	67	67	67

**Online-only Table 9:** Elemental concentrations (mg kg^−1^) in blanks used for acid oxalate extraction (average of two blanks in each batch). ETH = Ethiopia, MWI = Malawi, BMGF = Bill & Melinda Gates Foundation, GCRF = Global Challenges Research Fund.

Not blank corrected							
Average of two blanks in each batch				Acid oxalate extractable (mg/kg)			
Country_Project	ICP-Run	AY	AmmoniumOxalate_BatchID	Al	Fe	Mn	P
ETH-GCRF	set01	2018/19	115	−3.962	16.672	2.675	−32.632
ETH-GCRF	set02	2018/19	90	−16.224	7.018	2.313	−77.468
ETH-GCRF	set03	2018/19	91	−12.733	15.248	2.766	−73.689
ETH-GCRF	set04	2018/19	93	−67.859	−91.170	0.268	−79.233
ETH-GCRF	set05	2018/19	92	−99.969	######	−0.746	######
ETH-GCRF	set06	2018/19	94	5.772	46.880	4.362	−14.095
ETH-GCRF	set07	2018/19	95	3.578	28.385	4.032	−28.565
ETH-GCRF	set08	2018/19	96	44.810	21.382	3.864	−28.647
ETH-GCRF	set09	2018/19	97	1.021	40.777	3.759	−32.766
ETH-GCRF	set10	2018/19	98	−14.783	6.596	2.941	−70.546
ETH-GCRF	set11	2018/19	185	−6.781	17.807	1.589	−24.650
ETH-BMGF	set01	2019/20	459	−7.079	12.719	−0.338	−22.985
ETH-BMGF	set02	2019/20	460	−14.213	−1.570	−0.213	−35.631
ETH-BMGF	set03	2019/20	461	15.460	21.261	0.675	7.423
ETH-BMGF	set04	2019/20	462	11.489	19.309	1.408	13.252
ETH-BMGF	set05	2019/20	463	14.870	1.537	0.319	−42.261
ETH-BMGF	set06	2019/20	464	9.049	11.571	0.395	−29.199
ETH-BMGF	set07	2019/20	465	−16.377	0.140	−0.373	−53.873
ETH-BMGF	set08	2019/20	466	12.390	10.994	0.847	−25.896
ETH-BMGF	set09	2019/20	467	−3.961	−1.996	0.302	−1.963
ETH-BMGF	set10	2019/20	21	−30.853	−28.923	−1.130	######
ETH-BMGF	set11	2019/20	469	−12.948	−15.036	−0.834	−2.661
ETH-BMGF	set12	2019/20	470	5.189	15.314	0.600	−24.020
ETH-BMGF	set13	2019/20	471	5.460	9.759	0.765	−28.982
ETH-BMGF	set14	2019/20	472	23.884	9.362	0.395	1.259
ETH-BMGF	set15	2019/20	473	−22.209	10.717	0.505	−0.391
ETH-BMGF	set16	2019/20	474	−10.244	−15.706	−0.555	3.160
ETH-BMGF	set17	2019/20	475	2.926	−2.965	0.028	7.253
ETH-BMGF	set18	2019/20	476	−33.687	−25.367	−1.338	−12.905
ETH-BMGF	set19	2019/20	477	−40.889	−38.479	−1.796	−1.046
MWI-BMGF	set01	2018/19	557	−1.775	−5.686	−0.917	−81.788
MWI-BMGF	set02	2018/19	558	−16.327	−14.018	−1.437	−87.099
MWI-BMGF	set03	2018/19	559	−6.181	−10.267	−0.908	−83.765
MWI-BMGF	set04	2018/19	560	1.286	−12.244	−0.935	−74.734
MWI-BMGF	set05	2018/19	671	0.680	11.746	−0.367	−19.766
MWI-BMGF	set06	2018/19	672	4.575	1.383	−0.426	−23.412
MWI-BMGF	set07	2018/19	480	3.736	24.335	0.286	−0.171
MWI-BMGF	set08	2018/19	673	1.370	11.358	−0.361	−25.799
MWI-BMGF	set09	2018/19	481	−3.232	9.409	−0.160	−20.988
MWI-BMGF	set10	2018/19	482	−3.664	0.731	−0.362	−75.416
MWI-BMGF	set11	2018/19	750	18.848	11.313	0.035	−23.168
MWI-BMGF	set12	2018/19	752	−3.942	−10.913	−0.887	−73.402
MWI-BMGF	set13	2018/19	755	8.095	15.355	0.050	−9.032
MWI-BMGF	set14	2018/19	756	18.532	24.520	0.441	14.999
MWI-BMGF	set15	2018/19	757	14.186	36.533	0.394	23.905
MWI-BMGF	set16	2018/19	758	1.833	4.288	−0.298	−13.742
MWI-BMGF	set17	2018/19	759	−3.532	−5.550	−0.766	−52.100
MWI-BMGF	set18	2018/19	760	−7.247	−4.440	−0.932	−69.751
MWI-BMGF	set19	2018/19	761	7.560	18.542	0.142	−4.053
MWI-BMGF	set20	2018/19	762	1.161	24.189	−0.442	28.112
MWI-BMGF	set21	2018/19	763	−3.686	9.134	−0.482	−39.204
MWI-BMGF	set22	2018/19	764	−5.343	4.256	−0.236	−54.615
MWI-BMGF	set23	2018/19	765	−13.644	4.466	0.041	−52.053
MWI-BMGF	set24	2018/19	766	20.101	10.834	−0.659	−46.484
MWI-BMGF	set25	2018/19	767	4.439	30.153	0.721	2.634
MWI-BMGF	set26	2018/19	768	7.932	41.717	0.259	22.604
MWI-BMGF	set27	2018/19	769	9.387	15.576	0.013	−5.488
MWI-BMGF	set28	2018/19	770	18.831	20.848	−0.052	−7.726
MWI-BMGF	set29	2018/19	771	18.697	13.584	−0.578	−39.423
MWI-BMGF	set30	2018/19	772	33.898	21.929	−0.422	10.353
MWI-BMGF	set31	2018/19	773	10.931	12.673	0.002	−21.764
MWI-BMGF	set32	2018/19	774	−1.466	−0.810	−0.727	−74.628
MWI-BMGF	set33	2018/19	775	−20.385	−12.140	−0.763	−64.367
MWI-BMGF	set34	2018/19	776	−18.249	−11.051	−0.658	−67.557
MWI-BMGF	set35	2018/19	777	−9.591	−12.855	−0.584	−50.374
MWI-BMGF	set36	2018/19	778	−14.771	−20.994	−0.824	−68.938
MWI-BMGF	set37	2018/19	779	−12.457	−13.576	−0.695	−43.602

**Online-only Table 10:** Limits of detection (3x standard deviation of blank samples expressed as mg kg^-1^) and number of blanks analysed for Olsen-P analysis of soils.

	Olsen-P (mg/kg)
	PO_4_-P
LOD (Blank Stdev x3)	0.85
N	37

**Online-only Table 11:** Elemental concentrations (mg kg^−1^) in blanks used in for Olsen-P analysis of soils (average two blanks in each batch). ETH = Ethiopia, MWI = Malawi, BMGF = Bill & Melinda Gates Foundation, GCRF = Global Challenges Research Fund.

Not blank corrected					
Average of two blanks in each batch					Olsen-P (mg/kg)
Country_Project	ICP-Run	AY	OlsenP_BatchID	DoA	PO_4_-P
ETH-GCRF	set01	2018/19	732	17.7.18	0.27
ETH-GCRF	set02	2018/19	733	18.7.18	0.53
ETH-GCRF	set03	2018/19	734	19.7.18	0.20
ETH-GCRF	set04	2018/19	735	25.7.18	0.32
ETH-GCRF	set05	2018/19	736	26.7.18	0.70
ETH-BMGF	set01	2019/20	326	10.2.20	0.86
ETH-BMGF	set02	2019/20	327	11.2.20	0.31
ETH-BMGF	set03	2019/20	328	12.2.20	−0.06
ETH-BMGF	set04	2019/20	330	18.2.20	0.58
ETH-BMGF	set05	2019/20	331	19.2.20	0.04
ETH-BMGF	set06	2019/20	332	20.2.20	0.03
ETH-BMGF	set07	2019/20	333	3.3.20	0.61
ETH-BMGF	set08	2019/20	334	4.3.20	0.23
ETH-BMGF	set09	2019/20	335	5.3.20	0.22
ETH-BMGF	set10	2019/20	336	9.3.20	0.44
ETH-BMGF	set11	2019/20	337	10.3.20	0.65
MWI-BMGF	set01	2019/20	412	29.8.19	0.88
MWI-BMGF	set02	2019/20	413	18.8.19	0.14
MWI-BMGF	set03	2019/20	414	19.9.19	−0.10
MWI-BMGF	set04	2019/20	415	9.9.19	0.40
MWI-BMGF	set05	2019/20	416	28.9.19	0.12
MWI-BMGF	set06	2019/20	207	29.9.19	0.15
MWI-BMGF	set07	2019/20	208	11.11.19	0.15
MWI-BMGF	set08	2019/20	209	12.11.19	0.31
MWI-BMGF	set09	2019/20	210	13.11.19	0.80
MWI-BMGF	set10	2019/20	247	25.11.19	0.19
MWI-BMGF	set11	2019/20	248	26.11.19	0.00
MWI-BMGF	set12	2019/20	249	27.11.19	0.09
MWI-BMGF	set13	2019/20	250	28.11.19	0.60
MWI-BMGF	set14	2019/20	251	4.12.19	0.30
MWI-BMGF	set15	2019/20	252	5.12.19	0.05
MWI-BMGF	set16	2019/20	253	11.12.19	0.87
MWI-BMGF	set17	2019/20	254	12.12.19	0.60
MWI-BMGF	set18	2019/20	255	14.1.20	0.20
MWI-BMGF	set19	2019/20	256	15.1.20	0.20
MWI-BMGF	set20	2019/20	257	16.1.20	0.00
MWI-BMGF	set21	2019/20	258	1.11.19	0.60

**Online-only Table 12:** Limits of detection (3x standard deviation of blank samples) for the values of phosphorus buffering index (PBI) of soils.

	PBI
**LOD**^1^ **(Blank Stdev x3)**	2.22
**N**	70

^1^Assumes P_Olsen = 0

**Online-only Table 13:** Blank values (average two blanks in each batch) used in for PBI determination of soils. ETH = Ethiopia, MWI = Malawi, BMGF = Bill & Melinda Gates Foundation, GCRF = Global Challenges Research Fund.

Average of two blanks in each batch				
Country_Project	ICP_Run	AY	PBI_BatchID	PBI
**ETH-GCRF**	**set01**	**2018/19**	**17**	**−0.09**
ETH-GCRF	set02	2018/19	18	0.10
ETH-GCRF	set03	2018/19	19	−0.22
ETH-GCRF	set04	2018/19	20	−0.81
ETH-GCRF	set05	2018/19	21	0.37
ETH-GCRF	set06	2018/19	22	0.18
ETH-GCRF	set07	2018/19	23	0.44
ETH-GCRF	set08	2018/19	24	0.71
ETH-GCRF	set09	2018/19	25	1.49
ETH-GCRF	set10	2018/19	26	1.13
ETH-GCRF	set11	2018/19	183	−0.05
ETH-BMGF	set01	2019/20	351	−0.12
ETH-BMGF	set02	2019/20	352	1.32
ETH-BMGF	set03	2019/20	353	−0.16
ETH-BMGF	set04	2019/20	354	−0.97
ETH-BMGF	set05	2019/20	355	−0.14
ETH-BMGF	set06	2019/20	356	−0.10
ETH-BMGF	set07	2019/20	357	1.73
ETH-BMGF	set08	2019/20	358	−0.33
ETH-BMGF	set09	2019/20	359	−0.08
ETH-BMGF	set10	2019/20	360	−0.15
ETH-BMGF	set11	2019/20	361	−0.20
ETH-BMGF	set12	2019/20	362	0.53
ETH-BMGF	set13	2019/20	363	3.02
ETH-BMGF	set14	2019/20	364	1.82
ETH-BMGF	set15	2019/20	365	−0.17
ETH-BMGF	set16	2019/20	366	−0.68
ETH-BMGF	set17	2019/20	367	−0.04
ETH-BMGF	set18	2019/20	368	0.01
ETH-BMGF	set19	2019/20	369	−0.03
ETH-BMGF	set20	2019/20	370	0.12
ETH-BMGF	set21	2019/20	757	−0.20
ETH-BMGF	set22	2019/20	758	−0.04
MWI-BMGF	set01	2018/19	391	−0.19
MWI-BMGF	set02	2018/19	392	−0.08
MWI-BMGF	set03	2018/19	316	−0.12
MWI-BMGF	set04	2018/19	394	−0.34
MWI-BMGF	set05	2018/19	395	−0.31
MWI-BMGF	set06	2018/19	396	−0.10
MWI-BMGF	set07	2018/19	317	2.63
MWI-BMGF	set08	2018/19	398	0.36
MWI-BMGF	set09	2018/19	399	−0.37
MWI-BMGF	set10	2018/19	218	−0.25
MWI-BMGF	set11	2018/19	219	−0.24
MWI-BMGF	set12	2018/19	220	0.36
MWI-BMGF	set13	2018/19	221	−0.17
MWI-BMGF	set14	2018/19	222	−0.55
MWI-BMGF	set15	2018/19	223	−0.58
MWI-BMGF	set16	2018/19	224	−0.92
MWI-BMGF	set17	2018/19	225	0.00
MWI-BMGF	set18	2018/19	226	−0.21
MWI-BMGF	set19	2018/19	227	0.34
MWI-BMGF	set20	2018/19	228	0.48
MWI-BMGF	set21	2018/19	229	0.32
MWI-BMGF	set22	2018/19	230	−0.32
MWI-BMGF	set23	2018/19	231	−0.58
MWI-BMGF	set24	2018/19	232	−0.65
MWI-BMGF	set25	2018/19	324	−0.11
MWI-BMGF	set26	2018/19	234	0.19
MWI-BMGF	set27	2018/19	325	−0.73
MWI-BMGF	set28	2018/19	236	0.40
MWI-BMGF	set29	2018/19	237	0.77
MWI-BMGF	set30	2018/19	238	0.11
MWI-BMGF	set31	2018/19	239	0.28
MWI-BMGF	set32	2018/19	240	−0.11
MWI-BMGF	set33	2018/19	241	1.15
MWI-BMGF	set34	2018/19	242	−0.64
MWI-BMGF	set35	2018/19	243	−0.80
MWI-BMGF	set36	2018/19	311	0.12
MWI-BMGF	set37	2018/19	323	−0.07
