# Supplementary material for: Cereal grain mineral micronutrient and soil chemistry data from GeoNutrition surveys in Ethiopia and Malawi
Source: Sci Data. 2022 Jul 25;9:443. doi: 10.1038/s41597-022-01500-5 (PMC9314434; doi:10.1038/s41597-022-01500-5)
Supplement: Supplementary file 1 — Supplementary file 3 [file 41597_2022_1500_MOESM1_ESM.pdf]

Supplementary file 3. Cereal grain and soil sample field data collection questionnaires in Ethiopia and Malawi.

GeoNutrition crop and soil sample metadata, Ethiopia

Team ID

This is the identification number assigned to your team.

GID

This is the identification number from the original sampling site lists

Data recorder

Please input your full name (your name & father's name)

Locality name

Date and time of sampling

yyyy-mm-dd

hh:mm

GPS location

GPS coordinates can only be collected when outside.

latitude (x.y °)

longitude (x.y °)

altitude (m)

accuracy (m)

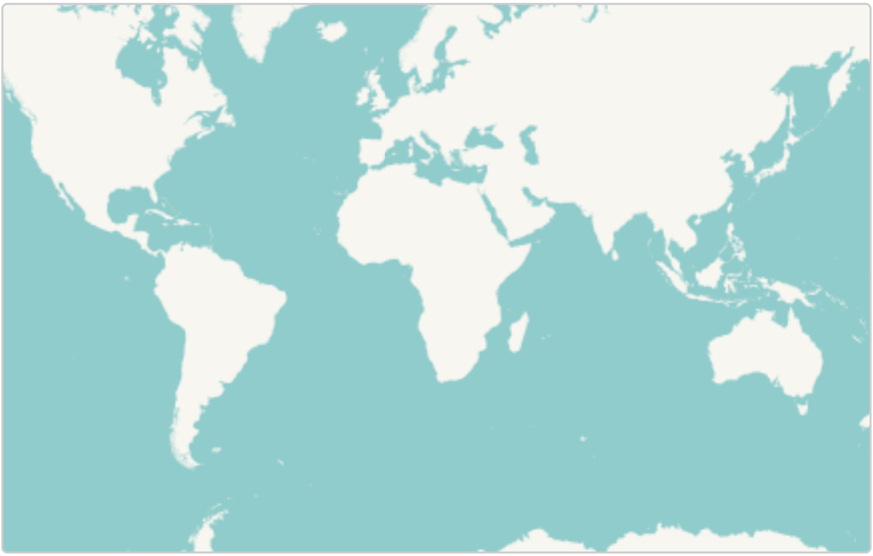

Site

- ◯ Main
- ◯ Close pair

Has the land been ploughed recently?

- ◯ Yes
- ◯ No

Crop sample

Sample ID

____

Source of grain sample

- ◯ Standing crop
- ◯ Field stack
- ◯ Store

Crop type

- ◯ Wheat
- ◯ Teff
- ◯ Maize
- ◯ Sorghum
- ◯ Finger millet
- ◯ Pearl millet
- ◯ Other

If crop type is "Other", please write the crop here.

____

Crop variety if known

____

Crop picture

Click here to upload file. (< 5MB)

Soil sample

Sample ID

____

Site picture

Click here to upload file. (< 5MB)

Sample bags picture

Click here to upload file. (< 5MB)

2018 GeoNutrition crop and soil sample metadata, Ethiopia

Team ID

This is the identification number assigned to your team.

TargetSiteID

This is the identification number for this field from the original sampling site lists.

Data recorder name

Please write down your initials. E.g., DG for Dawd Gashu

Locality name

What do the local community call this place? E.g., village/kebele name

GPS location

Tap on Start GeoPoint to record the latitude, longitude and altitude of this field. Wait until the tablet populates this data automatically. Make sure the accuracy is <10 m.

latitude (x.y °)

longitude (x.y °)

altitude (m)

accuracy (m)

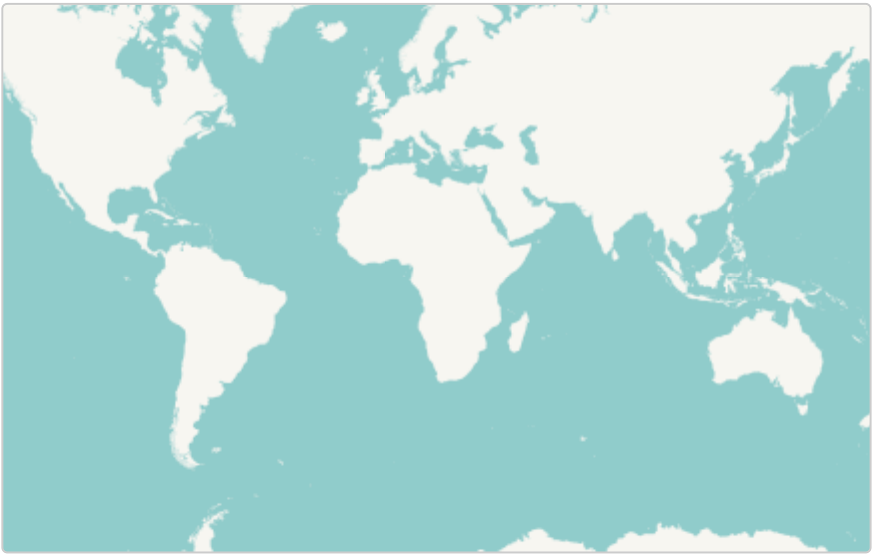

Site

Is this site labelled as main or close pair on your sample list for your team? If it is a close pair, please select "Close Pair" for both sample sites

- ◯ Main
- ◯ Close pair

Has the land been tilled recently (since harvest)?

- ◯ Yes
- ◯ No
- ◯ Minimum tillage is practiced
- ◯ Unknown

Crop sample data

CropSampleID

Tap on Get Barcode to scan the QR-Code label on the cereal grain bag from this field using the tablet camera. Make sure that there is no other label in the sight of the tablet camera. After scan, check the sample ID recorded is what you see on the label of the grain sample bag from this field.

____

Source of grain sample

- ◯ Standing crop
- ◯ Field stack
- ◯ Store

Crop type

- ◯ Teff
- ◯ Wheat
- ◯ Triticale
- ◯ Barley
- ◯ Maize
- ◯ Sorghum
- ◯ Pearl millet
- ◯ Finger millet
- ◯ Rice
- ◯ Other cereal grain (specify in the next question)

If other cereal grain was selected, please specify

____

Crop variety if known

____

Was this an inter-cropped field?

- ◯ Yes
- ◯ No
- ◯ Unknown

If yes, what was inter-cropped?

- [ ] Legume-grain
- [ ] Ethiopian kale-grain
- [ ] Other (specify)
- [ ] Unknown

If other was selected, please specify

____

What was the grain yield from this field?

Please select the unit for the cereal grain yield in the next question. If the yield is unknown, please enter 0 and select the unit as "Unknown"

____

What is the unit for the grain yield?

- ○ 50 kg sacks of clean grain per hectare
- ○ Quintal per hectare
- ○ Other (specify)
- ○ Unknown

If other was selected, please specify.

____

Crop picture

Take the picture of the standing crop/ stack using the tablet camera.

Click here to upload file. (< 5MB)

Soil sample and field data

SoilSampleID

Tap on Get Barcode to scan the QR-Code label on the soil bag from this field using the tablet camera. Make sure that there is no other label in the sight of the tablet camera. After scan, check the sample ID recorded is what you see on the label of the soil sample bag from this field.

____

How was the soil sample taken?

- ○ Using auger
- ○ Using trowel

What is the colour of the soil?

- ◯ Black
- ◯ Brown
- ◯ Dark brown
- ◯ Light brown
- ◯ Sandy yellow
- ◯ Red
- ◯ Other (specify in the next question)

If "Other" was selected as the soil colour, please describe it here.

____

Was fertiliser applied to this field in this cropping season?

- ◯ Yes
- ◯ No
- ◯ Unknown

If yes, which one (s)?

- ☐ NPK
- ☐ NPS
- ☐ NPK (Boron and zinc blended)
- ☐ NPS (Boron and zinc blended)
- ☐ Urea
- ☐ Urea (Boron and Zinc blended)
- ☐ Diammonium phosphate
- ☐ Ammonium nitrate
- ☐ Triple super phosphate
- ☐ Other (specify in the next question)
- ☐ Unknown

If other fertilizer was selected, please specify here

____

What was the fertilizer application rate? (kg per hectare)

____

Was lime applied to this field in this cropping season?

- ◯ Yes
- ◯ No
- ◯ Unknown

If yes, what type of lime? E.g., dolomite, chalk, etc

What was the lime application rate? (kg per hectare)

Were manure or organic residues applied to this field in this cropping season?

- ◯ Yes
- ◯ No
- ◯ Unknown

If yes, what type

- ☐ Farmyard manure
- ☐ Compost
- ☐ Crop residue left on field
- ☐ Crop residue burned on the field
- ☐ Other (specify in the next question)
- ☐ Unknown

If other was selected above, please specify

Site picture

Take the picture of the site with some easily identifiable landmarks.

Click here to upload file. (< 5MB)

Sample bags picture

Take the picture of the grain and soil bags with the labels facing the camera.

Click here to upload file. (< 5MB)

GeoNutrition crop and soil sample metadata, Malawi

Team ID

This is the identification number assigned to your team.

____

GID

This is the identification number for this field from the original sampling site lists.

____

Data recorder

Please write down your initials.

____

Locality name

What do the local community call this place?

____

Date and time of sampling

No need for manual input. Please swipe left to continue to the next question.

yyyy-mm-dd ______________ hh:mm ______________

GPS location

Tap on Record Location to record the latitude, longitude and altitude of this field. Wait until the tablet populates this data automatically. Make sure the accuracy is <10 m.

____

latitude (x.y °)

____

longitude (x.y °)

____

altitude (m)

____

accuracy (m)

____

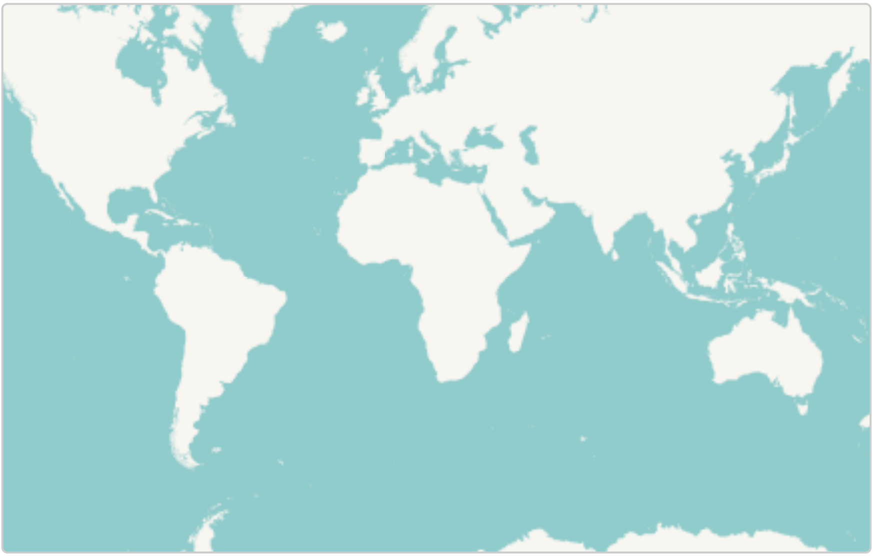

Site

Is this site labelled as main or close pair on your sample list for your team?

- ◯ Main
- ◯ Close pair

Has the land been tilled recently (since harvest)?

- ◯ Yes
- ◯ Not yet
- ◯ Minimum tillage is practiced
- ◯ Unknown

Crop sample data

CropSample ID

Tap on Get Barcode to scan the QR-Code label on the cereal grain bag from this field using the tablet camera. Make sure that there is no other label in the sight of the tablet camera. After scan, check the sample ID recorded is what you see on the label of the grain sample bag from this field.

____

Source of grain sample

- ◯ Standing crop
- ◯ Field stack
- ◯ Store

Crop type

- ◯ Maize
- ◯ Sorghum
- ◯ Finger millet
- ◯ Pearl millet
- ◯ Rice
- ◯ Other cereal grain (specify in the next question)

If other cereal grain was selected, please specify

____

Crop variety if known

____

Was this an inter-cropped field?

- ◯ Yes
- ◯ No
- ◯ Unknown

If yes, what was inter-cropped?

- [ ] Legume-grain
- [ ] Cassava-grain
- [ ] Legume-cassava-grain
- [ ] Other (specify)
- [ ] Unknown

If other was selected, please specify

___

What was the grain yield from this field?

Please select the unit for the cereal grain yield in the next question. If the yield is unknown, please enter 0 and select the unit as "Unknown"

___

What is the unit for the yield?

- ( ) 50kg sacks of clean grain per acre
- ( ) Ox-carts of maize cobs with husks per acre
- ( ) Ox-carts of dehusked maize cobs per acre
- ( ) Unknown

Crop picture

Take the picture of the standing crop/ stack using the tablet camera.

Click here to upload file. (< 5MB)

Soil sample and field data

SoilSample ID

Tap on Get Barcode to scan the QR-Code label on the soil bag from this field using the tablet camera. Make sure that there is no other label in the sight of the tablet camera. After scan, check the sample ID recorded is what you see on the label of the soil sample bag from this field.

___

What is the colour of the soil?

- ( ) Black
- ( ) Brown
- ( ) Dark brown
- ( ) Light brown
- ( ) Sandy
- ( ) Red
- ( ) Other (specify in the next question)

If "Other" was selected as the soil colour, please describe it here.

___

Was fertiliser or lime applied to this field in this cropping season?

- ○ Yes
- ○ No
- ○ Unknown

If yes, which one (s)?

- ☐ Urea
- ☐ Diammonium phosphate
- ☐ Ammonium nitrate
- ☐ NPK (23:21:0 + 4S) basal dressing
- ☐ NPK with Zn (23:10:5 + 1%Zn + 6%S)
- ☐ Triple super phosphate
- ☐ Lime
- ☐ Other (specify in the next question)
- ☐ Unknown

If other fertiliser/lime was selected, please specify here

What was the rate of application? (number of 50 kg bags per acre)

Were manure or organic residues applied to this field in this cropping season?

- ○ Yes
- ○ No
- ○ Unknown

If yes, what type

- ☐ Farmyard manure
- ☐ Compost
- ☐ Crop residue left on field
- ☐ Crop residue burned on the field
- ☐ Other (specify in the next question)
- ☐ Unknown

If other was selected above, please specify

Site picture

Take the picture of the site with some easily identifiable landmarks.

Click here to upload file. (< 5MB)

Sample bags picture

Take the picture of the grain and soil bags with the labels facing the camera.

Click here to upload file. (< 5MB)
