# Supplementary material for: Cereal grain mineral micronutrient and soil chemistry data from GeoNutrition surveys in Ethiopia and Malawi
Source: Sci Data. 2022 Jul 25;9:443. doi: 10.1038/s41597-022-01500-5 (PMC9314434; doi:10.1038/s41597-022-01500-5)
Supplement: Supplementary file 13 — MWI_Crop_LOD_ByICPRun [file 41597_2022_1500_MOESM13_ESM.pdf]

Supplementary file 1. Ethical approvals, information sheets, and farmers' consent form in English, Amharic, Afaan Oromoo and Tigrigna for the Ethiopian GeoNutrition data collection.

Appendix A: Information Sheet for Participants (የጥናት ፈቃድ ፎርም)

የፕሮጀክቱስም፡ጂኦኒውትሪሽን

ርዕስ፡የአፈርእናእህልማዕድናትይዘትበኢትዮጲያ

Invitation: Good morning/ good afternoon. My name is…………I am a member of the Geonutrition study team by Addis Ababa University and the University of Notthigham, UK.You are being invited to be involved in a research study; before you decide whether you want to take part, it is important for you to understand why the research is being done and what your participation will involve. Please read the following information carefully and discuss it with other people if you wish. Please contact me if anything is unclear or if you would like more information. Take time to decide whether or not you wish to take part.

እንደምን አደሩ /ዋሉ? ስሜ__________ይባላል፡፡ የአዲስ አበባ ዩኒቨርስቲ የምግብ ሳይንሰና ኒውትሪሽን ማዕከል እንግሊዝ ሀገር ከሚገኘው ኖቲንግሃም ዩኒቨርስቲ ጋር በመተባበር ከሚሰራው የጂኦኒውትሪሽን ፕሮጀክት የጥናት ቡድን አባል ነኝ፡፡ የእርሻ መሬት ስላሎት ወይንም እህል ስለሚያመርቱ በዚህ ጥናት እንዲሳተፉ ተጋብዘዋል፡፡ በጥናቱ ለመሳተፍ ከመወሰኖ እና ፈቃዶን ከመስጠቶ በፊት ስለጥናቱ ዓላማ እና ከእርሶ ተሳትፎ የምንጠብቀውን ልግለፅሎት፡፡ እዚህ የጥናት ፈቃድ ቅፅ ውስጥ ያሉትን መረጃዎች አንብሎታለሁ፡፡ ግልፅ ያልሆኑ ነገሮች ያልኩ እንደሆነ ያለ ምንም ማመንታ ሊጠይቁኝ ይችላሉ፡፡ እኔም መልስ እሰጣለሁ፡፡ እዚህ የጥናት ፈቃድ ቅፅ ውስጥ ያሉትን መረጃዎች ከሌሎች ሰዎች ጋር ሊወያዩባቸው ይችላሉ፡፡ ተሳትፎት ሙሉ በሙሉ በእርሶ ፈቃደኝነት ላይ የተመሰረተ ነው፡፡ ለመሳተፍ ካልተስማሙ ምንም አይነት ቅጣት አይደርስቦትም፡፡

What is the purpose of this study?The aim of this study is to understand how soil quality affects the nutritional value of cereal grains growing in those fields.

የጥናቱ ዓላማ ምንድን ነው?

የዚህ ጥናት ዓላማ የእህል የማዕድን ይዞታ በአፈሩ አይነት /ፀባይ እንዴት እንደሚወሰን ለመረዳት ነው፡፡

Why I have been chosen? You are randomly selected because you grow cereal grain within Malawi/Ethiopia. There is no specific reason other than this.

እኔ ለምን ተመረጥኩ?
የእርሻ መሬት ስላሎት ወይንም እህል ስለሚያመርቱ በዚህጥናት እንዲሳተፉ ተመርጠዋል፡፡ ነገር ግን ከሌሎች አርሶ አደሮች በተለየ የተመረጡት ታቅዶ እና በተለየ ምክነያት ሳይሆን በአጋጣሚ ነው፡፡

What will participation involve?You will be asked questions about your field, soils, crops, and how you manage these. Your responses will be written down on a tablet (please show the tablet for the participant). We will then ask to take a small sample of soil and cereal grain from your field (or crop store).

በጥናቱ ብሳተፍ ከእኔ ምን ይጠበቃል?

በጥናቱ ቢሳተፉ ስለ እርሻ መሬቶ የአፈር አይነት ወይም ፀባይ እና ስለሚያመርቱት የእህል አይነት እጠይቆታለሁ፡፡ መልሶ በያዝኩት ታብሌት (ታብሌቱን ለተሳታፊው አሳይ) ላይ እመዘግባለሁ፡፡በተጨማሪም ከእርሻ መሬቶ ወይንም ከጎተራዎ ትንሽ የአፈር እና የእህል ናሙና እወስዳለሁ፡፡

What if I decide that I don’t want to take part?You are free to decide that you don’t want to take part in the study and can:

1. Refuse to answer any questions that you don’t want to
2. Decide to stop the interview at any time
3. Remove your consent for the data collected to be used.

Reference number:Ethical Approval Number BIO-1819-001

በጥናቱ ለመሳተፍ ፈቃደኛ ባልሆንስ?

ተሳትፎት ሙሉ በሙሉ በእርሶ ፈቃደኝነት ላይ የተመሰረተነው፡፡ስለሆነም

1. መመለስ ያልፈለጉትን ጥያቄ መተው ይችላሉ
2. ቃለ መጠይቁን በማንኛውም ጊዜ ሊያቋርጡ ይችላሉ
3. ለመሳተፍ ፈቃደኛ ከሆኑ እና ቃለ መጠይቁን ካደረግን በኋላ እንኳን ፈቃደኝነቶን ማጠፍ ይችላሉ

Will I be paid for my time?There is no payment for taking part in this questionnaire.

በጥናቱ ብሳተፍ ይከፈለኛል?

በጥናቱ በመሳተፎ ምንም አይነት የገንዘብ ክፍያ አያገኙም፡፡

Will I be anonymous, and who will know my identity? If you agree to take part in an interview, a Participant Number will be generated for you, and that's the only thing that will be used to identify you. Your identity will only be known by the interviewer, and will not be found in any record. Hard copy and electronic data will be stored on the University of Nottingham's computer network: thiswill be deleted after 7 years, or if you withdraw your consent (whichever is sooner).

በጥናቱ ብሳተፍ የሰጠሁትን መረጃ ማን ሊያውቅ ይችላል?

በጥናቱ ለመሳተፍ ከተስማሙ የሰጡት ቃለ መጠይቅም ሆነ የአፈር እና የእህል ናሙና በስሞ ሳይሆን በቁጥሮች ይወከላል፡፡ እርሶ የሰጡትን መረጃ የሚያውቀው ቃለመጠይቁን ያደረገው ሰው ብቻ ነው፡፡ ነገር ግን በማንኛውም ሰነድ ላይ አይገኝም፡፡ የወረቀት ሰነዶችም ሆኔ የኤሌክትሮኒክስ ቅጂዎች በአዲስ አበባ ዩኒቨርስቲ እና እንግሊዝ ሀገር በሚገኘው ኖቲንግሃም ዩኒቨርስቲ ብቻ ይቀመጣሉ፡፡ ጥናቱ ከተጠናቀቀ በኋላ ወይንም በጥናቱ እንዲሳተፉ ፈቃደኝነቶን ካጠፉ ቅጂዎቹ ሙሉ በሙሉ እንዲጠፉ ወይንም እንዲወድሙ ይደረጋል፡፡

Who shall I contact with any questions?Please contact the Principal Investigators, Redacted (Addis Ababa University, Center for Food Science and Nutrition; Redacted ስልክ Redacted); Redacted (University of Nottingham, UK; Redacted, Redacted. If you wish to ask questions or make a complaint you may also contact either Research Ethics Officer: Redacted (Office of the Associate Dean, Addis Ababa University; Redacted; Redacted) or Redacted (University of Nottingham, UK; or Redacted).

ጥያቄ ካለኝ የጥናቱ ተጠሪዎች እነማን ናቸው?

ምንም አይነት ጥያቄ ካለዎት በማንኛውም ሰዓት መጠየቅ ይችላሉ፡፡ ስለዚህ ጥናት ተጨማሪ ጥያቄዎች ካለዎት ዶ/ር ዳውድ ጋሹ፤ አዲስ አበባ ዩኒቨርስቲ የምግብ ሳይንሰና ኒውትሪሽን ማዕከል (Redacted ; ስልክ Redacted) መጠየቅይችላሉ፡፡ ወይንም Redacted ፤ ኖቲንግሃም ዩኒቨርስቲ Redacted ስልክ Redacted) መጠየቅ ይችላሉ፡፡ በተጨማሪም ስለ ምርምሩ ጥያቄ ወይንም ቅሬታ ካለዎት የአዲስ አበባ ዩኒቨርስቲ ተፈጥሮ ሳይንስ ኮሌጅ የምርምር ዳይሬክተር የሆኑትን Redacted (ስልክ Redacted ወይንም Redacted (ኖቲንግሃም ዩኒቨርስቲ ፣ እንግሊዝ Redacted መጠየቅ ይችላሉ፡፡

Reference number:Ethical Approval Number BIO-1819-001

Appendix B: Consent Form / Fomuyachilolezo

You have been invited to take part in a research project. If you like to participate, please sign or give your left thumb impression at the space indicated below.

በዚህ ጥናት እንዲሳተፉ ተጋብዘዋል፡፡ በጥናቱ ለመሳተፍ ከተሰማሙ ከታች ባለው ክፍት ቦታ ፊርማዎትን ወይን የግራ አውራ ጣቶን አሻራ ያስቀምጡ፡፡

By completing this form you are consenting to take part in this research project; you can withdraw your consent at any point. To withdraw your consent, please either mention that to the Interviewer during the interview or contact Redacted (Addis Ababa University Center for Food Science and Nutrition; Redacted and/or (University of Nottingham, UK; Redacted Redacted).If you wish to ask questions or make a complaint you may also contact either Research Ethics Officer: Redacted (Office of the Associate Dean, Addis Ababa University; Redacted) or Redacted (University of Nottingham, UK; or Redacted).

ይህንን የጥናት ፈቃድ ቅፅ ስለፈረሙ በጥናቱ ለመሳተፍ እንደተስማሙ እንወስደዋለን፡፡ ነገርግን በማንኛውም ሰአት ተሳትፎዎን ማቋረጥ ይችላሉ፡፡ተሳትፎዎትን ማቆም ሲሹ ቃለመተየቅ አድራጊዉን ወይም Redacted (አዲስ አባባ ዩኒቨርሲቲ፣ የምግብ ሳይንሰና ኒውትሪሽን ማዕከል Redacted) ወይም Redacted ፤ኖቲንግሃም ዩኒቨርስቲ (Redacted) ማሳወቅ ይችላሉ፡፡በተጨማሪም ስለ ምርምሩ ጥያቄ ወይንም ቅሬታ ካለዎት የአዲስ አበባ ዩኒቨርስቲ ተፈጥሮ ሳይንስ ኮሌጅ የምርምር ዳይሬክተር የሆኑትን Redacted (ስልክ Redacted ; e-mail: Redacted) ወይንም Redacted (ኖቲንግሃም ዩኒቨርስቲ ፣ እንግሊዝ; Redacted መጠየቅ ይችላሉ፡፡

Before signing this form, please read the following statements and indicate that you agree with them by initialling "√"next to them.

ጥናት ፈቀድ ቅፅ ላይ ከመፈረሞ በፊት የሚከተሉትን ነጥቦች በመገንዘብ መስማማቶን ለመግለፅ ከእያንዳንዱ ነጥቦች ጎንየ "✓" ምልክት ያድርጉ፡፡

GID#	Tick here<br>እዚህ ምልክት ያድርጉ(✓)
I have been issued with a Participant Information Sheet<br><br>ስለ ጥናት ፈቃድ ቅፁ ገለፃ ተደርጎልኛል፡፡	
I have been informed what the purpose of this research is, and the nature of the study<br><br>ስለ ጥናቱ ዓላማ እና አጠቃላይ ሁኔታ ገለፃ ተደርጎልኛል፡፡	
I have been informed how the data that are collected within the research will be handled and stored.	

በጥናቱ የሚሰበሰቡት መረጃዎች እንዴት እንደሚያዙ እና እንደሚቀመጡ ገለፃ ተደርጎልኛል፡፡	
I have been informed that I can remove my consent at any time either during, or after the interview, and that withdrawal of consent will not harm me in any way.<br><br>ቃለ መጠይቁ እየተካሄደ ባለበት ግዜም ሆነ መረጃውን ከተሰበሰበ በኋላ የሰጠሁትን በጥናቱ የመሳተፍ ፈቃድ ማጠፍ እንደምችል ያንን ተከትሎ ምንም አይነት ነገር እንደማይደርስብኝ በግልፅ ተነግሮኛል፡፡	
I have been informed that the interview will be written down on tablet<br><br>ለቃለ መጠይቁ የሰጠኋቸው መልሶች በታብሌቱ ተፅፈው እንደሚቀመጡ ተነግሮኛል፡፡	
I have been informed that my anonymised quotes may be used within the reporting of this research.<br><br>ለቃለ መጠይቁ የሰጠኋቸው መልሶች ስሜ ሳይጠቀስ በጥናቱ ሪፖርት ላይ ሊጠቀሱ እንደሚችሉ ተነግሮኛል፡፡	
I agree to take part in this study<br><br>በዚህ ጥናት ለመሳተፍ ተስማምቻለሁ፡፡	

Signed by______________________________ Date____________

ፊርማ___________________________________ ቀን_______________

Consent received by__________________________ Date________________

የቃለ መጠይቁ አድራጊ ፊርማ______________________ቀን________________

Appendix A: Information Sheet for Participants (**Guca Eeyyama qorannoo**

Maqaa projektii: Gi'O Qornyaata

Mata dureen: Qabiyyee biyyeetiifii albuudootaa midhaan Itiyoophiyaati

Invitation: Good morning/ good afternoon. My name is…………I am a member of the Geonutrition study team by Addis Ababa University and the University of Notthigham, UK.You are being invited to be involved in a research study; before you decide whether you want to take part, it is important for you to understand why the research is being done and what your participation will involve. Please read the following information carefully and discuss it with other people if you wish. Please contact me if anything is unclear or if you would like more information. Take time to decide whether or not you wish to take part.

Akkam bultan/Ooltan? Maqaankoo………………………jedhama. An Yunivarsiitii Addis Ababaatti, biyya Ingiliziitti kan argamuYuunivarsitii Notinghami wajjin walii galuun kan hojjatu miseensa garee qorannoo projektii Qornyaataati.

Waan Lafa qonnaa qabdaniif yookan midhaan omishtaniif qorannoo kanaratti afferamtanii jirtu. Osoo qorannoo kanaratti hirmaachuuf hin mirkaneesin fi eyyama hin kennin duratti waa'ee kaayyoo qorannootiifi hirmaannaa isinirraa eegamu isiniif haa ibsu.Odeeffanoowwan Uunkaa keessatti argaman dubbisaa namoota biroo wajjinis mari'achuu ni dandeessu.Waan isiniif ifa hin taane yoon dubbadhe shakkii tokko malee nagaafachuu dandeechu. Anis deebii isiniifan kenna. Yeroo fudhachuudhaan hirmaachuudhaaf fedhii qabaachuu fi dhabuu keessan murteessaa.

What is the purpose of this study? The aim of this study is to understand how soil quality affects the nutritional value of cereal grains growing in the fields.

Kaayyoon Qorannoo maalidha? Kaayyoon qorannoo kanaa qulqullinni biyyee midhaanni irratti guddatu dhiibbaa hammamii akka qabiyyee dhangaa midhaan callaa sanaarratti fidu hubachuufi.

Why I have been chosen?You are randomly selected because you grow cereal grain within Malawi/Ethiopia. There is no specific reason other than this.

Ani maalifan filatamee?

Sababa midhaan oomishtaniif qorannoo kanarratti akka hirmaattaniif filatamtanii jirtu. Isin qonnaan bultoota biraatirraa waanti adda taatanii filatamtaniif itti yaadamee yookan sababa biratin osoo hin taane akkuma carraati.

What will participation involve?You will be asked questions about your field, soils, crops,

and how you manage these. Your responses will be written down on a tablet. We will then ask to take a small sample of soil and cereal grain from your field (or crop store).

Qu'anooratti yoon hirmaadhee anaraa maaltu eegama?

Qu'anoo kana irrati yoo hirmaattan waa'ee lafa qonnaa keessanii, biyyee fi midhaanii isin gaafadha, deebii keessan, tabileetii irratti isiniif galmeessa, dabalatanis biyyee lafa qonnaa irraa fi midhaan irraa samuuda xiqqoo nan fudhadha.

What if I decide that I don't want to take part?You are free to decide that you don't want to take part in the study and can:

1. Refuse to answer any questions that you don't want to
2. Decide to stop the interview at any time
3. Remove your consent for the data collected to be used.

Qorannoo irratti hirmaachuuf eeyyamamaa yoon ta'uu badheehoo?

Hirmaannan guutumaan guututi heeyyama keessanirratti kan hundaa'ee dha, kanaafuu

1. Gaaffii deebisuu hin barbaane dhiisuu ni dandeessu.

2.Gaaafii afaani yeroo barbaaddan addaan kutuu ni dandeessu.

3.Hirmaachuuf eyyamamaa taataaniifi gaafii afaani erga raawwannee booda eeyyama keessan jijjiiruu ni dandeessu.

Will I be paid for my time?There is no payment for taking part in this questionnaire.

Qorannoorrati yoon hirmaadhe naaf kafalamaa?

Qu'anooratti yoo hirmaattan kaffaltiin qarshii homaatu isiniif hin kaffalamu.

Will I be anonymous, and who will know my identity? If you agree to take part in an interview, a participant number will be generated for you, and that's the only thing that will be used to identify you. Your identity will only be known by the interviewer, and will not be found in any record. Hard copy and electronic data will be stored on the University of Nottingham's computer network: thiswill be deleted after 7 years, or if you withdraw your consent (whichever is sooner).

Eenyuun akkan ta'e akka hinbaramne hintasifamaa? Eenyuumaakoo eenyutuu gara fulduraatti beekuu danda'aa?

Reference number:Ethical Approval Number BIO-1819-001

Qu'anooratti hirmaachuuf erga waliigalamee fi gaaffi afaanii erga kennitanii samuuda biyyee fi midhaanii kennitan maqaa keessaniin osoo hin ta'in lakkoofsaan bakka bu'a. Odeeffannoo isin kennitan kan beeku danda'u nama gaaffii afaanii isiniif godhe duwwaadha. Garuu uunkaa kamirrattuu hin argamu. Uunkan waraqatootas haata'u waraabbiin elektrooniksii kan taa'u biyya Ingiliziitti kanargamu Yunivarsiiti Notighaam duwwaatti. Qorannoon erga raawwatee booda yookan qu'anoorratti hirmaachuuf eeyama keesan yoo jijjirtan warraabbiiwan guutumaan guutuutti akka baduufi barbadaa'u ni godhama.

Who shall I contact with any questions? Please contact the Principal Investigators, Dr. Dawd Gashu (Addis Ababa University, Center for Food Science and Nutrition; Redacted ; ስልክ Redacted); Redacted (University of Nottingham, UK; Redacted , Redacted . If you wish to ask questions or make a complaint you may also contact either Research Ethics Officer: (Office of the Associate Dean, Addis Ababa University; Redacted) or Redacted (University of Nottingham, UK; or Redacted

Gaafi yoon qabaadhe itigaafatamtooni qu'anoo enyufa'a?

Gaafi barbaadan yoo qabatan yeroo barbaadan gafachuu nidandeesu. Kanaafu waa'ee qorannoo kana gaafi dabalataa yoo qabatan YKN hojjetaa Univarsittii Addis Ababaa kan ta'an Redacted quunnam (Redacted ; Lak. Bil. Redacted) gafachuu dandeesu. Yookan hojjetaa Univarsitii Nottingham (Biya Inglizii) kan ta'an Redacted (Redacted ; Lak. Bil. Redacted) gafachuu dandeesu. Yoo gaaffii yookaan komee qabaattan ogeessa naamusa qorannoo kan ta'an Redacted (Biiroo deggeraa diinii yuunivarsitii Addis Ababaa ; Redacted) Yookaan Redacted (yuunivarsitii Notiingam , Ingilizii; or Redacted ; Redacted) quunnamuu dandeessu.

Reference number:Ethical Approval Number BIO-1819-001

Appendix B: Consent Form / Fomuyachilolezo

You have been invited to take part in a research project. If you like to participate, please sign or give your left thumb impression at the space indicated below.

Lafa qonnaa wan qabdaniif yookan midhaan waan omishtaniif qu'anoo kanarrat aka hirmaatan afeeramtaniirtu. Qu'anno kanarrat hirmaachuf yoo waliigalu baka du'waa armaan gadiit jiruurrat malatoo keesan yoo kan ashaara quba abuduu harkaa bitaa keesan kaa'a.

By completing this form you are consenting to take part in this research project; you can withdraw your consent at any point. To withdraw your consent, please either mention that to the Interviewer during the interview or contact Redacted , Addis Ababa University, Center Fof Food Science and Nutrition, tel: 911745153 and/or Redacted (University of Nottingham, UK). If you wish to ask questions or make a complaint you may also contact either Research Ethics Officer: Redacted (Office of the Associate Dean, Addis Ababa University; Redacted) or Redacted (University of Nottingham, UK; or Redacted

Uunkaa Eeyama qorannoo kanaa waan malateesitaniif hirmaachuuf akka waliigaleti fudhana. Garuu yerroo barbaadan hirmaana adaan kutuu nidandeesu. Yoo addan kutu barbaadde, nama gaaffii waa'ee qorannoo sigaafatu YKN hojjetaa Univarsittii Finfinnee kan ta'an Redacted quunnam (Lak. Bil. Redacted) YKN hojjetaa Univarsitii Nottingham (Biya Inglizii) kan ta'an Redacted quunnamaa. Yoo gaaffii yookaan komee qabaattan ogeessa naamusa qorannoo kan ta'an Redacted (Biiroo Diinii yuunivarsitii Addis Ababaa ; Redacted) Yookaan Redacted (yuunivarsitii Notiingam , UK; or Redacted) quunnamuu dandeessu.

Before signing this form, please read the following statements and indicate that you agree with them by initialling next to them.

Eeyyama Uunkaa qorannoo kanaratti mallatteessuun duratti qabxiiwwaan armaan gadiirratti waliigaluu ibsuuf qabxiiwwan hunda cinatti fakkii ka'aa.

GID#	Tick here (x)
I have been issued with a Participant Information Sheet<br><br>Waa’ee eyyama Uunkaa qorannoo ibsi naaf godhameera.	
I have been informed what the purpose of this research is, and the nature of the study<br><br>Waa’ee kaayyoo qorannoo kanaafi haalli isaa bal’inaan naaf ibsamee jira.	
I have been informed how the data that are collected within the research will be handled and stored.<br><br>Odeefannoowwan qorannoo kanaaf funaanaman akamitti akka qabamaniifi ta’aan ibsi naaf godhameera.	
I have been informed that I can remove my consent at any time either during, or after the interview, and that withdrawal of consent will not harm me in any way.<br><br>Gaaffiin afaanii bakka rawwatetti erga odeefannoon guurameen boodas heeyyama hirmaanaa jijjiiruu akka danda’uuf hoomtuu akka narra hin geenye ifaan natti himamee jira.	
I have been informed that the interview will be written down on tablet<br><br>Deebiiwwan gaaffi afaanii itti kenne Tabileetirratti bareeffamee akka taa’u natti himameera.	
I have been informed that my anonymised quotes may be used within the reporting of this research.<br><br>Deebiiwwan gaaffii afaaniif kenne maqaakoo osoo hin ibsin unkaa qorannoo rippoortaraa irratti akka ibsamu nati himaameera.	
I agree to take part in this study<br><br>Qorannoo kanaratti hirmaachuf walii galeen jira.	

Reference number:Ethical Approval Number BIO-1819-001

Signed by……………………………………………Date…………………

Mallatoo……………………………………………Guyyaa…………………

Consent received by________________________________Date______________

Mallatoo gaafi afaani kangodhee___________________Guyyaa___________

Appendix A: Information Sheet for Participants/ Tsamba la unthengawachidziwitsokwaotenganawombali: "GeoNutrition (Work Package 1): Sampling soils and crops in Malawi / Kutenga ma sampulo a dothindimbewuku Malawi"

Invitation: Good morning/ good afternoon. My name is…………I am a member of the Geonutrition study team by Addis Ababa University and the University of Notthigham, UK.You are being invited to be involved in a research study; before you decide whether you want to take part, it is important for you to understand why the research is being done and what your participation will involve. Please read the following information carefully and discuss it with other people if you wish. Please contact me if anything is unclear or if you would like more information. Take time to decide whether or not you wish to take part.

ከመይ ወዒሎም/ሓዲሮም? ሽመይ..ይብሃል። ናይ ኣዲስአበባ ዩኒቨርሲቲ ናይ ምግብና ሳይንስን ኒውትሪሺን ማእከል ኣብ እንግሊዝ ሃገር ምስ ዝርከብ ኖቲንግሃም ዩኒቨርሲቲ ብምትሕብባር ካብ ዝሰራሕ ናይ ጂኦኒውትሪሽን ፕሮጀክት ጽንዓት ጉጅለ ኣባል እየ።ናይ ሕርሻ መሬት ስለዘለዎም ወይ እክሊ ስለዘምርቱ ኣብዚ ጽንዓት ንክሳተፉ ተዲዓሞም ኣለዉ። ኣብ ጽንትዓ ንምስታፍ ቅድሚ ምውሳኖምን ፍቃድ ምሃቦምን ስለጽንትዓ ላዓማን ካብኦም ትሳትፎ ንጽብዮ ክገልጸሎም። ኣብዚ ናይ ጽንዓት ፍቃድ ቅጥዒ ዉሽጢ ዘሎ ሓበሬታ ከንበሎም እየ። ግልጺ ዘይኮኑ ነግራት እንተተዛሪብ በይዘኦም ብዘይ ስጋኣት ይሕትቱኒ። ኣነ እውን እውን መልሲ ክህቦም እይ። ኣብዚ ናይ ጽንዓት ፍቃድ ቅጥዒ ዉሽጢ ዘለዉ ሓብሬታታት ምስ ካልኦት ሰባት ክምያየጥሎም ይክእሉ ጽእዮም። ተሳትፎኦም ሙሉእ ብሙሉእ ብዓርሰ ፍቃዶም ዝተመስረተ ኣዩ። ንምስታፍ እንተዘይ ተስማዕሚዖም ምንም ዓይነት ቅጽትዓ ኣይበጽሖምን።

What is the purpose of this study?The aim of this study is to understand how soil quality affects the nutritional value of cereal grains growing in those fields.

ናይቲ ጽንዓት ዓላማ እንታይኣዩ?
ናይዚ ጽንዓት እዙይ ላዓማ ናይ እኽሊ ምዓድን ትሕዝቶ ብናይ ሓምድ ዓይነት ወይ ጸባይ ከመይ ከምዝውሰን ንምርዳእ እዩ።

Why I have been chosen?You are randomly selected because you grow cereal grain within Malawi/Ethiopia. There is no specific reason other than this.

ኣነ ንምንታይ ተመሪጸ?
ናይ ሕርሻ መሬት ስለዘለዎም ወይ እኽሊ ስለዘምርቱ ኣብዚ ጽንትዓን ክሳተፉ ትምሪጾም። ነገር ግን ካብካልኦት ሓሮስቶት ብዝተፍልየ ዝትምረጹ ኮነ ተባሂሉን ብዝተፈለየ ምኽንያትን ዘይኮነስ ብኣጋጣሚ እይ።

What will participation involve?You will be asked questions about your field, soils, crops, and how you manage these. Your responses will be written down on a tablet. We will then ask to take a small sample of soil and cereal grain from your field (or crop store).

ኣብዚ ጽንዓት እንተተሳቲፈ ካባይ እንታይ ይጽብ?

ኣብዚ ጽንዓት እንተተሳቲፎም ብዛዕባ ሕርሻ ምሬት ናይ ሓመድ ዓይነት ወይ ጽባይን ብዛዕባ ዘምርትዎ ዓይነት እኽሊ ክጥይቖም እየ። መልሶም ኣብዛ ዝሓዝከ ዋታብሌት ክምዝግቦ እይ። ብተወሳኺ ካብሕርሻ ምሬቶም ወይ ቆፎኦም ንእሽተይ ናይ እኽልን ሓመድን ናሙና ክወስድ እይ።

What if I decide that I don't want to take part?You are free to decide that you don't want to take part in the study and can:
1. Refuse to answer any questions that you don't want to
2. Decide to stop the interview at any time
3. Remove your consent for the data collected to be used.

ኣብጽንዓትን ምስታፍ ፍቃድኛ እንተዘይ ኮይነስ?

ተሳትፎኦም ሙሉእ ብሙሉእ ኣብ ዓርሰ ፍቃዶም ዝተመስረተ እዩ። ስለዝኮነ
1.ክምልስዎ ዘይደለዩ ሕቶ ዘይክምልሱዎ ይካሉ እዮም
2. ቃለ መሕታቱ ኣብዝደለይዎ ግዜ ክቓርጽዎ ይክእሉ እዮም
3. ንምስታፍ ፍቃድኛ እንተኾይኖም ቃለ ምሕታት ድሕሪ ምግባሮም ፍቃደኝንቶም ክዐጽፉ ይክእሉ እዮም

Will I be paid for my time?There is no payment for taking part in this questionnaire.

ኣብጽንዓት እንትትሳተፍ ክኽፈለኒ ድዩ? ኣብ ጽንዓት ብምስታፎም ምንም ዓይነተ ክፍያ ኣይረክቡን።

Will I be anonymous, and who will know my identity?If you agree to take part in an interview, a Participant Number will be generated for you, and that's the only thing that will be used to identify you. Your identity will only be known by the interviewer, and will not be found in any record. Hard copy and electronic data will be stored on the University of Nottingham's computer network: thiswill be deleted after 7 years, or if you withdraw your consent (whichever is sooner).

ኣብ ጽንዓት እንተተሳቲፈ ዝሃብህዎ ሓብሬታ መን ክፈልጥ ይኽእል?

ኣብዚ ጽንዓት ንብስታፍ እንተተስማዕሚያም ዝሃብዎ ቃለም ጠይቅ ኮነ ናይ ሓምድን እኽልን ናሙና ብሽሞም ዘይኮነስ ብቁጽሪ ይውከል። ዝሃብዎ ሓብሬታ ቃለመጠይቅ ዝገበረሎም ሰብ ጥራሕ እዩ ዘይፈልጦ። ነገር ግን ኣብ ማንም ሰነድ ኣይርከብን። ናይ ወረቐት ሰነዳት ኮኑ ናይ ኤሌክትሮኒክስ ቅዳሕ ብ ኣዲስ ኣበባ ዩኒቨርሲቲ እና ኣብ እንግሊዝ ሃገር ዝርከብ ኖቲንግሃም ዩኒቨርሲቲ ጥራሕ ይቕምጥ። እቲ ጽንዓት ምስተወደኣ ወይ ኣብጽንዓት ንክሳተፉ ፍቃደኝንቶም እንተ ኣጺፎም ኮሎም ቅዳሓት ክጠፍኡ ይግበር።

Who shall I contact with any questions? Please contact the Principal Investigators, Redacted (Addis Ababa University, Center for Food Science and Nutrition; Redacted ; ስልክ Redacted); Redacted (University of Nottingham, UK; Redacted If you wish to ask questions or make a complaint you may also contact either Research Ethics Officer: Redacted (Office of the Associate Dean, Addis Ababa University; Redacted) or Redacted (University of Nottingham, UK; or Redacted

ሕቶ እንተሃሊኒ ናይ ቲጽንዓት ተጸዋዕቲ እንመን እዮም?

ምንም ዓይነት ሕቶ እንተሃሊዎም ኣብ ዝኾነ ሰዓት ክጥይቑ ይክእሉ እዮም። ብዛዕባ እዚ ጽንዓት ሕቶ እንተሃሊዎም Redacted ፣ ኣዲስ ኣባባ ዩኒቨርሲቲ ናይ ምግብና ሰይንስን ኒውትሪሽንን ማእከል (Redacted ; ስልክ Redacted) ክሓቱ ይክእሉ እዮም። ወይ ከኣ Redacted ፣ ኖቲንግሃም ዩኒቨርሲቲ (Redacted ; ስልኪ Redacted) ይሕተቱ።

ሕቶ ወይከዓ ቅሬታ እንተሃሊዎም/ወን Redacted (ናይ ምርምር ስነ-ምግባር ኦፌሰር፤ ተሓባባሪ ዲን ኣዲስ ኣባባ ዩኒቨርሲቲ/ Redacted) ወይከዓ Redacted ፣ ኖቲንግሃም ዩኒቨርሲቲ፤ Redacted) ይሕተቱ::

Reference number:Ethical Approval Number BIO-1819-001

Appendix B: Consent Form / Fomuyachilolezo

You have been invited to take part in a research project. If you like to participate, please sign or give your left thumb impression at the space indicated below.

ናይ ሕርሻ ምሬት ስለዘለዎም ወይ እኽሊ ስለዘምርቱ ኣብዚ ጽንዓት ንክሳተፉ ትዓዲሞም ኣልዉ። ኣብዚ ጽንዓት ንምስታፍ እንተተስሚያም ኣብታሕቲ ዘሎ ክፍቲ ቦታ ፊርሞኦም ወይ ናይ ጽጋማይ ዓባይ ኢዶም ዓሸራ የቐምጡ።

By completing this form you are consenting to take part in this research project; you can withdraw your consent at any point. To withdraw your consent, please either mention that to the Interviewer during the interview or contact Redacted (Addis Ababa University; Redacted) and/or Redacted (University of Nottingham, UK). If you wish to ask questions or make a complaint you may also contact either Research Ethics Officer: Redacted (Office of the Associate Dean, Addis Ababa University; Redacted) or Redacted (University of Nottingham, UK; or Redacted

ኣብዚ ናይ ጽንዓት ፍቃድ ቅጥዒ ስለዝፈረሙ ኣብዚ ጽንዓትን ምስታፍ ከምዝተስማዕምዑ ጌርና ንወስዶ። ነገር ግን ተሳትፎኦም ኣብ ዝኮነ ሰዓት ከቃርጽዎ ይክእሉ እዮም። ተሳትፎኦም ከቃርጹ እንተደልዮም Redacted (ሓጋዚፐሮፌሰር፣ ኣዲስ ኣባባ ዩኒቨርሲቲ) ወይ ከኣ Redacted ፣ ኖቲንግሃም ዩኒቨርሲቲ የፍልጡ።

ሕቶ ወይከዓ ቅሬታ እንተሃሊዎም/ወን Redacted (ናይ ምርምር ስነ-ምግባር ኦፊሰር፤ ተሓባባሪ ዲን ኣዲስ ኣባባ ዩኒቨርሲቲ/ Redacted) ወይከዓ Redacted ሞር፣ ኖቲንግሃም ዩኒቨርሲቲ፤ Redacted) ይሕተቱ። Redacted

Before signing this form, please read the following statements and indicate that you agree with them by initialling next to them.

ኣብዚ ቅጥዒ ቅድሚ ምፍራሞም ስዒቦም ዘለዉ ስጥቢታት ብምግንዛብ ምስምዕሞያም ንምግላጽ ኣብ ጎኒ ሕድሕድ ነጥቢ ናይ **'X'** ይግብሩ።

GID#	Tick here<br>ኣብዚ ምልክት<br>ኣቅምጡ(x)
I have been issued with a Participant Information Sheet<br><br>ብዛዕባ ጽንዓት ፍቃድ ቅጥዒ ገለጻ ተገይሩለይ	
I have been informed what the purpose of this research is, and the nature of the study<br><br>ናይቲ ጽንዓት ዓላማን ጠቅላላ ኩነታትን ገለጻ ተገይሩለይ	
I have been informed how the data that are collected within the research will be handled and stored.<br><br>ኣፍቲ ጽንዓት ዝእከቡ ሓበሬታታት ከመይ ከም ዝተሓጹን ከምዝቅመጡን ገለጻ ተገይሩለይ	

I have been informed that I can remove my consent at any time either during, or after the interview, and that withdrawal of consent will not harm me in any way.<br><br>ቃለመጠይቕ ኣብዝግበረሉ ሰዓት ኮነ ሓበሬታ ድሕሪ ምእካቡ ኣብ ጽንዓት ንምስታፍ ዝሃብክዎ ፍቃድ ምዕጻፍ ከም ጽኽእልን ምንም ዓይነት ሽግር ከምጽይ በጽሓኒን ተገሊጹለይ	
I have been informed that the interview will be written down on tablet<br><br>ንቃለመሕትት ዝሃብክዎም መልሲታት ኣብ ታብሌት ተጻሂፉ ከምዝቕመጥ ተነጊሩኒ	
I have been informed that my anonymised quotes may be used within the reporting of this research.<br><br>ንቃለመሕትት ዝሃብክዎም መልሲታት ኣብ ታብሌት ተጻሂፉ ከምዝቕመጥ ተነጊሩኒ	
I agree to take part in this study<br>ኣብጺ ጽንዓት ንምስታፍ ተስማዕሚዐ ኣለኩ	

Signed by...Date.....................

ፊርማ..ዕለት...................

Consent received by_________________Date______________

ቃለመሕትትኣካያዲ_______________________ዕለት________________
