# Supplementary material for: Cereal grain mineral micronutrient and soil chemistry data from GeoNutrition surveys in Ethiopia and Malawi
Source: Sci Data. 2022 Jul 25;9:443. doi: 10.1038/s41597-022-01500-5 (PMC9314434; doi:10.1038/s41597-022-01500-5)
Supplement: Supplementary file 14 — Supplementary file 1 [file 41597_2022_1500_MOESM14_ESM.pdf]

Supplementary file 2. Ethical approval, information sheet, and farmers' consent form in English and Chichewa for the Malawian GeoNutrition data collection.

Reference number: Ethical Approval Number BIO-1718-0004

School of Sociology & Social Policy

Application for Research Ethics Approval for Students and Staff

This form and any attachments must be completed, signed electronically, and submitted to Redacted

This application must be approved by the School of Sociology and Social Policy Research Ethics Committee (SSP-REC) before potential participants are approached to take part in any research. Any change in the design or conduct of the research over the course of the project should be reported to the SSP-REC and may require a new application for ethics approval. Please consult the School's Research Ethics website and reference where relevant the guidance on researcher safety, lone working, working abroad, the Mental Capacity Act 2005, research data management, etc.

Application Checklist

Your application cannot be considered without certain evidence. You should provide documents to cover each of the questions below where your answer is **'yes'**, and tick to indicate the type of evidence you have enclosed. All forms and templates can be found on the Research Ethics website.

Questions about your application	Evidence required	Enclosed
Does the research project, dissertation/thesis or assignment involve human participants or their data (even if you judge it to be of minimal risk)?	Application for Research Ethics Approval (this form)	☒
	Participant Consent Form	☒
	Participant Information Sheet	☒
Is the research of a sensitive nature, i.e. involves vulnerable participants and/or is concerned with a sensitive topic?	An exemplar of any communication inviting individuals to participate in the study	☐
	An indicative list of survey or interview questions to be used in the study	☐
Does the research involve data collection in the UK or overseas (even if you judge it to be of minimal risk)?<br>Overseas travel involving data collection is referred to the University's Insurance Office. Please consult the travel advice Flow Chart and Overseas Travel Guidance and **DO NOT** book travel or arrange fieldwork until your application has been approved.	Fieldwork Risk Assessment Form and Hazard Checklist	☒
If the research is to be conducted outside the UK or involves international partners, is ethics review required by non-UK RECs? (NB It is unlikely UG students will get ethical approval)	Non-UK REC approval	☐
Does the research require approval from a UK REC other than the SSP-REC (e.g., NHS, HMPPS, Social Care)?	External REC approval	☐
If you are a student, has your supervisor or tutor approved the project by signing this form (and any other documents requiring their signature)?	Supervisor or tutor signature	☐

Reference number: Ethical Approval Number BIO-1718-0004

**Section 1: Applicant details**	
Name of researcher	**Redacted**
Role	☐ Undergraduate student<br>☐ Postgraduate taught student<br>☐ Postgraduate research student<br>☒ Staff
Email address	**Redacted**
Names of other project members	See Appendix C

**To be completed by students only**	
Student ID number	
Degree programme	
Module name and number	
Supervisor, module leader or tutor	

**Section 2: Project details**	
Project title	**GeoNutrition (Work Package 1): Sampling soils and crops in Malawi**
Research question(s) or aim(s)	**Understanding the geospatial influence of soil quality on soil-to-grain transfer of micronutrients including iodine, iron, selenium and zinc**
Method(s) of data collection	**Questionnaire and soil and crop sampling from farmers' fields, to be conducted by multiple field teams employed by Lilongwe University of Agriculture and Natural Resources (LUANAR, Malawi). See Appendix D for Standard Operating Procedures, Questionnaire, Data Management/Use, etc. See Appendix E for map of approximate sampling locations.**
Where and when will data collection take place (please consult the University's Lone Working, Working Abroad and Safe Conduct of Fieldwork guidelines)?	**Late-April to early-June 2018 (Malawi)**
How will data be managed and used (please consult the University's Code of Research Conduct and Research Ethics and School Research Data Management Plan)?	**All data will be managed and used according to the University's Code of Research Conduct and Research Ethics and School of Biosciences.**
How will access to participants be gained?	**Participants will be selected by colleagues at Lilongwe University of Agriculture and Natural Resources (LUANAR, Malawi) after liaison with (1) national**

	**Government, (2) local authorities, (3) heads of households.**

**Section 3: Questions about the appropriate REC to review the application**	**Yes**	**No**
Does the study involve recruitment of patients or staff through the NHS or the use of NHS data or premises and/or equipment?	☐	☒
Does the study involve vulnerable adults who are unable to make an informed and free decision on their involvement in the research (e.g., those with a mental incapacity or prisoners) or involve children / adults who live within a social care setting?	☐	☒
If you have answered **'yes'** to either of the questions above, the SSP-REC may not be able to approve your project. You will need to send this completed form to the SSP-REC for reference and submit your research for ethics approval to the relevant external review board, e.g., HRA, HMPPS or SCREC. Once ethics approval is granted by the external review board, a copy should be sent to the SSP-REC for its records.<br><br>NB – for the purposes of research in the UK, an adult is a person aged 16 years or over.		

Section 4: Ethical considerations

Please answer **ALL** of the following questions by ticking the appropriate box and providing additional information in the text box where required.

**4.1: Questions about consent**	**Yes**	**No**
Will the research involve any participants who are known to be vulnerable due to: being aged under 16, residing in institutional care, having a learning disability, having a mental health condition, having physical or sensory impairments, previous life experiences (e.g., victims of abuse), other (please specify below)?	☐	☒
Will the study require the co-operation of a gatekeeper for initial access to the groups or individuals to be recruited (e.g., pupils at school, residents of nursing home)?	☐	☒
Will the research involve participants taking part without their knowledge or consent, deception or covert observation in any form?	☐	☒
Will the research involve access to personal information about identifiable individuals without their knowledge or consent?	☐	☒

If you have answered **'yes'** to any of the questions about consent, please explain why, and describe any steps you will take to deal with the ethical issues raised in the box below:

**4.2: Questions about the potential for harm**	**Yes**	**No**
If the research involves working with children and/or vulnerable adults, are there any reasons why you won't be able to follow the University's Guidance on the Safeguarding of Children and Adults at Risk?	☐	☒
Will the research expose participants to any risk of physical or emotional harm?	☐	☒

Will the research involve physically invasive procedures, the collection of bodily samples or the administering of drugs, placebos or other substances (e.g., vitamins, food)?	☐	☒
Will the research involve discussion of sensitive issues (e.g., abuse, sexual activity, sexuality, drug use, serious illness, commercial or legally sensitive topics)?	☐	☒
Will the research involve access to personal information about identifiable individuals without their knowledge or consent?	☐	☒
Will the research expose the researcher to any risk of physical or emotional harm?	☒	☐

If you have answered **'yes'** to any of the questions about the potential for harm, please explain why, and describe any steps you will take to deal with the ethical issues raised in the box below:

There is a minor risk of physical harm to researchers, associated with sampling soils and crops from farmers' fields. These risks are addressed in the Risk Assessment section of Appendix D. This Risk Assessment is a dynamic document.

**4.3: Questions about data collection**	**Yes**	**No**
Have you read the University's Research Code of Conduct guidelines, particularly section 4 on Data, and agree to abide by them?	☒	☐
Will it be made clear that participation is unlikely to be of direct personal benefit to the individual?	☒	☐
Will you refuse to offer incentives other than basic expenses such as cash payments and non-cash items such as vouchers or book tokens to potential participants as an inducement to participate in the research?	☒	☐
Before any data are collected, will participants be guaranteed anonymity only insofar as they do not disclose any illegal activities?	☒	☐
Before any data are collected, will anonymity not be guaranteed where there is disclosure or evidence of significant harm, abuse, neglect or danger to participants or to others?	☒	☐
If fieldwork is to take place in private or unfamiliar settings (i.e., not in public and/or professional spaces) have you detailed what steps will be taken to ensure your safety in the Fieldwork Risk Assessment Form and Hazard Checklist?	☒	☐
Will participants be informed when observation and/or recording is taking place?	☒	☐
Will participants be free to withdraw from the study at any time, including withdrawing collected data after an agreed timeframe?	☒	☐
Will participants be treated with dignity and respect at all times?	☒	☐
Will the participants be provided with your University contact details, and those of your supervisor, so that they may get in touch about any aspect of the research?	☒	☐
Will a signed copy of the consent form be retained by the researcher and another by the participant?	☒	☐

If you have answered **'no'** to any of the questions about data collection, please explain why, and describe any steps you will take to deal with the ethical issues raised in the box below:

4.4: Questions about data confidentiality and storage	Yes	No
Will data only be used for the purposes outlined within the Participant Consent Form and Information Sheet?	☒	☐
Where anonymity has been agreed with the participant, will data be anonymized as soon as possible after collection?	☒	☐
Will all personal data which could identify individual participants be inaccessible to everyone other than the researcher, their supervisor and internal and/or external examiners without participants' explicit consent?	☒	☐
Will you inform your supervisor and/or the School's REO and (if necessary) statutory services of any incidents of actual or suspected harm of children or vulnerable adults which are disclosed to you during the course of data collection?	☒	☐
Will participants be given the opportunity to know about the overall research findings?	☒	☐
Will all research data be managed and retained in accordance with the requirements of the University's Code of Research Conduct and Research Ethics and the School's Research Data Management Plan?	☒	☐

If you have answered **'no'** to any of the questions about data confidentiality and storage, please explain why, and describe any steps you will take to deal with the ethical issues raised in the box below:

Reference number: Ethical Approval Number BIO-1718-0004

Section 5: Ethical approval

DECLARATION OF ETHICAL RESEARCH

By signing this form I agree to work within the protocol which I have outlined and to abide by the University of Nottingham's Code of Research Ethics. If I make any changes to my protocol which would change my answers to any of the questions above I will submit a new form to my supervisor or module convener and to **Redacted**.

	**12th March 2018**
Signature of applicant	***Date***

AUTHORISATION

Having reviewed the ethical issues arising from the proposed research, I authorise the research to go ahead.

Signature of supervisor	***Date***

The School's Research Ethics Committee authorises the research to go ahead as described.

Signature of REC / REO	***Date***

Please remember to enclose all of the documentary evidence required to support your application, as indicated in the checklist on the front page of this application

Reference number: Ethical Approval Number BIO-1718-0004

Appendix A: Information Sheet for Participants / Tsamba la unthenga wachidziwitso kwa otenga nawo mbali: "GeoNutrition (Work Package 1): Sampling soils and crops in Malawi / Kutenga ma sampulo a dothi ndi mbewu ku Malawi"

Invitation: You are being invited to be involved in a research study; before you decide whether you want to take part, it is important for you to understand why the research is being done and what your participation will involve. Please read the following information carefully and discuss it with other people if you wish. Please contact me if anything is unclear or if you would like more information. Take time to decide whether or not you wish to take part.

Pempho: Mukupemphedwa kuti mutenge nawo gawo mu kafukufuku; koma musanasankhe kutero , ndikofunikira kwa inu kuti mumvetsetse cholinga chakafukufukuyu komanso zofunikira zakutenga nawo mbali kwanu. Chonde werengani unthenga otsatirawu moyenera ndi kukambirana ndi anzanu ngati mungafune kutero.Chonde ndiimbireni ngati pali chinachilichonse chomwe simukumvetsetsa kapena ngati mungafune unthenga owonjezera.Tengani nthawi yanu ndithu kulingalira musanasankhe kuvomera kutenga nawo gawo mu kafukufuku kapena kusatenga nawo mbali.

What is the purpose of this study? The aim of this study is to understand how soil quality affects the nutritional value of cereal grains growing in those fields.

Kodi cholinga chakafukufukuyu ndi chiyani? Cholinga chakafukufukuyu ndikufuna kumvetsetsetsa m'mene chonde cha dothi chimakhudzira mlingo wa thanzi ndi michere yamu njere za mbweu zolimbitsa thupi monga chimanga, mpunga, mapira, mchewere, mawere ndi tirigu zolimidwa mu dothilo.

Why I have been chosen? You are randomly selected because you grow cereal grain within Malawi/Ethiopia. There is no specific reason other than this.

Ndichifukwa chiyani ndasankhidwa? Mwasankhidwa mwachisawawa chifukwa chakuti mumalima mbewu za chakudya cholimbitsa thupi mu Malawi kapena Ethiopia. Palibenso chifukwa china chikhazikika kuposa ichi.

What will participation involve? You will be asked questions about your field, soils, crops, and how you manage these. Your responses will be written down on a tablet. We will then ask to take a small sample of soil and cereal grain from your field (or crop store).

Kutenga nawo mbali kwanga kufunika chiyani? Mudzafunsidwa mafunso okhudza munda wanu, mbewu zanu komanso njira zomwe mumazisamalira izi. Mayankho anu azalembedwa pa maikina a m'manja.. Tikatero tidzatenga nawo sampulo ya dothi ndi mbewu yanu ya chakudya cholimbitsa thupi kuchokera m'munda mwanu kapena mu nkhokwe yanu.

What if I decide that I don't want to take part? You are free to decide that you don't want to take part in the study and can:
1. Refuse to answer any questions that you don't want to
2. Decide to stop the interview at any time
3. Remove your consent for the data collected to be used.

Nanga nditasankha kusatenga nawo mbali? Muli ndi ufulu wosankha kusatenga nawo mbali mu kafukufukuyu ndipo mutha kuchitanso izi:

Reference number: Ethical Approval Number BIO-1718-0004

1. Kukana kuyankha mafunso amene simukufuna kuyankha
2. Kusankha kusiya kufunsidwaku pa nthawi ina iliyonse
3. Kuletsa chilolezo chomwe munapereka kuti unthenga omwe mwapeleka ukagwiritsidwe ntchito.

Will I be paid for my time? There is no payment for taking part in this questionnaire.

Kodi ndilipilidwa ku nthawi yanga yomwe ndipelekeyi? Kulibe malipilo ena aliwonse amene mupatsidwe chifukwa chotenga nawo mbali yoyankha mafunsowa.

Will I be anonymous, and who will know my identity? If you agree to take part in an interview, a Participant Number will be generated for you, and that's the only thing that will be used to identify you. Your identity will only be known by the interviewer, and will not be found in any record. Hard copy and electronic data will be stored on the University of Nottingham's computer network: this will be deleted after 7 years, or if you withdraw your consent (whichever is sooner).

Kodi sindidzatchulidwa dzina, komanso adzadziwe chizindikiro change ndi ndani? Ngati mwavomera kutenga nawo gawo loyankha mafunsowa, tidzapanga nambala yoimira otenga nawo mbali mukafukufukuyu ndipo ichi ndi chinthu chokhacho chomwe tidzagwiritse ntchito pofuna kuzindikira mayankkho anu. Chizindikiro chanu chidzadziwika ndi okufunsani mafunso yekha ndipo sizizapezeka muzolembedwa zina zilizonse. Uthenga wopelekedwa wamumapepala ndi wosungidwa mu makina udzasungidwa mu makina a compuyuta aku sukulu yaukachenjede ya Nottingham: unthengawu udzafufutidwa pakadzatha dzaka zisanu ndi ziwiri kapena nthawi ina iliyonse zisanakwane zakazi pamene inu mwasankha kulanda chilolezo chakuti unthenga omwe munapeleka usungidwe ndi kugwiritsidwa ntchito.

Who shall I contact with any questions? Please contact the Principal Investigators, Redacted (Lilongwe University of Agriculture and Natural Resources, LUANAR); Redacted (University of Nottingham, UK). Emails: Redacted. In the event of any complaint, please can you contact Redacted, PhD, Director of Research and Outreach, Lilongwe University of Agriculture and Natural Resources (LUANAR). Cell: Redacted; Email: Redacted.

Ngati ndili ndimafunso ndingalumikizane ndi ndani? Chonde lumikizanani ndi wotsogolera kafukufukuyu, a Redacted aku sukulu ya ukachenjede ya zaulimi (Lilongwe University of Agriculture and Natural Resources, LUANAR); Redacted a ku sukulu ya ukachenjede ya Nottingham (University of Nottingham, UK). Keyala: Redacted. Ngati mungakhale ndi dandaulo lirilonse, chonde lumikizananani ndi wamkulu wa kafukufuku ndi kufalitsa, Redacted ku sukulu ya ukachenjede ya zaulimi, Lilongwe University of Agriculture and Natural Resources (LUANAR). Keyala: Cell: Redacted; Email: Redacted.

Reference number: Ethical Approval Number BIO-1718-0004

Appendix B: Consent Form / Fomu yachilolezo

You have been invited to take part in a research project; in order to go forward with your participation, it is necessary for you to give your consent.

Mwasankhidwa kuti mutenge nawo gawo mukafukufuku; kuti mupitilire kutenga nawo mbali mukafukufukuyu, ndikoyenera kuti inu mupeleke chilolezo chanu.

By completing this form you are consenting to take part in this research project; you can withdraw your consent at any point. To withdraw your consent, please either mention that to the Interviewer during the interview or contact Redacted (Lilongwe University of Agriculture and Natural Resources, LUANAR) and/or Redacted Redacted (University of Nottingham, UK) **before 20 June 2018**.

Poyankha ndi kusayinira fomu iyi mukupeleka chilolezo chotenga nawo mbali mukafukufukuyu; koma mutha kulanda chilolezo chanu nthawi ina iliyonse. Ngati mwafuna kulanda chilolezo chanu, chonde afotokozereni okufunsani mafunso munthawi yomwe mukucheza nawoyo kapena lumikizanani ndi a Redacted aku sukulu ya ukachenjede ya zaulimi (Lilongwe University of Agriculture and Natural Resources, LUANAR); Redacted a ku sukulu ya ukachenjede ya Nottingham (University of Nottingham, UK) **pasanafike pa 20 June, 2018.**

Before signing this form, please read the following statements and indicate that you agree with them by initialling next to them.

Musanasayine fomu imeneyi, chonde werengani mfundo zotsatirazi ndikuonetsa kuti mukuvomereza poyika malembo oyambirira a mayina anu kutsogolo kwa mfundozi.

GID#	Tick here<br>Lembani<br>Chizindikiro<br>cha:<br>✓
I have been issued with a Participant Information Sheet<br><br>Ndapatsidwa pepala lopeleka unthenga kwa otenga nawo mbali	
I have been informed what the purpose of this research is, and the nature of the study<br><br>Ndadziwitsidwa cholinga chakafukufukuyu komanso ndondomeko yakafukufukuyu	
I have been informed how the data that are collected within the research will be handled and stored.<br><br>Ndadziwitsidwa kasamalidwe ndi kasungidwe ka unthenga womwe udzatoleledwe mukafufukuyu	
I have been informed that I can remove my consent at any time either during, or after the interview (up to 20 June 2018), and that withdrawal of consent will not harm me in	

any way.<br><br>Ndadziwitsidwa m’mene ndingalandire chilolezo chomwe ndingapeleke mu nthawi ina iliyonse yoyankha mafunsowa kapena pasanafike pa (20 June 2018) ndipo chiletso changa sichidzakhala ndi vuto liri lonse pa ine	
I have been informed that the interview will be written down on tablet<br><br>Ndadziwitsidwa kuti unthenga womwe ndipeleke mumachezawa ulembedwa mumakina a m’manja	
I have been informed that my anonymised quotes may be used within the reporting of this research.<br><br>Ndadziwitsidwa kuti zoyankhula zanga zenizeni zitha kugwiritsidwa ntchito mu malipoti akafukufukuyu koma sindidzatchulidwa dzina	
I agree to take part in this study<br><br>Ndavomera kutenga nawo mbali mukafukufukuyu	

Signed by……………………………………………Date…………………

Wosayina……………………………………………Tsiku………………..

Consent received by……………………………….Date…………………

Wotenga chiloleza…………………………………Tsiku………………

Reference number: Ethical Approval Number BIO-1718-0004

Appendix C: Participant List

Name	Job Title and Institution	Email	Institute	Country	Management Role	Project Role
Dawd Gashu	Assistant Professor, Center for Food Science and Nutrition, Addis Ababa University		AAU	ETH	AAU Lead	Nutritionist
Tegbaru Bellete	EthioSIS Project Team Leader, Ethiopian Agricultural Transformation Agency		ATA/MoA	ETH	ATA Lead	Soil Scientist
Kiflu Gudeta	Senior GIS Expert, Acting Director at Ministry of Agriculture and Natural Resource, Ministry of Agriculture and Natural Resource, Ethiopia		ATA/MoA	ETH		GIS
Sam Gameda	Senior Soil Scientist, International Maize and Wheat Improvement Center		CIMMYT	ETH	CIMMYT Lead	Agronomist / Soil Scientist
Adamu Belay	Nutritionist, Ethiopian Public Health Institute		EPHI	ETH	EPHI Lead	Nutritionist
Dilnesaw Zerfu	Associate Researcher, Ethiopian Public Health Institute		EPHI	ETH		Nutritionist
Solomon Eshetu	Food Science and Nutrition Research Directorate, Ethiopian Public Health Institute		EPHI	ETH		Nutritionist
Tesfaye Hailu	Nutritionist, Ethiopian Public Health Institute		EPHI	ETH		Advisor
Tilahun Amede	Principal Scientist, Natural Resources and Systems Agronomy, International Crops Research Institute for the Semi-Arid Tropics (ICRISAT), Ethiopia		ICRISAT	ETH	ICRISAT Lead	Agronomist / Soil Scientist
Louise Ander	Environmental Process Geochemist, British Geological Survey		BGS	GBR	BGS Lead	Data management / training
Edward Joy	Research Fellow in Nutrition and Sustainability, London School of Hygiene & Tropical Medicine		LSHTM	GBR	LSHTM Lead	Nutritionist / macroeconomics
Alan Dangour	Professor of Nutrition, London School of Hygiene & Tropical Medicine		LSHTM	GBR		Nutritionist
Elaine Ferguson	Senior Lecturer, London School of Hygiene & Tropical Medicine		LSHTM	GBR		Nutritionist
Joanna Sturgess	Research Fellow, London School of Hygiene and Tropical Medicine		LSHTM	GBR		Nutritionist / logistics
Kirsten Dawes	Project Administrator, London School of Hygiene and Tropical Medicine		LSHTM	GBR		Research administration
Liz Allen	Professor, Epidemiology and Statistics, London School of Hygiene & Tropical Medicine		LSHTM	GBR		Epidemiology / statistics
Steve McGrath	Professor of Sustainable Agricultural Sciences, Rothamsted Research		RRes	GBR	RRes/AfSIS Lead	Soil Scientist
Alice Milne	Spatial Statistician, Sustainable Agricultural Sciences, Rothamsted Research	Redacted	RRes	GBR		Geostatistics
Jackie Stroud	Research Scientist, Sustainable Agricultural Sciences, Rothamsted Research		RRes	GBR		Soil Scientist
Stephan Haefele	Systems Agronomist, Sustainable Agriculture Sciences, Rothamsted Research		RRes	GBR		Agronomist
Martin Broadley	Professor of Plant Nutrition, University of Nottingham		UoN	GBR	UoN/Overall lead	Project manager, crop nutrition
Diriba Kumssa	Research Fellow, GIS, University of Nottingham		UoN	GBR		GIS / data management / web manag
Joanna Smuga Lumatz	Project Administrator, University of Nottingham		UoN	GBR		Research administration
Kate Millar	Bioethicist and Director of Centre for Applied Bioethics, University of Nottingham		UoN	GBR		Bioethics
Liz Bailey	Lecturer in Environmental Science, University of Nottingham		UoN	GBR		Soil Chemist
Lolita Wilson	Laboratory Manager, University of Nottingham		UoN	GBR		Senior technician
Abdul Mossa	Senior Technician, University of Nottingham		UoN	GBR		Senior technician
Murray Lark	Geoinformatics, University of Nottingham		UoN	GBR		Geostatistics
Scott Young	Associate Professor and Reader in Environmental Science, University of Nottingham		UoN	GBR		Soil Chemist
Simon Langley-Evans	Professor of Human Nutrition, University of Nottingham		UoN	GBR		Nutritionist
Hugo de Groote	Agricultural Economist/Principal Scientist, International Maize and Wheat Improvement Center (CIMMYT), Kenya		CIMMYT	KEN		Economist
Ermias Betemariam	Landscape Ecologist, World Agroforestry Centre (ICRAF)		ICRAF	KEN		Ecologist
Keith Shepherd	Principal Soil Scientist, World Agroforestry Centre		ICRAF	KEN		Soil Scientist
Ben Chilima	Deputy Director, Community Health Services, Ministry of Health, Malawi		CHSU	MWI	CHSU Lead	Public Health Policy
Wilkson Makumba	Director of Department of Agricultural Research Services, Malawi Ministry of Agriculture		DARS	MWI	DARS Lead	Agricultural Research Services Lead
Moses Munthali	Senior Soil and Plant Nutrition Scientist, Malawi Ministry of Agriculture		DARS	MWI		Soil Scientist
Ivy Ligowe	Research Scientist, Dept. Agricultural Research Services; PhD Student, Dept. Crop & Soil Sciences, Lilongwe University of Agriculture & Natural Resources		DARS/LUANAF	MWI		Soil Scientist
Felix Phiri	Director of Nutrition, the Department of Nutrition, HIV and AIDS, Ministry of Health, Malawi		DNHA	MWI	DNHA Lead	Nutrition Policy
Patson Nalivata	Department of Crop and Soil Sciences, Lilongwe University of Agriculture and Natural Resources		LUANAR	MWI	LUANAR Lead	Soil Scientist
Alexander Kalimbira	Human Nutritionist, Head, Department of Human Nutrition and Health		LUANAR	MWI		Nutritionist
Allan Chilimba	Agriculture and Soils Consultant, Malawi		N/A	MWI		Soil Scientist
Joseph Mfutso-Bengo	Professor of Bioethics and Director of Centre for Bioethics at the College of Medicine at the University of Malawi		UNIMA	MWI	UNIMA Lead	Bioethics
